# Supplementary material for: Unequal cluster sizes in stepped-wedge cluster randomised trials: a systematic review
Source: BMJ Open. 2017 Nov 15;7(11):e017151. doi: 10.1136/bmjopen-2017-017151 (PMC5695383; doi:10.1136/bmjopen-2017-017151)
Supplement: Supplementary file 1 [file bmjopen-2017-017151supp001.pdf]

SYSTEMATIC REVIEW SEARCH STRATEGY

1. step$ wedge.ti,ab.
2. experimentally staged introduction.ti,ab.
3. delayed intervention.ti,ab.
4. (one directional cross over design or one directional crossover design).ti,ab.
5. ((incremental or phased or staggered or stepwise or step wise or delayed) adj1 (recruitment or introduction or implementation)).ti,ab.
6. or/1-5
7. limit 6 to english language
8. limit 7 to randomized controlled trial
9. limit 8 to humans
10. limit 9 to yr=”2014-current”
