# Supplementary material for: Unequal cluster sizes in stepped-wedge cluster randomised trials: a systematic review
Source: BMJ Open. 2017 Nov 15;7(11):e017151. doi: 10.1136/bmjopen-2017-017151 (PMC5695383; doi:10.1136/bmjopen-2017-017151)
Supplement: Supplementary file 2 [file bmjopen-2017-017151supp002.pdf]

Reference list of included studies

1. Craine N, Whitaker R, Perrett S, Zou L, Hickman M, Lyons M. A stepped wedge cluster randomized control trial of dried blood spot testing to improve the uptake of hepatitis C antibody testing within UK prisons. European journal of public health. 2014.

2. Palmay L, Elligsen M, Walker SAN, Pinto R, Walker S, Einarson T, et al. Hospital-wide rollout of antimicrobial stewardship: A stepped-wedge randomized trial. Clinical Infectious Diseases. 2014;59(6):867-74.

3. van de Steeg L, R IJ, Langelaan M, Wagner C. Can an e-learning course improve nursing care for older people at risk of delirium: a stepped wedge cluster randomised trial. BMC geriatrics. 2014;14:69.

4. Haugen AS, Softeland E, Almeland SK, Sevdalis N, Vonen B, Eide GE, et al. Effect of the World Health Organization Checklist on Patient Outcomes: A Stepped Wedge Cluster Randomized Controlled Trial. Annals of surgery. 2014.

5. Stern A, Mitsakakis N, Paulden M, Alibhai S, Wong J, Tomlinson G, et al. Pressure ulcer multidisciplinary teams via telemedicine: a pragmatic cluster randomized stepped wedge trial in long term care. BMC Health Serv Res. 2014;14:83.

6. Enns E, Rhemtulla R, Ewa V, Fruetel K, Holroyd-Leduc JM. A controlled quality improvement trial to reduce the use of physical restraints in older hospitalized adults. Journal of the American Geriatrics Society. 2014;62(3):541-5.

7. Roy A, Anaraki S, Hardelid P, Catchpole M, Rodrigues LC, Lipman M, et al. Universal HIV testing in London tuberculosis clinics: A cluster randomised controlled trial. European Respiratory Journal. 2013;41(3):627-34.

8. Mhurchu CN, Gorton D, Turley M, Jiang Y, Michie J, Maddison R, et al. Effects of a free school breakfast programme on children's attendance, academic achievement and short-term hunger: Results from a stepped-wedge, cluster randomised controlled trial. Journal of Epidemiology and Community Health. 2013;67(3):257-64.

9. Leontjevas R, Gerritsen DL, Smalbrugge M, Teerenstra S, Vernooij-Dassen MJFJ, Koopmans RTCM. A structural multidisciplinary approach to depression management in nursing-home residents: A multicentre, stepped-wedge cluster-randomised trial. The Lancet. 2013;381(9885):2255-64.

10. Gruber JS, Reygadas F, Arnold BF, Ray I, Nelson K, Colford Jr JM. A stepped wedge, cluster-randomized trial of a household UV-disinfection and safe storage drinking water intervention in rural Baja California Sur, Mexico. American Journal of Tropical Medicine and Hygiene. 2013;89(2):238-45.

11. Durovni B, Saraceni V, Moulton LH, Pacheco AG, Cavalcante SC, King BS, et al. Effect of improved tuberculosis screening and isoniazid preventive therapy on incidence of tuberculosis and death in patients with HIV in clinics in Rio de Janeiro, Brazil: A stepped wedge, cluster-randomised trial. The Lancet Infectious Diseases. 2013;13(10):852-8.

12. Bashour HN, Kanaan M, Kharouf MH, Abdulsalam AA, Tabbaa MA, Cheikha SA. The effect of training doctors in communication skills on women's satisfaction with doctor-woman relationship during labour and delivery: A stepped wedge cluster randomised trial in Damascus. BMJ Open. 2013;3(8).

13. van den Broek IV, van Bergen JE, Brouwers EE, Fennema JS, Gotz HM, Hoebe CJ, et al. Effectiveness of yearly, register based screening for chlamydia in the Netherlands: Controlled trial with randomised stepped wedge implementation. BMJ: British Medical Journal. 2012;345(7869):1-12.

14. Monse B, Duijster D, Sheiham A, Grijalva-Eternod CS, van Palenstein Helderman W, Hobdell MH. The effects of extraction of pulpally involved primary teeth on weight, height and BMI in underweight Filipino children. A cluster randomized clinical trial. BMC public health. 2012;12:725.

15. Horner C, Wilcox M, Barr B, Hall D, Hodgson G, Parnell P, et al. The longitudinal prevalence of MRSA in care home residents and the effectiveness of improving infection prevention knowledge and practice on colonisation using a stepped wedge study design. BMJ Open. 2012;2(1):e000423.

16. Fuller C, Michie S, Savage J, McAteer J, Besser S, Charlett A, et al. The Feedback Intervention Trial (FIT)--improving hand-hygiene compliance in UK healthcare workers: a stepped wedge cluster randomised controlled trial. PloS one. 2012;7(10):e41617.

17. Chinbuah MA, Kager PA, Abbey M, Gyapong M, Awini E, Nonvignon J, et al. Impact of community management of fever (using antimalarials with or without antibiotics) on childhood mortality: A cluster-randomized controlled trial in Ghana. American Journal of Tropical Medicine and Hygiene. 2012;87(SUPPL.5):11-20.

18. Etchells E, Adhikari NK, Wu R, Cheung M, Quan S, Mraz R, et al. Real-time automated paging and decision support for critical laboratory abnormalities. BMJ quality & safety. 2011;20(11):924-30.

19. Bacchieri G, Barros AJ, Santos JV, Goncalves H, Gigante DP. A community intervention to prevent traffic accidents among bicycle commuters. Revista de saude publica. 2010;44(5):867-75.

20. Solomon E, Rees T, Ukoumunne OC, Metcalf B, Hillsdon M. The Devon Active Villages Evaluation (DAVE) trial of a community-level physical activity intervention in rural south-west England: A stepped wedge cluster randomised controlled trial. The International Journal of Behavioral Nutrition and Physical Activity. 2014;11:94.

21. Gozalo P, Prakash S, Qato DM, Sloane PD, Mor V. Effect of the bathing without a battle training intervention on bathing-associated physical and verbal outcomes in nursing home residents with dementia: A randomized crossover diffusion study. Journal of the American Geriatrics Society. 2014;62(5):797-804.

22. Foster JM, Hoskins G, Smith B, Lee AJ, Price D, Pinnock H. Practice development plans to improve the primary care management of acute asthma: randomised controlled trial. BMC Fam Pract. 2007;8:23.

23. Flannery DJ, Vazsonyi AT, Liau AK, Guo S, Powell KE, Atha H, et al. Initial behavior outcomes for the peacebuilders universal school-based violence prevention program. Developmental psychology. 2003;39(2):292-308.

24. Priestley G, Watson W, Rashidian A, Mozley C, Russell D, Wilson J, et al. Introducing Critical Care Outreach: A ward-randomised trial of phased introduction in a general hospital. Intensive Care Medicine. 2004;30(7):1398-404.

25. Husaini BA, Reece MC, Emerson JS, Scales S, Hull PC, Levine RS. A church-based program on prostate cancer screening for African American men: reducing health disparities. Ethn Dis. 2008;18(2 Suppl 2):S2-179-84.

26. Viera AJ, Garrett JM. Preliminary study of a school-based program to improve hypertension awareness in the community. Family medicine. 2008;40(4):264-70.

27. Barton A, Basham M, Foy C, Buckingham K, Somervill M. The Watcombe Housing Study: the short term effect of improving housing conditions on the health of residents. J Epidemiol Community Health. 2007;61(9):771-7.

28. Bailet LL, Repper KK, Piasta SB, Murphy SP. Emergent literacy intervention for prekindergarteners at risk for reading failure. Journal of Learning Disabilities. 2009;42(4):336-55.

29. Weiner M, El Hoyek G, Wang L, Dexter PR, Zerr AD, Perkins AJ, et al. A web-based generalist-specialist system to improve scheduling of outpatient specialty consultations in an academic center. Journal of general internal medicine. 2009;24(6):710-5.

30. Brown BB, Young J, Smith DP, Kneebone AB, Brooks AJ, Xhilaga M, et al. Clinician-led improvement in cancer care (CLICC)--testing a multifaceted implementation strategy to increase evidence-based prostate cancer care: phased randomised controlled trial--study protocol. Implementation science : IS. 2014;9:64.

31. Reuther S, Holle D, Buscher I, Dortmann O, Muller R, Bartholomeyczik S, et al. Effect evaluation of two types of dementia-specific case conferences in German nursing homes (FallDem) using a stepped-wedge design: Study protocol for a randomized controlled trial. Trials. 2014;15(1).

32. Bernabe-Ortiz A, Diez-Canseco F, Gilman RH, Cardenas MK, Sacksteder KA, Miranda JJ. Launching a salt substitute to reduce blood pressure at the population level: A cluster randomized stepped wedge trial in Peru. Trials. 2014;15(1).

33. Brimblecombe J, Ferguson M, Liberato SC, Ball K, Moodie ML, Magnus A, et al. Stores Healthy Options Project in Remote Indigenous Communities (SHOP@RIC): a protocol of a randomised trial promoting healthy food and beverage purchases through price discounts and in-store nutrition education. BMC Public Health. 2013;13:744.

34. Feng R, Li K, Cheng J, Xie S, Chai J, Wei P, et al. Toward integrated and sustainable prevention against diabetes in rural China: Study rationale and protocol of eCROPS. BMC Endocrine Disorders. 2013;13(28).

35. Keriel-Gascou M, Buchet-Poyau K, Duclos A, Rabilloud M, Figon S, Dubois JP, et al. Evaluation of an interactive program for preventing adverse drug events in primary care: study protocol of the InPAct cluster randomised stepped wedge trial. Implementation science : IS. 2013;8:69.

36. Praveen D, Patel A, McMahon S, Prabhakaran D, Clifford GD, Maulik PK, et al. A multifaceted strategy using mobile technology to assist rural primary healthcare doctors and frontline health workers in cardiovascular disease risk management: protocol for the SMARTHealth India cluster randomised controlled trial. Implement Sci. 2013;8:137.

37. Ward J, McGregor S, Guy RJ, Rumbold AR, Garton L, Silver BJ, et al. STI in remote communities: improved and enhanced primary health care (STRIVE) study protocol: a cluster randomised controlled trial comparing 'usual practice' STI care to enhanced care in remote primary health care services in Australia. BMC Infect Dis. 2013;13:425.

38. Bennett PN, Daly RM, Fraser SF, Haines T, Barnard R, Ockerby C, et al. The impact of an exercise physiologist coordinated resistance exercise program on the physical function of people receiving hemodialysis: a stepped wedge randomised control study. BMC Nephrol. 2013;14:204.

39. Poldervaart JM, Reitsma JB, Koffijberg H, Backus BE, Six AJ, Doevendans PA, et al. The impact of the HEART risk score in the early assessment of patients with acute chest pain: design of a stepped wedge, cluster randomised trial. BMC Cardiovasc Disord. 2013;13:77.

40. Rasmussen CD, Holtermann A, Mortensen OS, Sogaard K, Jorgensen MB. Prevention of low back pain and its consequences among nurses' aides in elderly care: a stepped-wedge multi-faceted cluster-randomized controlled trial. BMC Public Health. 2013;13:1088.

41. van Holland BJ, de Boer MR, Brouwer S, Soer R, Reneman MF. Sustained employability of workers in a production environment: design of a stepped wedge trial to evaluate effectiveness and cost-benefit of the POSE program. BMC Public Health. 2012;12:1003.

42. Dreischulte T, Grant A, Donnan P, McCowan C, Davey P, Petrie D, et al. A cluster randomised stepped wedge trial to evaluate the effectiveness of a multifaceted information technology-based intervention in reducing high-risk prescribing of non-steroidal anti-inflammatory drugs and antiplatelets in primary medical care: the DQIP study protocol. Implementation science : IS. 2012;7:24.

43. Flocke SA, Antognoli E, Step MM, Marsh S, Parran T, Mason MJ. A Teachable Moment Communication Process for smoking cessation talk: description of a group randomized clinician-focused intervention. BMC health services research. 2012;12:109.

44. Marshall T, Caley M, Hemming K, Gill P, Gale N, Jolly K. Mixed methods evaluation of targeted case finding for cardiovascular disease prevention using a stepped wedged cluster RCT. BMC public health. 2012;12:908.

45. Mouchoux C, Rippert P, Duclos A, Fassier T, Bonnefoy M, Comte B, et al. Impact of a multifaceted program to prevent postoperative delirium in the elderly: the CONFUCIUS stepped wedge protocol. BMC geriatrics. 2011;11:25.

46. Turner J, Kelly B, Clarke D, Yates P, Aranda S, Jolley D, et al. A randomised trial of a psychosocial intervention for cancer patients integrated into routine care: the PROMPT study (promoting optimal outcomes in mood through tailored psychosocial therapies). BMC Cancer. 2011;11:48.

47. Liddy C, Hogg W, Russell G, Wells G, Armstrong CD, Akbari A, et al. Improved delivery of cardiovascular care (IDOCC) through outreach facilitation: study protocol and implementation details of a cluster randomized controlled trial in primary care. Implement Sci. 2011;6:110.

48. The Gambia Hepatitis Intervention Study. The Gambia Hepatitis Study Group. Cancer Research. 1987;47(21):5782-7.

49. Schultz TJ, Kitson AL, Soenen S, Long L, Shanks A, Wiechula R, et al. Does a multidisciplinary nutritional intervention prevent nutritional decline in hospital patients? A stepped wedge randomised cluster trial. e-SPEN Journal. 2014;9(2):e84-e90.

50. Kjeken I, Berdal G, Bo I, Dager T, Dingsor A, Hagfors J, et al. Evaluation of a structured goal planning and tailored follow-up programme in rehabilitation for patients with rheumatic diseases: protocol for a pragmatic, stepped-wedge cluster randomized trial. BMC musculoskeletal disorders. 2014;15:153.

51. Stringer JS, Chisembele-Taylor A, Chibwesha CJ, Chi HF, Ayles H, Manda H, et al. Protocol-driven primary care and community linkages to improve population health in rural Zambia: the Better Health Outcomes through Mentoring and Assessment (BHOMA) project. BMC Health Serv Res. 2013;13 Suppl 2:S7.

52. De Allegri M, Pokhrel S, Becher H, Dong H, Mansmann U, Kouyate B, et al. Step-wedge cluster-randomised community-based trials: an application to the study of the impact of community health insurance. Health research policy and systems / BioMed Central. 2008;6:10.

53. Gucciardi E, Fortugno M, Horodezny S, Lou W, Sidani S, Espin S, et al. Will Mobile Diabetes Education Teams (MDETs) in primary care improve patient care processes and health outcomes? Study protocol for a randomized controlled trial. Trials. 2012;13:165.

54. Williams AL, Phillips CJ, Watkins A, Rushton AB. The effect of work-based mentoring on patient outcome in musculoskeletal physiotherapy: study protocol for a randomised controlled trial. Trials. 2014;15:409.

55. Mosha F, Winani S, Wood S, Changalucha J, Ngasalla B. Evaluation of the effectiveness of a clean delivery kit intervention in preventing cord infection and puerperal sepsis among neonates and their mothers in rural Mwanza Region, Tanzania. Tanzania health research bulletin. 2005;7(3):185-8.

56. Strijbos MJ, Steunenberg B, van der Mast RC, Inouye SK, Schuurmans MJ. Design and methods of the Hospital Elder Life Program (HELP), a multicomponent targeted intervention to prevent delirium in hospitalized older patients: efficacy and cost-effectiveness in Dutch health care. BMC Geriatr. 2013;13:78.

57. Zwijsen SA, Smalbrugge M, Eefsting JA, Twisk JWR, Gerritsen DL, Pot AM, et al. Coming to grips with challenging behavior: A cluster randomized controlled trial on the effects of a multidisciplinary care program for challenging behavior in Dementia. Journal of the American Medical Directors Association. 2014;15(7):531.e1-.e10.

58. Baker C, Huxley P, Dennis M, Islam S, Russell I. Alleviating staff stress in care homes for people with dementia: Protocol for stepped-wedge cluster randomised trial to evaluate a web-based mindfulness- stress reduction course. BMC Psychiatry 2015 12;15.

59. Baker M, Biringen Z, Meyer-Parsons B, Schneider A. Emotional attachment and emotional availability tele-intervention for adoptive families. Infant Ment Health J 2015;36(2):179-192.

60. Banga FR, Truijens SEM, Fransen AF, Dieleman JP, Van Runnard Heimel PJ, Oei GS. The impact of transmural multiprofessional simulation-based obstetric team training on perinatal outcome and quality of care in the Netherlands. BMC Med Educ 2014;14(1).

61. Brady MC, Stott D, Weir CJ, Chalmers C, Sweeney P, Donaldson C, et al. Clinical and cost effectiveness of enhanced oral healthcare in stroke care settings (SOCLE II): A pilot, stepped wedge, cluster randomized, controlled trial protocol. Int J Stroke 2015;10(6):979-984.

62. Britton B, McCarter K, Baker A, Wolfenden L, Wratten C, Bauer J, et al. Eating As Treatment (EAT) study protocol: A stepped-wedge, randomised controlled trial of a health behaviour change intervention provided by dietitians to improve nutrition in patients with head and neck cancer undergoing radiotherapy. BMJ Open 2015;5(7).

63. Chavane L, Merialdi M, Betrán AP, Requejo-Harris J, Bergel E, Aleman A, et al. Implementation of evidence-based antenatal care in Mozambique: A cluster randomized controlled trial: Study protocol. BMC Health Serv Res 2014;14(1).

64. Cowan JF, Micek M, Cowan JFG, Napua M, Hoek R, Gimbel S, et al. Early ART initiation among HIV-positive pregnant women in central Mozambique: a stepped wedge randomized controlled trial of an optimized Option B+ approach. Implementation Science 2015;10:61.

65. Dryden-Peterson S, Bennett K, Hughes MD, Veres A, John O, Pradhananga R, et al. An augmented SMS intervention to improve access to antenatal CD4 testing and ART initiation in HIV-infected pregnant women: A cluster randomized trial. PLoS ONE 2015;10(2).

66. Durovni B, Saraceni V, van den Hof S, Trajman A, Cordeiro-Santos M, Cavalcante S, et al. Impact of Replacing Smear Microscopy with Xpert MTB/RIF for Diagnosing Tuberculosis in Brazil: A Stepped-Wedge Cluster-Randomized Trial. PLoS Med 2014;11(12).

67. Fiscella K, Ogedegbe G, He H, Carroll J, Cassells A, Sanders M, et al. Blood Pressure Visit Intensification Study in Treatment: Trial design. Am Heart J 2015;170(6):1202-1210.

68. Golden MR, Kerani RP, Stenger M, Hughes JP, Aubin M, Malinski C, et al. Uptake and Population-Level Impact of Expedited Partner Therapy (EPT) on Chlamydia trachomatis and Neisseria gonorrhoeae: The Washington State Community-Level Randomized Trial of EPT. PLoS Med 2015;12(1):1-22.

69. Grace AM, Lippert S, Collins K, Pineda N, Tolani A, Walker R, et al. Educating health care professionals on human trafficking. Pediatr Emerg Care 2014;30(12):856-861.

70. Haines TP, O'Brien L, Mitchell D, Bowles K-, Haas R, Markham D, et al. Study protocol for two randomized controlled trials examining the effectiveness and safety of current weekend allied health services and a new stakeholder-driven model for acute medical/surgical patients versus no weekend allied health services. Trials 2015;16(1).

71. Happ MB, Sereika SM, Houze MP, Seaman JB, Tate JA, Nilsen ML, et al. Quality of care and resource use among mechanically ventilated patients before and after an intervention to assist nurse-nonvocal patient communication. Heart Lung J Acute Crit Care 2015;44(5):408-415.e2.

72. Hayden MK, Lin MY, Lolans K, Weiner S, Blom D, Moore NM, et al. Prevention of colonization and infection by klebsiella pneumoniae carbapenemase-producing enterobacteriaceae in long-term acute-care hospitals. Clin Infect Dis 2015;60(8):1154-1161.

73. Helitzer DL, Hathorn G, Benally J, Ortega C. Culturally relevant model program to prevent and reduce agricultural injuries. J Agric Saf Health 2014;20(3):175-198.

74. Hill A-, McPhail SM, Waldron N, Etherton-Beer C, Ingram K, Flicker L, et al. Fall rates in hospital rehabilitation units after individualised patient and staff education programmes: A pragmatic, stepped-wedge, cluster-randomised controlled trial. Lancet 2015;385(9987):2592-2599.

75. Hunter SB, Ober AJ, Paddock SM, Hunt PE, Levan D. Continuous quality improvement (CQI) in addiction treatment settings: design and intervention protocol of a group randomized pilot study. Addict Sci Clin Pract 2014;9:4.

76. Jarvik JG, Comstock BA, James KT, Avins AL, Bresnahan BW, Deyo RA, et al. Lumbar Imaging With Reporting Of Epidemiology (LIRE)-Protocol for a pragmatic cluster randomized trial. Contemp Clin Trials 2015;45:157-163.

77. Jordan S, Gabe-Walters ME, Watkins A, Humphreys I, Newson L, Snelgrove S, et al. Nurse-led medicines' monitoring for patients with dementia in care homes: A pragmatic cohort stepped wedge cluster randomised trial. PLoS ONE 2015;10(10).

78. Kelly PJ, Baker AL, Deane FP, Callister R, Collins CE, Oldmeadow C, et al. Study protocol: a stepped wedge cluster randomised controlled trial of a healthy lifestyle intervention for people attending residential substance abuse treatment. BMC Public Health 2015;15:465.

79. Li S, Wu Y, Du X, Li X, Patel A, Peterson ED, et al. Rational and design of a stepped-wedge cluster randomized trial evaluating quality improvement initiative for reducing cardiovascular events among patients with acute coronary syndromes in resource-constrained hospitals in China. Am Heart J 2015;169(3):349-355.

80. Marrin K, Wood F, Firth J, Kinsey K, Edwards A, Brain KE, et al. Option Grids to facilitate shared decision making for patients with Osteoarthritis of the knee: Protocol for a single site, efficacy trial. BMC Health Serv Res 2014;14(1).

81. Morrison LJ, Brooks SC, Dainty KN, Dorian P, Needham DM, Ferguson ND, et al. Improving use of targeted temperature management after out-of-hospital cardiac arrest: A stepped wedge cluster randomized controlled trial. Crit Care Med 2015;43(5):954-964.

82. Noel PH, Romero RL, Robertson M, Parchman ML. Key activities used by community based primary care practices to improve the quality of diabetes care in response to practice facilitation. Quality in Primary Care 2014;22(4):211-219.

83. Nosyk B, Krebs E, Min JE, Ahamad K, Buxton J, Goldsmith C, et al. The 'Expanded HIV care in opioid substitution treatment' (EHOST) cluster-randomized, stepped-wedge trial: A study protocol. Contemp Clin Trials 2015;45:201-209.

84. Olds DL, Baca P, McClatchey M, Ingoldsby EM, Luckey DW, Knudtson MD, et al. Cluster randomized controlled trial of intervention to increase participant retention and completed home visits in the Nurse-Family Partnership. Prevention Science 2015 08;16(6):778-788.

85. Palmer VJ, Chondros P, Piper D, Callander R, Weavell W, Godbee K, et al. The CORE study protocol: A stepped wedge cluster randomised controlled trial to test a co-design technique to optimise psychosocial recovery outcomes for people affected by mental illness in the community mental health setting. BMJ Open 2015;5(3).

86. Pickering BW, Dong Y, Ahmed A, Giri J, Kilickaya O, Gupta A, et al. The implementation of clinician designed, human-centered electronic medical record viewer in the intensive care unit: A pilot step-wedge cluster randomized trial. Int J Med Informatics 2016;84(5):299-307.

87. Ralph AP, Read C, Johnston V, de Dassel JL, Bycroft K, Mitchell A, et al. Improving delivery of secondary prophylaxis for rheumatic heart disease in remote Indigenous communities: Study protocol for a stepped-wedge randomised trial. Trials 2016;17(1).

88. Rodriguez V, Giuffre C, Villa S, Almada G, Prasopa-Plaizier N, Gogna M, et al. A multimodal intervention to improve hand hygiene in ICUs in Buenos Aires, Argentina: A stepped wedge trial. Int J Qual Health Care 2015;27(5):405-411.

89. Shah L, Rojas M, Mori O, Zamudio C, Kaufman JS, Otero L, et al. Implementation of a stepped-wedge cluster randomized design in routine public health practice: design and application for a tuberculosis (TB) household contact study in a high burden area of Lima, Peru. BMC Public Health 2015;15:587.

90. Suman A, Schaafsma FG, Elders PJM, van Tulder MW, Anema JR. Cost-effectiveness of a multifaceted implementation strategy for the Dutch multidisciplinary guideline for nonspecific low back pain: design of a stepped-wedge cluster randomised controlled trial. BMC Public Health 2015;15:522.

91. Tielsch JM, Katz J, Zeger SL, Khatry SK, Shrestha L, Breysse P, et al. Designs of two randomized, community-based trials to assess the impact of alternative cookstove installation on respiratory illness among young children and reproductive outcomes in rural Nepal. BMC Public Health 2014;14:1271.

92. Tiono AB, Pinder M, N'Fale S, Faragher B, Smith T, Silkey M, et al. The AvecNet Trial to assess whether addition of pyriproxyfen, an insect juvenile hormone mimic, to long-lasting insecticidal mosquito nets provides additional protection against clinical malaria over current best practice in an area with pyrethroid-resistant vectors in rural Burkina Faso: Study protocol for a randomised controlled trial. Trials 2015;16(1).

93. Toftegaard BS, Bro F, Vedsted P. A geographical cluster randomised stepped wedge study of continuing medical education and cancer diagnosis in general practice. Implementation Science 2014;9:159.

94. van Daalen FV, Prins JM, Opmeer BC, Boermeester MA, Visser CE, van Hest RM, et al. A cluster randomized trial for the implementation of an antibiotic checklist based on validated quality indicators: The AB-checklist. BMC Infect Dis 2015;15(1).

95. Var C, Bazzano AN, Srivastav SK, Welty JC, Ek NI, Oberhelman RA. Newborn Infection Control and Care Initiative for health facilities to accelerate reduction of newborn mortality (NICCI): Study protocol for a randomized controlled trial. Trials 2015;16(1).

96. Verberne CJ, Zhan Z, Van Den Heuvel E, Grossmann I, Doornbos PM, Havenga K, et al. Intensified follow-up in colorectal cancer patients using frequent Carcino-Embryonic Antigen (CEA) measurements and CEA-triggered imaging: Results of the randomized "CEAwatch" trial. Eur J Surg Oncol 2015;41(9):1188-1196.

97. Wald HL, Bandle B, Richard AA, Min S, Capezuti E. A Trial of electronic surveillance feedback for quality improvement at Nurses Improving Care for Healthsystem Elders (NICHE) hospitals. Am J Infect Control 2014 Oct;42(10 Suppl):S250-6.

98. Williamson A, Redman S, Haynes A, Barker D, Jorm L, Green S, et al. Supporting Policy In health with Research: an Intervention Trial (SPIRIT)—protocol for a stepped wedge trial. BMJ Open 2014;4(7).

99. Wu M-, Wu C-, Chen B-. Behavioral Intervention and Decreased Daily Melamine Exposure from Melamine Tableware. Environ Sci Technol 2015;49(16):9964-9970.

100. Hoogendijk EO, van der Horst HE, van de Ven PM, Twisk JWR, Deeg DJH, Frijters DHM, et al. Effectiveness of a Geriatric Care Model for frail older adults in primary care: Results from a stepped wedge cluster randomised trial. Eur J Intern Med 2015;28(43).

101. Ononge S, Campbell OMR, Kaharuza F, Lewis JL, Fielding K, Mirembe F. Effectiveness and safety of misoprostol distributed to antenatal women to prevent postpartum haemorrhage after child-births: a stepped-wedge cluster-randomised trial. BMC Pregnancy and Childbirth 2015;15(315).
