# Supplementary material for: Unequal cluster sizes in stepped-wedge cluster randomised trials: a systematic review
Source: BMJ Open. 2017 Nov 15;7(11):e017151. doi: 10.1136/bmjopen-2017-017151 (PMC5695383; doi:10.1136/bmjopen-2017-017151)
Supplement: Author's manuscript [file bmjopen-2017-017151.draft_revisions.pdf]

BMJ Open is committed to open peer review. As part of this commitment we make the peer review history of every article we publish publicly available.

When an article is published we post the peer reviewers' comments and the authors' responses online. We also post the versions of the paper that were used during peer review. These are the versions that the peer review comments apply to.

The versions of the paper that follow are the versions that were submitted during the peer review process. They are not the versions of record or the final published versions. They should not be cited or distributed as the published version of this manuscript.

BMJ Open is an open access journal and the full, final, typeset and author-corrected version of record of the manuscript is available on our site with no access controls, subscription charges or pay-per-view fees (http://bmjopen.bmj.com).

If you have any questions on BMJ Open's open peer review process please email editorial.bmjopen@bmj.com

BMJ Open

Unequal cluster sizes in stepped-wedge cluster randomised trials: a systematic review

Journal:	*BMJ Open*
Manuscript ID	bmjopen-2017-017151
Article Type:	Research
Date Submitted by the Author:	05-Apr-2017
Complete List of Authors:	Kristunas, Caroline; University of Leicester, Department of Health Sciences<br>Morris, Tom; University of Leicester, Leicester Clinical Trials Unit<br>Gray, Laura; University of Leicester, Department of Health Sciences
**Primary Subject Heading**:	Research methods
Secondary Subject Heading:	Public health
Keywords:	Stepped-wedge trial, Systematic review, Cluster randomised trial

SCHOLARONE™
Manuscripts

For peer review only

Unequal cluster sizes in stepped-wedge cluster randomised trials: a systematic review

Caroline Kristunas[1*], Tom Morris[2], Laura J Gray[1]

Authors Affiliations:

[1]Department of Health Sciences, University of Leicester, Leicester, UK
[2]Leicester Clinical Trials Unit, University of Leicester, Leicester, UK

**Corresponding author:* Caroline Kristunas; cak21@le.ac.uk

Keywords: Stepped-wedge trial; systematic review; cluster randomised trial.

For peer review only

ABSTRACT

Objectives: To investigate the extent to which cluster sizes vary in stepped-wedge cluster randomised trials (SW-CRTs) and whether any variability is accounted for during the sample size calculation or analysis of these trials.

Setting: Any, not limited to healthcare settings.

Participants: Any taking part in a SW-CRT published up to March 2016.

Primary and secondary outcome measures: The primary outcome is the difference between the cluster sizes assumed during the sample size calculation and those observed during the trial. Secondary outcomes include any reported variability in cluster sizes and whether the methods of sample size calculation and analysis accounted for any variability in cluster sizes.

Results: Of the 101 included SW-CRTs, 48% indicated that the included clusters were known to vary in size, yet only 13% of these accounted for this during the calculation of the sample size. However, 45% of the trials did use a method of analysis appropriate for when clusters vary in size. Full trial reports were available for 53 trials. The coefficient of variation in cluster size (CV) was calculated for 23 of these: the median CV was 0.41 (interquartile range: 0.22-0.52). Actual cluster sizes could be compared to those assumed during the sample size calculation for 14 (26%) of the trial reports: the cluster sizes were between 29% and 480% of that which had been assumed.

Conclusions: Cluster sizes often vary in SW-CRTs and this variability is frequently at a level that would cause a significant loss of power in a parallel CRT. Reporting of SW-CRTs also remains sub-optimal. The effect of unequal cluster sizes on the statistical power of SW-CRTs needs to be explored further and methods appropriate to studies with unequal cluster sizes need to be employed.

STRENGTHS AND LIMITATIONS OF THIS STUDY:

- To our knowledge this is the first systematic review to assess how often and to what extent cluster sizes are unequal in stepped-wedge cluster randomised trials (SW-CRTs) and determine whether the current methodology being used accounts for any variability in cluster sizes.
- This review used pre-specified search and study selection strategies as well as using double data extraction.
- Some cluster sizes could only be estimated due to the quality of the reporting of some studies.

For peer review only

BACKGROUND

Cluster randomised trials (CRTs) are often used to evaluate healthcare interventions that are implemented at the cluster level, for example within hospitals, general practices or across geographical areas, where treatment contamination would be likely to occur if individual randomisation was used.[1] In conventional parallel CRTs, clusters are generally randomised to either a control or intervention group (usually with equal numbers in each group). After baseline measurements have been taken, those randomised to the intervention group are exposed to the intervention for the remainder of the trial, whereas those in the control group are not.

The stepped-wedge CRT (SW-CRT) is a relatively recent variant of the CRT. In SW-CRTs the clusters are randomised to start the intervention at different time points, known as steps, rather than being randomised to either a control or an intervention group. All of the clusters generally start the trial in a control group, then switch to the intervention when their time comes, until by the end of the trial all of the clusters are exposed to the intervention. Figure 1 gives a schematic representation of a SW-CRT. Although use of this trial design is still relatively rare, recent systematic reviews have shown there to be a dramatic rise in the number of SW-CRTs published within the last few years.[2] However, the methodological advances for SW-CRTs lag behind those for other designs of trial.

Due to the variability that occurs in the natural sizes of some clusters, the actual observed cluster sizes often vary within a CRT.[1] Clusters may also vary in size due to differences in the rates of loss to follow-up and incidence rates across clusters.[1] Increasing variability in cluster sizes has been shown to cause a loss of statistical power in parallel CRTs,[3] particularly if the coefficient of variation in cluster size (CV) (given as the standard deviation of the cluster size divided by the mean cluster size) is greater than 0.23.[4] This can lead to a trial that is under-powered to detect the effect of the intervention that is being evaluated. Various sample size methods have thus been developed for parallel CRTs which account for any imbalance in cluster sizes, to ensure that the trial will be sufficiently powered.[4-7] However, although the effect of unequal cluster sizes has been reported for parallel CRTs, it has only been reported for cross-sectional SW-CRTs with a continuous outcome and analysed using generalised estimating equations (GEEs).[8] It is expected that some loss of power will be observed for SW-CRTs of some designs [9] and therefore a similar adjustment to the sample size calculation is likely to be required.

It has been recommended that mixed-effects models (MMs) or GEEs should be used to analyse SW-CRTs.[10] In particular, linear MMs can be used if cluster sizes are approximately equal, but if cluster sizes vary then generalised linear MM (GLMM) or GEEs are preferred.[10] This is because both of these methods can account for the variability in cluster sizes.[10] It is therefore to be expected that these methods will be employed in SW-CRTs where the cluster sizes are likely to vary.

A recent systematic review of SW-CRTs found that few studies reported any variation in cluster sizes, however, major deficiencies were found in the reporting [11] and so this is likely to be an underestimation of the true extent of variability in cluster sizes. The size of the variability in cluster sizes in SW-CRTs has not previously been reported, nor how any

For peer review only

imbalance in cluster sizes is being adjusted for during the calculation of the sample size or the analysis of these trials. The primary aims of our review were therefore to:

- Determine how often and to what extent cluster sizes are unequal in SW-CRTs.
- Determine how unequal cluster sizes are being taken into account during the calculation of the sample size and the analysis of SW-CRTs.

METHODS

Search strategy

We identified a recent systematic review of SW-CRTs that included studies up to 23 October 2014.[11] We then updated this review by searching MEDLINE, Scopus and EBSCO Host, up to 21 March 2016, using an adapted version of the authors' search strategy. The search strategy is provided in Supplementary File 1. The studies that were included were from protocols or independent full reports of SW-CRTs in both healthcare and non-healthcare settings.[11] The studies had to be randomised trials, using cluster randomisation, and have at least two steps. Trials that were not published in English, were individually or non-randomised trials, trials with a cross-over design and those that were retrospectively analysed as a SW-CRT were excluded.[11] We focussed on original research studies and primary study reports.

Once duplicates had been removed, the titles and abstracts of the studies identified by the search were screened for eligibility. Full-text articles were then obtained for all potentially eligible studies and screened independently by two authors (CK and TM). Those studies found not to meet the eligibility criteria were excluded and the reasons for exclusion tabulated. Any differences of opinion were resolved by discussions with LG.

Data extraction

Data for all studies meeting the eligibility criteria were extracted by CK and checked by TM. Any differences of opinion were resolved through discussions with all authors. A data extraction form was developed, then tested and refined on a small number of studies.

Basic trial characteristics are reported. These include the year of publication, type of cluster, the type of primary outcome (binary, continuous, time-to-event etc.), number of clusters required and whether any restrictions related to cluster sizes were used in the randomisation procedure.

Information was also extracted in relation to variability in cluster sizes. We report whether any indication was given that clusters were known to vary in size prior to the trial commencing and whether any variability in cluster sizes was accounted for during the randomisation process, calculation of the sample size or analysis. We also recorded the number and sizes of the clusters assumed during the sample size calculation. For completed trials we also extracted the actual cluster sizes and reported the variability in cluster sizes that was observed during the trial and a comparison of the actual cluster sizes with the cluster sizes that were assumed during the sample size calculations.

Reporting of SW-CRTs is poor [11] and so to increase the number of trials available for a comparison of cluster sizes, some cluster sizes had to be estimated from whatever information was available. Cluster sizes per time period, at a specific time point (such as

at baseline) or the total cluster sizes over the full duration of the trial were preferred. If this information was not available then other information relating to the sizes of the clusters were used to estimate the cluster sizes, so that an estimate of the variability in the sizes of the clusters could be made. This included using the reported number of eligible individuals in each cluster, mean cluster size per randomisation group, range of cluster sizes etc. The cluster sizes and comparison of cluster sizes are reported separately for the actual cluster sizes and those that were estimated.

Analysis

We summarise the basic trial characteristics, methods of sample size calculation and analysis, and number and size of clusters assumed during the sample size calculation for study reports and protocols. Actual cluster sizes, estimated actual cluster sizes and comparisons of actual cluster sizes with those that were assumed during the calculation of the sample size are summarised for trial reports only.

RESULTS

We identified 371 records through our search of electronic databases. After combining this with the records identified by Martin et al,[11] removing three protocols for which the full reports had been identified, and after removing duplicates, we were left with 330 records. We screened these records and excluded 229 which did not meet our eligibility criteria (Figure 2). A full list of the 101 included studies is given in Supplementary File 2.

The general characteristics of the included trials are presented in Table 1. Full trial reports were available for 53 (52%) trials, with only protocols available for the remaining 48 (48%) trials. Two (2%) trials were conducted as pilot studies. A primary outcome was reported for 87 (86%) trials, of which the majority were proportions (41%), rates (16%) or continuous (16%) outcomes and six (7%) reported multiple primary outcomes. Three (3%) trials did not report a primary outcome and for the remaining 11 (11%) trials the primary outcome was not clear. For the majority of the trials (52%), the clusters were comprised of health care facilities, such as hospitals, hospital wards, clinics or GP practices. The remaining clusters comprised of geographical areas/communities (10%), residential/long-term care facilities (9%), health care professionals (9%), educational facilities (6%), rehabilitation clinics/programs (5%) family groups/households (4%), or other groups (5%).

Table 1: Trial characteristics of included SW-CRTs. Values are numbers (percentages).

	Number (%)
**Publication type**	
Report	53 (52.5)
Protocol	48 (47.5)
**Pilot study**	2 (2)
**Primary outcome reported**	**87 (86.1)**
Proportion	36 (41.4)
Rate Continuous	14 (16.1)
Count	14 (16.1)
Discrete	7 (8.0)

For peer review only

Multiple	6 (6.9)
Binary	6 (6.9)
Time-to-event	3 (3.4)
	1 (1.1)
**Types of cluster:**	
Health care facilities	53 (52.5)
Geographical areas/communities	10 (9.9)
Residential/long-term care facilities	9 (8.9)
Health care professionals (including groups thereof)	9 (8.9)
Education facilities	6 (5.9)
Rehabilitation clinics/programs	5 (5.0)
Family groups/households	4 (4.0)
Other	5 (5.0)
Indication of clusters being known to vary in size	48 (47.5)
Sample size calculation presented	82 (81.2)
**Accounted for unequal cluster sizes in sample size calculation**	**6 (5.9)**
Used coefficient of variation in cluster size	2 (33.3)
Accounted for in simulation	2 (33.3)
Unclear	2 (33.3)
**Reported number of clusters required**	**71 (70.3)**
Median (range) number of clusters required	11 (3 - 142)
**Reported required cluster size**	**57 (56.4)**
Median (range) per time period (n=23)	30 (4 - 565)
Median (range) overall (n=34)	51 (4 – 500,000)
Reported total sample size required[1]	64 (63.4)
**Method of analysis**	**94 (93)**
GEEs	13 (13)
MM	57 (56)
GLMM	32 (32)
LMM	7 (7)
Unclear	18 (18)
Other models	13 (13)
Generalised LM	5 (5)
GLM (accounting for clustering)	5 (5)
LM/ANCOVA	6 (6)
Simple analysis (McNemar's, Mann-Whitney U, t-test, ANOVA, bivariate, Wilcoxon signed-rank)	8 (8)
Unclear	5 (5)
Not given	2 (2)

[1] *Total sample size required either stated or enough detail given for it to be reproduced.*

Just under half (48%) of the trials gave some indication that the clusters were known to vary in size. Yet few of these accounted for inequality in cluster size during the

For peer review only

randomisation process (29%) or sample size calculation (13%). Nine stratified by cluster size, one pair-matched according to cluster size, two restricted randomisation by cluster size, one used minimisation to balance cluster sizes and one used block randomisation by cluster size. One additional trial used stratification, but did not state for which variables stratification was applied.

Sample size calculation

Only 82 (81%) of the included studies presented a sample size calculation, of which 71 reported the number of clusters required and 57 reported the required cluster sizes (23 cluster size per time period, 34 total cluster size). The median (range) number of clusters required for these trials was 11 (3 - 142) and the median (range) cluster size was 30 (4 - 565) for those that gave the cluster size per time period and 51 (4-500,000) for those that gave the total cluster size over the duration of the trial.

In total only six (6%) trials reported that they had accounted for unequal cluster sizes when calculating the required sample size. Two used an estimate of the coefficient of variation in cluster size, two used simulations to calculate the required sample size, varying the cluster sizes in their simulations, and for the remaining two trials the method used to account for unequal cluster sizes was unclear.

Methods of analysis

A small proportion of the trials (12%) attempted to adjust for any inequality in cluster size through the inclusion of covariates in the analysis and one trial planned to conduct an individual level analysis, rather than cluster level analysis, if clusters were found to vary in size.

The majority of analyses were conducted using multilevel models (MM) (56%), with 32 studies using generalised linear MMs, seven using linear MMs and 18 not distinguishing between the two. An equal number of trials used generalised estimating equations (13%) as used simple regression models (linear or generalised linear models) (13%). Only five of those that used simple regression models accounted for the clustering of the data during the analysis. The method of analysis for the primary outcome was unclear for five studies and two studies did not report in their protocol details of how the analysis would be conducted. The remaining 11 (11%) studies employed simple analysis methods, such as ANOVA and two sample t-tests, which did not account for the clustering of the data.

Reporting of actual cluster sizes

Of the 53 included full trial reports, it was only possible to determine the actual cluster sizes for 13 (25%) of the trials. Five of these trials gave the cluster sizes during each time period, four gave the size of each cluster at a specific time point, such as at baseline or the point of analysis and the remaining four reports gave the total size of each cluster over the duration of the whole trial. One further trial gave the number of eligible individuals in each cluster. A further eight (15%) reports gave a summary measure of the cluster sizes, such as the mean per month, per randomisation group, per intervention group etc. or the range of cluster sizes. Two (4%) reports gave the sizes of the randomisation groups only.

Comparison of cluster sizes

For peer review only

Few study reports presented both the required cluster sizes and the actual cluster sizes. The way in which cluster sizes were presented varied between studies and some only presented summary measures of the cluster sizes. Of the 24 trial reports that presented some indication of the cluster sizes, only 14 (58%) also stated the required cluster size. For those trials that presented the actual cluster sizes, the individual cluster sizes, either over the whole trial or at each time point, were between 29% and 480% of the required cluster size. For those trials that reported a summary measure of the actual cluster sizes, the cluster sizes were between 30% and 209% of the required cluster size. The average cluster size for each trial ranged between 66% and 214% of the required cluster size.

None of the trial reports presented the actual value of the coefficient of variation (CV) in cluster size. It was possible to estimate the CV in cluster size for 23 of the trials, of which only two experienced no variability in cluster size. The median CV in cluster size was 0.41 (interquartile range: 0.22-0.52) and the maximum value was 1.29. The majority of trials had a CV in cluster size >0.23 (70%), so would have experienced a significant loss of power had these been parallel CRTs. None of these trials reported that they accounted for unequal cluster sizes during the sample size calculation.

DISCUSSION

We conducted a systematic review to assess how often and to what extent cluster sizes are unequal in SW-CRTs and to determine how any inequality in cluster sizes is currently being taken into account during the calculation of the sample sizes and the analysis of these trials. Of the 101 SW-CRTs included, almost half (48%) gave an indication that the clusters were known to vary in size, yet only a small proportion of these accounted for this during the calculation of the sample size. About 45% of the trials did however employ a method of analysis deemed appropriate for when cluster sizes are unequal (GLMM or GEEs).[10] Methods of sample size calculation which account for any variability in cluster sizes exist for parallel CRTs,[4-7] however, no such methods have been developed for use with SW-CRTs. These methods may be able to be adapted for use with SW-CRTs.

Full trial reports were available for 53 trials. Only a quarter of these reported the sizes of each cluster, either per time period, at a specific point during the trial or the total size over the whole duration of the trial. The CV in cluster size was calculated based on this information. A further eight trials reported a summary measure of the cluster sizes, such as the mean cluster size per month or the mean cluster size per randomisation group. From these summary measures we were able to produce an estimate of the CV in cluster size for each of these trials. One further trial only gave the number of eligible individuals in each cluster and so, assuming equal recruitment rates in each cluster, it was also possible to estimate the CV in cluster size for this trial. Only two trials experienced no variability in cluster size. The majority (70%) of trials had a CV in cluster size >0.23 and so if these had been parallel CRTs we would have expected these trials to have suffered a significant loss of statistical power.[4] A recent simulation study has shown that for cross-sectional SW-CRTs with a continuous outcome, even high variability in cluster sizes does not cause a significant loss of power.[8] However, a recent systematic review found that

For peer review only

the majority of SW-CRTs are not of a cross-sectional design and it is for these trials that the effect of unequal cluster sizes is still unknown.[12]

The actual cluster sizes also varied considerably from those that were assumed during the calculation of the required sample size, with reported cluster sizes being between 29% and 480% of that which was required. This would suggest that many of the trials we examined are likely to have been considerably over- or under-powered. These comparisons could only be made for 14 (26%) of the trials with published reports. Information both on the sizes of the clusters that were assumed during the calculation of the sample size and the actual cluster sizes that were observed during the trial were often not reported.

The majority of systematic reviews of SW-CRTs have been conducted with the aim of assessing the quality and breadth of SW-CRTs.[2, 13, 14] A couple of recent systematic reviews have also been conducted to assess the quality of reporting and methodological rigor of SW-CRTs,[11] as well as the statistical methodology being used and available for these trials.[15] However, none of these systematic reviews have assessed the prevalence and severity of unequal cluster sizes in SW-CRTs, nor the statistical methodology being used to account for this variability in cluster sizes. Our review has highlighted the high degree of variability in cluster sizes that is observed in SW-CRTs. Knowing that cluster sizes often vary in SW-CRTs can allow researchers to plan for an expected inequality in cluster sizes, taking this into account when calculating the required sample size for these trials and choosing an appropriate method of analysis. We also highlight the need for improved quality of reporting of cluster sizes in SW-CRTs, which concurs with the findings of previous reviews [11, 12] and the need for wider implementation of methodologies which account for unequal cluster sizes. These findings are in line with what has been shown more generally in CRTs.[16]

Our study has several strengths. In order to minimise the potential for bias during the review, we pre-specified search and study selection strategies. We also did not limit our search to publications from journals with a high impact factor, increasing the generalisability of our findings. However, our study does have limitations as well. For example, selection bias may have been introduced by the choice to only include studies published in English. It was also difficult to ensure the identification of all SW-CRT, as many don't include in either their title or abstract the common terms that identify a SW-CRT. We included in our search strategy all of the common identifiers that have been included in previous reviews, yet this is still not sufficient to identify studies where the researchers weren't aware that their studies were SW-CRTs and therefore did not include common identifiers in their title or abstract. This may introduce some selection bias. The poor reporting of SW-CRTs [11] may limit the generalisability of the findings of our study. It was only possible to make a comparison of cluster sizes and to calculate the CV in cluster size for a small number of studies, and these are likely to be those studies that have a better quality of reporting than the other studies. The estimations that were made when determining the observed cluster sizes may also result in an under- or over-estimation of the degree of variability in cluster sizes and the comparability of the observed cluster sizes with those which were required.

For peer review only

Unequal cluster sizes is a common problem in SW-CRTs and this variability in cluster sizes is most often at a level that would cause a significant loss of power for parallel CRTs. Variability in cluster sizes is not being accounted for during the calculation of the sample size for these trials and the reporting of the actual cluster sizes observed during these trials is poor, which limits the scope of this review. Appropriate methods of sample size calculation which account for variability in cluster sizes should be developed by methodological statisticians and perhaps implemented in appropriate software in order to allow their widespread use. Further research is also required to investigate the effect of unequal cluster sizes on the statistical power or cohort SW-CRTs and those with a binary outcome. The development of reporting guidelines for SW-CRTs is needed to improve the sub-optimal quality of reporting for the sample size calculations and of the actual observed cluster sizes in SW-CRTs.

CONCLUSIONS

Cluster sizes were found to vary in SW-CRTs and frequently this was at a level that in a parallel CRT would result in a significant loss of statistical power. The effect of unequal cluster sizes on SW-CRTs is yet to be reported for several designs and so the effect of this observed variability is currently unknown. Methods of sample size calculation that are used for these trials rarely account for any variability in cluster size. Reporting of cluster sizes in both the reported sample size calculation and those actually observed during the trial is poor. Authors need to provide greater detail of how the required sample size has been calculated as well as providing details of the actual cluster sizes observed during the trial, preferably at each step of the trial. Researchers should also be aware that cluster sizes are likely to vary in SW-CRTs and so sample size calculation methods and analysis methods should be used which can account for this variability.

FUNDING

CK is funded by a National Institute for Health Research (NIHR) Research Methods Fellowship. The authors would also like to acknowledge support from the NIHR Collaboration for Leadership in Applied Health Research and Care – East Midlands (NIHR CLAHRC – EM), Leicester Clinical Trials Unit and the NIHR Leicester-Loughborough Diet, Lifestyle and Physical Activity Biomedical Research Unit, which is a partnership between University Hospitals of Leicester NHS Trust, Loughborough University and the University of Leicester. The views expressed are those of the authors and not necessarily those of the NIHR or the Department of Health.

COMPETING INTERESTS

None declared.

DATA SHARING

No additional data are available.

For peer review only

CONTRIBUTORS

LJG conceptualised the review. CK carried out the systematic review, designed the data extraction form, identified the studies for inclusion, extracted data, performed the data analysis and wrote the first draft. TM carried out the second data extraction and critically reviewed the contents of the paper. LJG resolved any differences between CK and TM in the data extraction. All authors contributed to the writing of the paper, read and approved the final manuscript.

REFERENCES

1 Eldridge S, Kerry S. A Practical Guide to Cluster Randomised Trials in Health Services Research. US: John Wiley & Sons Inc 2012.

2 Beard E, Lewis J, Copas A, et al. Stepped wedge randomised controlled trials: systematic review of studies published between 2010 and 2014, *Trials* 2015;16:1-14.

3 Lauer SA, Kleinman KP, Reich NG. The Effect of Cluster Size Variability on Statistical Power in Cluster-Randomized Trials, *PLoS ONE* 2015;10:e0119074.

4 Eldridge SM, Ashby D, Kerry S. Sample size for cluster randomized trials: effect of coefficient of variation of cluster size and analysis method, *Int J Epidemiol* 2006;35:1292-300.

5 Kerry SM, Bland JM. Unequal cluster sizes for trials in English and Welsh general practice: implications for sample size calculations, *Stat Med* 2001;20:377-90 doi:AID-SIM799>3.0.CO;2-N.

6 Manatunga AK, Hudgens MG, Chen S. Sample Size Estimation in Cluster Randomized Studies with Varying Cluster Size, *Biometrical Journal* 2001;43:75-86.

7 Pan W. Sample Size and Power Calculations with Correlated Binary Data, *Control Clin Trials* 2001;22:211-27.

8 Kristunas CA, Smith KL, Gray LJ. An imbalance in cluster sizes does not lead to notable loss of power in cross-sectional, stepped-wedge cluster randomised trials with a continuous outcome, *Trials* 2017;18:109.

9 Baio G, Copas A, Ambler G, et al. Sample size calculation for a stepped wedge trial, *Trials* 2015;16:354.

10 Hussey MA, Hughes JP. Design and analysis of stepped wedge cluster randomized trials, *Contemporary Clinical Trials* 2007;28:182-91.

11 Martin J, Taljaard M, Girling A, et al. Systematic review finds major deficiencies in sample size methodology and reporting for stepped-wedge cluster randomised trials, *BMJ open* 2016;6:e010166 doi:10.1136/bmjopen-2015-010166.

For peer review only

12 Grayling MJ, Wason JM, Mander AP. Stepped wedge cluster randomized controlled trial designs: a review of reporting quality and design features, *Trials* 2017;18:33.

13 Brown C, Lilford R. The stepped wedge trial design: a systematic review, *BMC Medical Research Methodology* 2006;6:54.

14 Mdege ND, Man M, Taylor (nee Brown), Celia A, et al. Systematic review of stepped wedge cluster randomized trials shows that design is particularly used to evaluate interventions during routine implementation, *J Clin Epidemiol* 2011;64:936-48.

15 Barker D, McElduff P, D'Este C, et al. Stepped wedge cluster randomised trials: A review of the statistical methodology used and available, *BMC Medical Research Methodology* 2016;16 doi:10.1186/s12874-016-0176-5.

16 Rutterford C, Taljaard M, Dixon S, et al. Reporting and methodological quality of sample size calculations in cluster randomized trials could be improved: a review, *J Clin Epidemiol* 2015;68:716-23.

FIGURE LEGENDS

Figure 1: Schematic representation of a stepped-wedge cluster randomised trial with four steps. The white areas represent control periods and the shaded areas represent intervention periods.

Figure 2: Flow chart showing the studies identified by the systematic review.

For peer review only

Figure 1: Schematic representation of a stepped-wedge cluster randomised trial with four steps. The white areas represent control periods and the shaded areas represent intervention periods.

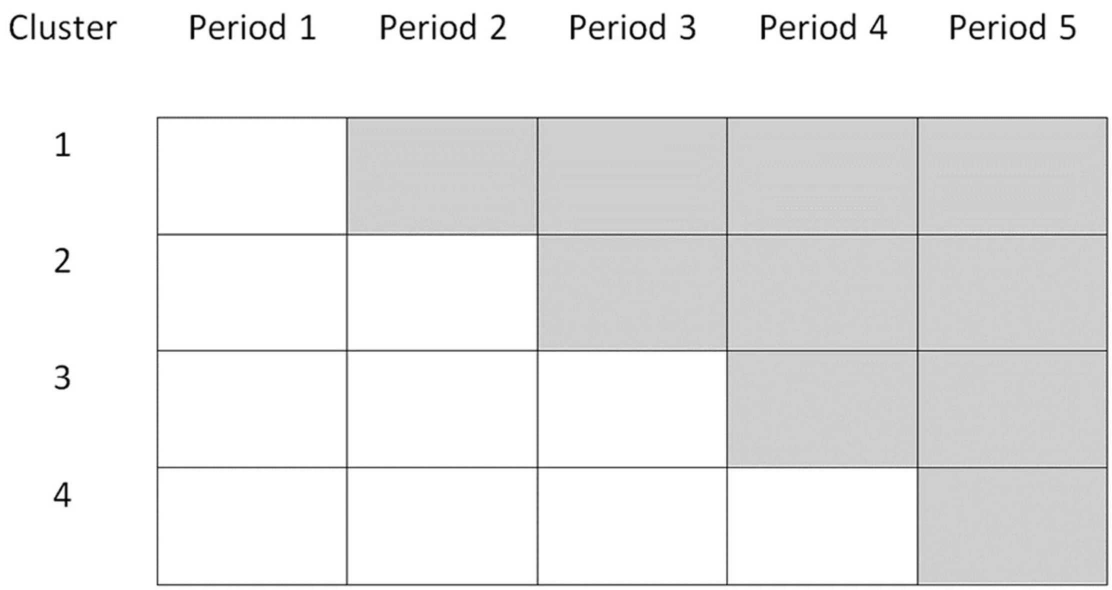

review only

Figure 2: Flow chart showing the studies identified by the systematic review.

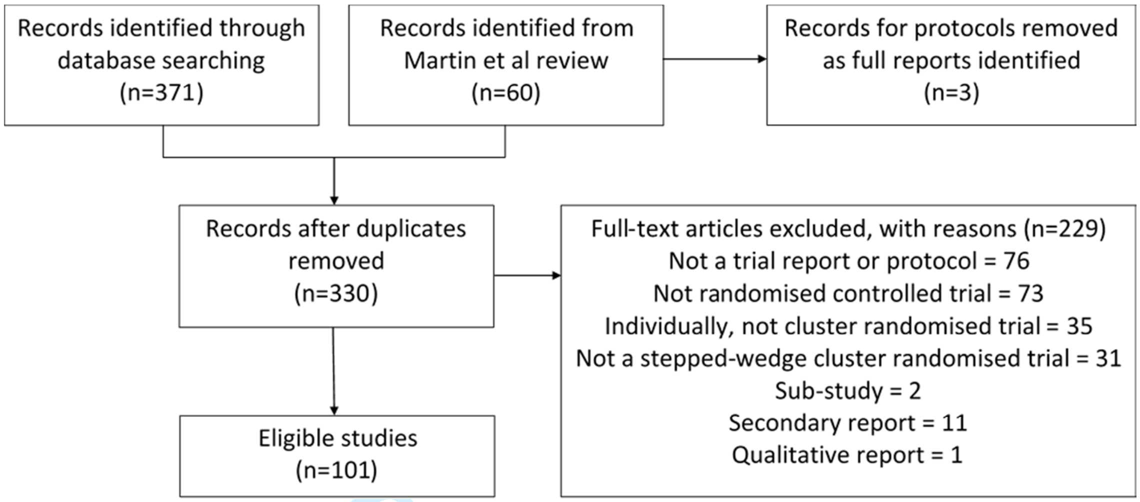

For peer review only - http://bmjopen.bmj.com/site/about/guidelines.xhtml

SYSTEMATIC REVIEW SEARCH STRATEGY

1. step$ wedge.ti,ab.
2. experimentally staged introduction.ti,ab.
3. delayed intervention.ti,ab.
4. (one directional cross over design or one directional crossover design).ti,ab.
5. ((incremental or phased or staggered or stepwise or step wise or delayed) adj1 (recruitment or introduction or implementation)).ti,ab.
6. or/1-5
7. limit 6 to english language
8. limit 7 to randomized controlled trial
9. limit 8 to humans
10. limit 9 to yr="2014-current"

For peer review only

1. Craine N, Whitaker R, Perrett S, Zou L, Hickman M, Lyons M. A stepped wedge cluster randomized control trial of dried blood spot testing to improve the uptake of hepatitis C antibody testing within UK prisons. European journal of public health. 2014.

2. Palmay L, Elligsen M, Walker SAN, Pinto R, Walker S, Einarson T, et al. Hospital-wide rollout of antimicrobial stewardship: A stepped-wedge randomized trial. Clinical Infectious Diseases. 2014;59(6):867-74.

3. van de Steeg L, R IJ, Langelaan M, Wagner C. Can an e-learning course improve nursing care for older people at risk of delirium: a stepped wedge cluster randomised trial. BMC geriatrics. 2014;14:69.

4. Haugen AS, Softeland E, Almeland SK, Sevdalis N, Vonen B, Eide GE, et al. Effect of the World Health Organization Checklist on Patient Outcomes: A Stepped Wedge Cluster Randomized Controlled Trial. Annals of surgery. 2014.

5. Stern A, Mitsakakis N, Paulden M, Alibhai S, Wong J, Tomlinson G, et al. Pressure ulcer multidisciplinary teams via telemedicine: a pragmatic cluster randomized stepped wedge trial in long term care. BMC Health Serv Res. 2014;14:83.

6. Enns E, Rhemtulla R, Ewa V, Fruetel K, Holroyd-Leduc JM. A controlled quality improvement trial to reduce the use of physical restraints in older hospitalized adults. Journal of the American Geriatrics Society. 2014;62(3):541-5.

7. Roy A, Anaraki S, Hardelid P, Catchpole M, Rodrigues LC, Lipman M, et al. Universal HIV testing in London tuberculosis clinics: A cluster randomised controlled trial. European Respiratory Journal. 2013;41(3):627-34.

8. Mhurchu CN, Gorton D, Turley M, Jiang Y, Michie J, Maddison R, et al. Effects of a free school breakfast programme on children's attendance, academic achievement and short-term hunger: Results from a stepped-wedge, cluster randomised controlled trial. Journal of Epidemiology and Community Health. 2013;67(3):257-64.

9. Leontjevas R, Gerritsen DL, Smalbrugge M, Teerenstra S, Vernooij-Dassen MJFJ, Koopmans RTCM. A structural multidisciplinary approach to depression management in nursing-home residents: A multicentre, stepped-wedge cluster-randomised trial. The Lancet. 2013;381(9885):2255-64.

10. Gruber JS, Reygadas F, Arnold BF, Ray I, Nelson K, Colford Jr JM. A stepped wedge, cluster-randomized trial of a household UV-disinfection and safe storage drinking water intervention in rural Baja California Sur, Mexico. American Journal of Tropical Medicine and Hygiene. 2013;89(2):238-45.

11. Durovni B, Saraceni V, Moulton LH, Pacheco AG, Cavalcante SC, King BS, et al. Effect of improved tuberculosis screening and isoniazid preventive therapy on incidence of tuberculosis and death in patients with HIV in clinics in Rio de Janeiro, Brazil: A stepped wedge, cluster-randomised trial. The Lancet Infectious Diseases. 2013;13(10):852-8.

12. Bashour HN, Kanaan M, Kharouf MH, Abdulsalam AA, Tabbaa MA, Cheikha SA. The effect of training doctors in communication skills on women's satisfaction with doctor-woman relationship during labour and delivery: A stepped wedge cluster randomised trial in Damascus. BMJ Open. 2013;3(8).

13. van den Broek IV, van Bergen JE, Brouwers EE, Fennema JS, Gotz HM, Hoebe CJ, et al. Effectiveness of yearly, register based screening for chlamydia in the Netherlands: Controlled trial with randomised stepped wedge implementation. BMJ: British Medical Journal. 2012;345(7869):1-12.

14. Monse B, Duijster D, Sheiham A, Grijalva-Eternod CS, van Palenstein Helderman W, Hobdell MH. The effects of extraction of pulpally involved primary teeth on weight, height and BMI in underweight Filipino children. A cluster randomized clinical trial. BMC public health. 2012;12:725.

15. Horner C, Wilcox M, Barr B, Hall D, Hodgson G, Parnell P, et al. The longitudinal prevalence of MRSA in care home residents and the effectiveness of improving infection prevention knowledge and practice on colonisation using a stepped wedge study design. BMJ Open. 2012;2(1):e000423.

16. Fuller C, Michie S, Savage J, McAteer J, Besser S, Charlett A, et al. The Feedback Intervention Trial (FIT)--improving hand-hygiene compliance in UK healthcare workers: a stepped wedge cluster randomised controlled trial. PloS one. 2012;7(10):e41617.

17. Chinbuah MA, Kager PA, Abbey M, Gyapong M, Awini E, Nonvignon J, et al. Impact of community management of fever (using antimalarials with or without antibiotics) on childhood mortality: A cluster-

For peer review only

randomized controlled trial in Ghana. American Journal of Tropical Medicine and Hygiene. 2012;87(SUPPL.5):11-20.

18. Etchells E, Adhikari NK, Wu R, Cheung M, Quan S, Mraz R, et al. Real-time automated paging and decision support for critical laboratory abnormalities. BMJ quality & safety. 2011;20(11):924-30.

19. Bacchieri G, Barros AJ, Santos JV, Goncalves H, Gigante DP. A community intervention to prevent traffic accidents among bicycle commuters. Revista de saude publica. 2010;44(5):867-75.

20. Solomon E, Rees T, Ukoumunne OC, Metcalf B, Hillsdon M. The Devon Active Villages Evaluation (DAVE) trial of a community-level physical activity intervention in rural south-west England: A stepped wedge cluster randomised controlled trial. The International Journal of Behavioral Nutrition and Physical Activity. 2014;11:94.

21. Gozalo P, Prakash S, Qato DM, Sloane PD, Mor V. Effect of the bathing without a battle training intervention on bathing-associated physical and verbal outcomes in nursing home residents with dementia: A randomized crossover diffusion study. Journal of the American Geriatrics Society. 2014;62(5):797-804.

22. Foster JM, Hoskins G, Smith B, Lee AJ, Price D, Pinnock H. Practice development plans to improve the primary care management of acute asthma: randomised controlled trial. BMC Fam Pract. 2007;8:23.

23. Flannery DJ, Vazsonyi AT, Liau AK, Guo S, Powell KE, Atha H, et al. Initial behavior outcomes for the peacebuilders universal school-based violence prevention program. Developmental psychology. 2003;39(2):292-308.

24. Priestley G, Watson W, Rashidian A, Mozley C, Russell D, Wilson J, et al. Introducing Critical Care Outreach: A ward-randomised trial of phased introduction in a general hospital. Intensive Care Medicine. 2004;30(7):1398-404.

25. Husaini BA, Reece MC, Emerson JS, Scales S, Hull PC, Levine RS. A church-based program on prostate cancer screening for African American men: reducing health disparities. Ethn Dis. 2008;18(2 Suppl 2):S2-179-84.

26. Viera AJ, Garrett JM. Preliminary study of a school-based program to improve hypertension awareness in the community. Family medicine. 2008;40(4):264-70.

27. Barton A, Basham M, Foy C, Buckingham K, Somervill M. The Watcombe Housing Study: the short term effect of improving housing conditions on the health of residents. J Epidemiol Community Health. 2007;61(9):771-7.

28. Bailet LL, Repper KK, Piasta SB, Murphy SP. Emergent literacy intervention for prekindergarteners at risk for reading failure. Journal of Learning Disabilities. 2009;42(4):336-55.

29. Weiner M, El Hoyek G, Wang L, Dexter PR, Zerr AD, Perkins AJ, et al. A web-based generalist-specialist system to improve scheduling of outpatient specialty consultations in an academic center. Journal of general internal medicine. 2009;24(6):710-5.

30. Brown BB, Young J, Smith DP, Kneebone AB, Brooks AJ, Xhilaga M, et al. Clinician-led improvement in cancer care (CLICC)--testing a multifaceted implementation strategy to increase evidence-based prostate cancer care: phased randomised controlled trial--study protocol. Implementation science : IS. 2014;9:64.

31. Reuther S, Holle D, Buscher I, Dortmann O, Muller R, Bartholomeyczik S, et al. Effect evaluation of two types of dementia-specific case conferences in German nursing homes (FallDem) using a stepped-wedge design: Study protocol for a randomized controlled trial. Trials. 2014;15(1).

32. Bernabe-Ortiz A, Diez-Canseco F, Gilman RH, Cardenas MK, Sacksteder KA, Miranda JJ. Launching a salt substitute to reduce blood pressure at the population level: A cluster randomized stepped wedge trial in Peru. Trials. 2014;15(1).

33. Brimblecombe J, Ferguson M, Liberato SC, Ball K, Moodie ML, Magnus A, et al. Stores Healthy Options Project in Remote Indigenous Communities (SHOP@RIC): a protocol of a randomised trial promoting healthy food and beverage purchases through price discounts and in-store nutrition education. BMC Public Health. 2013;13:744.

34. Feng R, Li K, Cheng J, Xie S, Chai J, Wei P, et al. Toward integrated and sustainable prevention against diabetes in rural China: Study rationale and protocol of eCROPS. BMC Endocrine Disorders. 2013;13(28).

For peer review only

35. Keriel-Gascou M, Buchet-Poyau K, Duclos A, Rabilloud M, Figon S, Dubois JP, et al. Evaluation of an interactive program for preventing adverse drug events in primary care: study protocol of the InPAct cluster randomised stepped wedge trial. Implementation science : IS. 2013;8:69.

36. Praveen D, Patel A, McMahon S, Prabhakaran D, Clifford GD, Maulik PK, et al. A multifaceted strategy using mobile technology to assist rural primary healthcare doctors and frontline health workers in cardiovascular disease risk management: protocol for the SMARTHealth India cluster randomised controlled trial. Implement Sci. 2013;8:137.

37. Ward J, McGregor S, Guy RJ, Rumbold AR, Garton L, Silver BJ, et al. STI in remote communities: improved and enhanced primary health care (STRIVE) study protocol: a cluster randomised controlled trial comparing 'usual practice' STI care to enhanced care in remote primary health care services in Australia. BMC Infect Dis. 2013;13:425.

38. Bennett PN, Daly RM, Fraser SF, Haines T, Barnard R, Ockerby C, et al. The impact of an exercise physiologist coordinated resistance exercise program on the physical function of people receiving hemodialysis: a stepped wedge randomised control study. BMC Nephrol. 2013;14:204.

39. Poldervaart JM, Reitsma JB, Koffijberg H, Backus BE, Six AJ, Doevendans PA, et al. The impact of the HEART risk score in the early assessment of patients with acute chest pain: design of a stepped wedge, cluster randomised trial. BMC Cardiovasc Disord. 2013;13:77.

40. Rasmussen CD, Holtermann A, Mortensen OS, Sogaard K, Jorgensen MB. Prevention of low back pain and its consequences among nurses' aides in elderly care: a stepped-wedge multi-faceted cluster-randomized controlled trial. BMC Public Health. 2013;13:1088.

41. van Holland BJ, de Boer MR, Brouwer S, Soer R, Reneman MF. Sustained employability of workers in a production environment: design of a stepped wedge trial to evaluate effectiveness and cost-benefit of the POSE program. BMC Public Health. 2012;12:1003.

42. Dreischulte T, Grant A, Donnan P, McCowan C, Davey P, Petrie D, et al. A cluster randomised stepped wedge trial to evaluate the effectiveness of a multifaceted information technology-based intervention in reducing high-risk prescribing of non-steroidal anti-inflammatory drugs and antiplatelets in primary medical care: the DQIP study protocol. Implementation science : IS. 2012;7:24.

43. Flocke SA, Antognoli E, Step MM, Marsh S, Parran T, Mason MJ. A Teachable Moment Communication Process for smoking cessation talk: description of a group randomized clinician-focused intervention. BMC health services research. 2012;12:109.

44. Marshall T, Caley M, Hemming K, Gill P, Gale N, Jolly K. Mixed methods evaluation of targeted case finding for cardiovascular disease prevention using a stepped wedged cluster RCT. BMC public health. 2012;12:908.

45. Mouchoux C, Rippert P, Duclos A, Fassier T, Bonnefoy M, Comte B, et al. Impact of a multifaceted program to prevent postoperative delirium in the elderly: the CONFUCIUS stepped wedge protocol. BMC geriatrics. 2011;11:25.

46. Turner J, Kelly B, Clarke D, Yates P, Aranda S, Jolley D, et al. A randomised trial of a psychosocial intervention for cancer patients integrated into routine care: the PROMPT study (promoting optimal outcomes in mood through tailored psychosocial therapies). BMC Cancer. 2011;11:48.

47. Liddy C, Hogg W, Russell G, Wells G, Armstrong CD, Akbari A, et al. Improved delivery of cardiovascular care (IDOCC) through outreach facilitation: study protocol and implementation details of a cluster randomized controlled trial in primary care. Implement Sci. 2011;6:110.

48. The Gambia Hepatitis Intervention Study. The Gambia Hepatitis Study Group. Cancer Research. 1987;47(21):5782-7.

49. Schultz TJ, Kitson AL, Soenen S, Long L, Shanks A, Wiechula R, et al. Does a multidisciplinary nutritional intervention prevent nutritional decline in hospital patients? A stepped wedge randomised cluster trial. e-SPEN Journal. 2014;9(2):e84-e90.

50. Kjeken I, Berdal G, Bo I, Dager T, Dingsor A, Hagfors J, et al. Evaluation of a structured goal planning and tailored follow-up programme in rehabilitation for patients with rheumatic diseases: protocol for a pragmatic, stepped-wedge cluster randomized trial. BMC musculoskeletal disorders. 2014;15:153.

For peer review only

51. Stringer JS, Chisembele-Taylor A, Chibwesha CJ, Chi HF, Ayles H, Manda H, et al. Protocol-driven primary care and community linkages to improve population health in rural Zambia: the Better Health Outcomes through Mentoring and Assessment (BHOMA) project. BMC Health Serv Res. 2013;13 Suppl 2:S7.

52. De Allegri M, Pokhrel S, Becher H, Dong H, Mansmann U, Kouyate B, et al. Step-wedge cluster-randomised community-based trials: an application to the study of the impact of community health insurance. Health research policy and systems / BioMed Central. 2008;6:10.

53. Gucciardi E, Fortugno M, Horodezny S, Lou W, Sidani S, Espin S, et al. Will Mobile Diabetes Education Teams (MDETs) in primary care improve patient care processes and health outcomes? Study protocol for a randomized controlled trial. Trials. 2012;13:165.

54. Williams AL, Phillips CJ, Watkins A, Rushton AB. The effect of work-based mentoring on patient outcome in musculoskeletal physiotherapy: study protocol for a randomised controlled trial. Trials. 2014;15:409.

55. Mosha F, Winani S, Wood S, Changalucha J, Ngasalla B. Evaluation of the effectiveness of a clean delivery kit intervention in preventing cord infection and puerperal sepsis among neonates and their mothers in rural Mwanza Region, Tanzania. Tanzania health research bulletin. 2005;7(3):185-8.

56. Strijbos MJ, Steunenberg B, van der Mast RC, Inouye SK, Schuurmans MJ. Design and methods of the Hospital Elder Life Program (HELP), a multicomponent targeted intervention to prevent delirium in hospitalized older patients: efficacy and cost-effectiveness in Dutch health care. BMC Geriatr. 2013;13:78.

57. Zwijsen SA, Smalbrugge M, Eefsting JA, Twisk JWR, Gerritsen DL, Pot AM, et al. Coming to grips with challenging behavior: A cluster randomized controlled trial on the effects of a multidisciplinary care program for challenging behavior in Dementia. Journal of the American Medical Directors Association. 2014;15(7):531.e1-.e10.

58. Baker C, Huxley P, Dennis M, Islam S, Russell I. Alleviating staff stress in care homes for people with dementia: Protocol for stepped-wedge cluster randomised trial to evaluate a web-based mindfulness- stress reduction course. BMC Psychiatry 2015 12;15.

59. Baker M, Biringen Z, Meyer-Parsons B, Schneider A. Emotional attachment and emotional availability tele-intervention for adoptive families. Infant Ment Health J 2015;36(2):179-192.

60. Banga FR, Truijens SEM, Fransen AF, Dieleman JP, Van Runnard Heimel PJ, Oei GS. The impact of transmural multiprofessional simulation-based obstetric team training on perinatal outcome and quality of care in the Netherlands. BMC Med Educ 2014;14(1).

61. Brady MC, Stott D, Weir CJ, Chalmers C, Sweeney P, Donaldson C, et al. Clinical and cost effectiveness of enhanced oral healthcare in stroke care settings (SOCLE II): A pilot, stepped wedge, cluster randomized, controlled trial protocol. Int J Stroke 2015;10(6):979-984.

62. Britton B, McCarter K, Baker A, Wolfenden L, Wratten C, Bauer J, et al. Eating As Treatment (EAT) study protocol: A stepped-wedge, randomised controlled trial of a health behaviour change intervention provided by dietitians to improve nutrition in patients with head and neck cancer undergoing radiotherapy. BMJ Open 2015;5(7).

63. Chavane L, Merialdi M, Betrán AP, Requejo-Harris J, Bergel E, Aleman A, et al. Implementation of evidence-based antenatal care in Mozambique: A cluster randomized controlled trial: Study protocol. BMC Health Serv Res 2014;14(1).

64. Cowan JF, Micek M, Cowan JFG, Napua M, Hoek R, Gimbel S, et al. Early ART initiation among HIV-positive pregnant women in central Mozambique: a stepped wedge randomized controlled trial of an optimized Option B+ approach. Implementation Science 2015;10:61.

65. Dryden-Peterson S, Bennett K, Hughes MD, Veres A, John O, Pradhananga R, et al. An augmented SMS intervention to improve access to antenatal CD4 testing and ART initiation in HIV-infected pregnant women: A cluster randomized trial. PLoS ONE 2015;10(2).

66. Durovni B, Saraceni V, van den Hof S, Trajman A, Cordeiro-Santos M, Cavalcante S, et al. Impact of Replacing Smear Microscopy with Xpert MTB/RIF for Diagnosing Tuberculosis in Brazil: A Stepped-Wedge Cluster-Randomized Trial. PLoS Med 2014;11(12).

For peer review only

67. Fiscella K, Ogedegbe G, He H, Carroll J, Cassells A, Sanders M, et al. Blood Pressure Visit Intensification Study in Treatment: Trial design. Am Heart J 2015;170(6):1202-1210.

68. Golden MR, Kerani RP, Stenger M, Hughes JP, Aubin M, Malinski C, et al. Uptake and Population-Level Impact of Expedited Partner Therapy (EPT) on Chlamydia trachomatis and Neisseria gonorrhoeae: The Washington State Community-Level Randomized Trial of EPT. PLoS Med 2015;12(1):1-22.

69. Grace AM, Lippert S, Collins K, Pineda N, Tolani A, Walker R, et al. Educating health care professionals on human trafficking. Pediatr Emerg Care 2014;30(12):856-861.

70. Haines TP, O'Brien L, Mitchell D, Bowles K-, Haas R, Markham D, et al. Study protocol for two randomized controlled trials examining the effectiveness and safety of current weekend allied health services and a new stakeholder-driven model for acute medical/surgical patients versus no weekend allied health services. Trials 2015;16(1).

71. Happ MB, Sereika SM, Houze MP, Seaman JB, Tate JA, Nilsen ML, et al. Quality of care and resource use among mechanically ventilated patients before and after an intervention to assist nurse-nonvocal patient communication. Heart Lung J Acute Crit Care 2015;44(5):408-415.e2.

72. Hayden MK, Lin MY, Lolans K, Weiner S, Blom D, Moore NM, et al. Prevention of colonization and infection by klebsiella pneumoniae carbapenemase-producing enterobacteriaceae in long-term acute-care hospitals. Clin Infect Dis 2015;60(8):1154-1161.

73. Helitzer DL, Hathorn G, Benally J, Ortega C. Culturally relevant model program to prevent and reduce agricultural injuries. J Agric Saf Health 2014;20(3):175-198.

74. Hill A-, McPhail SM, Waldron N, Etherton-Beer C, Ingram K, Flicker L, et al. Fall rates in hospital rehabilitation units after individualised patient and staff education programmes: A pragmatic, stepped-wedge, cluster-randomised controlled trial. Lancet 2015;385(9987):2592-2599.

75. Hunter SB, Ober AJ, Paddock SM, Hunt PE, Levan D. Continuous quality improvement (CQI) in addiction treatment settings: design and intervention protocol of a group randomized pilot study. Addict Sci Clin Pract 2014;9:4.

76. Jarvik JG, Comstock BA, James KT, Avins AL, Bresnahan BW, Deyo RA, et al. Lumbar Imaging With Reporting Of Epidemiology (LIRE)-Protocol for a pragmatic cluster randomized trial. Contemp Clin Trials 2015;45:157-163.

77. Jordan S, Gabe-Walters ME, Watkins A, Humphreys I, Newson L, Snelgrove S, et al. Nurse-led medicines' monitoring for patients with dementia in care homes: A pragmatic cohort stepped wedge cluster randomised trial. PLoS ONE 2015;10(10).

78. Kelly PJ, Baker AL, Deane FP, Callister R, Collins CE, Oldmeadow C, et al. Study protocol: a stepped wedge cluster randomised controlled trial of a healthy lifestyle intervention for people attending residential substance abuse treatment. BMC Public Health 2015;15:465.

79. Li S, Wu Y, Du X, Li X, Patel A, Peterson ED, et al. Rational and design of a stepped-wedge cluster randomized trial evaluating quality improvement initiative for reducing cardiovascular events among patients with acute coronary syndromes in resource-constrained hospitals in China. Am Heart J 2015;169(3):349-355.

80. Marrin K, Wood F, Firth J, Kinsey K, Edwards A, Brain KE, et al. Option Grids to facilitate shared decision making for patients with Osteoarthritis of the knee: Protocol for a single site, efficacy trial. BMC Health Serv Res 2014;14(1).

81. Morrison LJ, Brooks SC, Dainty KN, Dorian P, Needham DM, Ferguson ND, et al. Improving use of targeted temperature management after out-of-hospital cardiac arrest: A stepped wedge cluster randomized controlled trial. Crit Care Med 2015;43(5):954-964.

82. Noel PH, Romero RL, Robertson M, Parchman ML. Key activities used by community based primary care practices to improve the quality of diabetes care in response to practice facilitation. Quality in Primary Care 2014;22(4):211-219.

83. Nosyk B, Krebs E, Min JE, Ahamad K, Buxton J, Goldsmith C, et al. The 'Expanded HIV care in opioid substitution treatment' (EHOST) cluster-randomized, stepped-wedge trial: A study protocol. Contemp Clin Trials 2015;45:201-209.

For peer review only

84. Olds DL, Baca P, McClatchey M, Ingoldsby EM, Luckey DW, Knudtson MD, et al. Cluster randomized controlled trial of intervention to increase participant retention and completed home visits in the Nurse-Family Partnership. Prevention Science 2015 08;16(6):778-788.

85. Palmer VJ, Chondros P, Piper D, Callander R, Weavell W, Godbee K, et al. The CORE study protocol: A stepped wedge cluster randomised controlled trial to test a co-design technique to optimise psychosocial recovery outcomes for people affected by mental illness in the community mental health setting. BMJ Open 2015;5(3).

86. Pickering BW, Dong Y, Ahmed A, Giri J, Kilickaya O, Gupta A, et al. The implementation of clinician designed, human-centered electronic medical record viewer in the intensive care unit: A pilot step-wedge cluster randomized trial. Int J Med Informatics 2016;84(5):299-307.

87. Ralph AP, Read C, Johnston V, de Dassel JL, Bycroft K, Mitchell A, et al. Improving delivery of secondary prophylaxis for rheumatic heart disease in remote Indigenous communities: Study protocol for a stepped-wedge randomised trial. Trials 2016;17(1).

88. Rodriguez V, Giuffre C, Villa S, Almada G, Prasopa-Plaizier N, Gogna M, et al. A multimodal intervention to improve hand hygiene in ICUs in Buenos Aires, Argentina: A stepped wedge trial. Int J Qual Health Care 2015;27(5):405-411.

89. Shah L, Rojas M, Mori O, Zamudio C, Kaufman JS, Otero L, et al. Implementation of a stepped-wedge cluster randomized design in routine public health practice: design and application for a tuberculosis (TB) household contact study in a high burden area of Lima, Peru. BMC Public Health 2015;15:587.

90. Suman A, Schaafsma FG, Elders PJM, van Tulder MW, Anema JR. Cost-effectiveness of a multifaceted implementation strategy for the Dutch multidisciplinary guideline for nonspecific low back pain: design of a stepped-wedge cluster randomised controlled trial. BMC Public Health 2015;15:522.

91. Tielsch JM, Katz J, Zeger SL, Khatry SK, Shrestha L, Breysse P, et al. Designs of two randomized, community-based trials to assess the impact of alternative cookstove installation on respiratory illness among young children and reproductive outcomes in rural Nepal. BMC Public Health 2014;14:1271.

92. Tiono AB, Pinder M, N'Fale S, Faragher B, Smith T, Silkey M, et al. The AvecNet Trial to assess whether addition of pyriproxyfen, an insect juvenile hormone mimic, to long-lasting insecticidal mosquito nets provides additional protection against clinical malaria over current best practice in an area with pyrethroid-resistant vectors in rural Burkina Faso: Study protocol for a randomised controlled trial. Trials 2015;16(1).

93. Toftegaard BS, Bro F, Vedsted P. A geographical cluster randomised stepped wedge study of continuing medical education and cancer diagnosis in general practice. Implementation Science 2014;9:159.

94. van Daalen FV, Prins JM, Opmeer BC, Boermeester MA, Visser CE, van Hest RM, et al. A cluster randomized trial for the implementation of an antibiotic checklist based on validated quality indicators: The AB-checklist. BMC Infect Dis 2015;15(1).

95. Var C, Bazzano AN, Srivastav SK, Welty JC, Ek NI, Oberhelman RA. Newborn Infection Control and Care Initiative for health facilities to accelerate reduction of newborn mortality (NICCI): Study protocol for a randomized controlled trial. Trials 2015;16(1).

96. Verberne CJ, Zhan Z, Van Den Heuvel E, Grossmann I, Doornbos PM, Havenga K, et al. Intensified follow-up in colorectal cancer patients using frequent Carcino-Embryonic Antigen (CEA) measurements and CEA-triggered imaging: Results of the randomized "CEAwatch" trial. Eur J Surg Oncol 2015;41(9):1188-1196.

97. Wald HL, Bandle B, Richard AA, Min S, Capezuti E. A Trial of electronic surveillance feedback for quality improvement at Nurses Improving Care for Healthsystem Elders (NICHE) hospitals. Am J Infect Control 2014 Oct;42(10 Suppl):S250-6.

98. Williamson A, Redman S, Haynes A, Barker D, Jorm L, Green S, et al. Supporting Policy In health with Research: an Intervention Trial (SPIRIT)—protocol for a stepped wedge trial. BMJ Open 2014;4(7).

99. Wu M-, Wu C-, Chen B-. Behavioral Intervention and Decreased Daily Melamine Exposure from Melamine Tableware. Environ Sci Technol 2015;49(16):9964-9970.

For peer review only

100. Hoogendijk EO, van der Horst HE, van de Ven PM, Twisk JWR, Deeg DJH, Frijters DHM, et al. Effectiveness of a Geriatric Care Model for frail older adults in primary care: Results from a stepped wedge cluster randomised trial. Eur J Intern Med 2015;28(43).

101. Ononge S, Campbell OMR, Kaharuza F, Lewis JL, Fielding K, Mirembe F. Effectiveness and safety of misoprostol distributed to antenatal women to prevent postpartum haemorrhage after child-births: a stepped-wedge cluster-randomised trial. BMC Pregnancy and Childbirth 2015;15(315).

For peer review only

PRISMA 2009 Checklist

Section/topic	#	Checklist item	Reported on page #
**TITLE**			
Title	1	Identify the report as a systematic review, meta-analysis, or both.	1-2
**ABSTRACT**			
Structured summary	2	Provide a structured summary including, as applicable: background; objectives; data sources; study eligibility criteria, participants, and interventions; study appraisal and synthesis methods; results; limitations; conclusions and implications of key findings; systematic review registration number.	12-35
**INTRODUCTION**			
Rationale	3	Describe the rationale for the review in the context of what is already known.	50-94
Objectives	4	Provide an explicit statement of questions being addressed with reference to participants, interventions, comparisons, outcomes, and study design (PICOS).	95-97
**METHODS**			
Protocol and registration	5	Indicate if a review protocol exists, if and where it can be accessed (e.g., Web address), and, if available, provide registration information including registration number.	NA
Eligibility criteria	6	Specify study characteristics (e.g., PICOS, length of follow-up) and report characteristics (e.g., years considered, language, publication status) used as criteria for eligibility, giving rationale.	103-109
Information sources	7	Describe all information sources (e.g., databases with dates of coverage, contact with study authors to identify additional studies) in the search and date last searched.	100-103
Search	8	Present full electronic search strategy for at least one database, including any limits used, such that it could be repeated.	Supplementary File 1
Study selection	9	State the process for selecting studies (i.e., screening, eligibility, included in systematic review, and, if applicable, included in the meta-analysis).	110-119
Data collection process	10	Describe method of data extraction from reports (e.g., piloted forms, independently, in duplicate) and any processes for obtaining and confirming data from investigators.	115-119
Data items	11	List and define all variables for which data were sought (e.g., PICOS, funding sources) and any assumptions and simplifications made.	120-141
Risk of bias in individual studies	12	Describe methods used for assessing risk of bias of individual studies (including specification of whether this was done at the study or outcome level), and how this information is to be used in any data synthesis.	NA
Summary measures	13	State the principal summary measures (e.g., risk ratio, difference in means).	120-141
Synthesis of results	14	Describe the methods of handling data and combining results of studies, if done, including measures of consistency (e.g., I^2) for each meta-analysis.	142-147

For peer review only

PRISMA 2009 Checklist

Section/topic	#	Checklist item	Reported on page #
Risk of bias across studies	15	Specify any assessment of risk of bias that may affect the cumulative evidence (e.g., publication bias, selective reporting within studies).	NA
Additional analyses	16	Describe methods of additional analyses (e.g., sensitivity or subgroup analyses, meta-regression), if done, indicating which were pre-specified.	NA
**RESULTS**			
Study selection	17	Give numbers of studies screened, assessed for eligibility, and included in the review, with reasons for exclusions at each stage, ideally with a flow diagram.	149-153, Figure 2
Study characteristics	18	For each study, present characteristics for which data were extracted (e.g., study size, PICOS, follow-up period) and provide the citations.	154-227, Table 1
Risk of bias within studies	19	Present data on risk of bias of each study and, if available, any outcome level assessment (see item 12).	NA
Results of individual studies	20	For all outcomes considered (benefits or harms), present, for each study: (a) simple summary data for each intervention group (b) effect estimates and confidence intervals, ideally with a forest plot.	NA
Synthesis of results	21	Present results of each meta-analysis done, including confidence intervals and measures of consistency.	NA
Risk of bias across studies	22	Present results of any assessment of risk of bias across studies (see Item 15).	NA
Additional analysis	23	Give results of additional analyses, if done (e.g., sensitivity or subgroup analyses, meta-regression [see Item 16]).	NA
**DISCUSSION**			
Summary of evidence	24	Summarize the main findings including the strength of evidence for each main outcome; consider their relevance to key groups (e.g., healthcare providers, users, and policy makers).	228-278
Limitations	25	Discuss limitations at study and outcome level (e.g., risk of bias), and at review-level (e.g., incomplete retrieval of identified research, reporting bias).	279-296
Conclusions	26	Provide a general interpretation of the results in the context of other evidence, and implications for future research.	297-320
**FUNDING**			
Funding	27	Describe sources of funding for the systematic review and other support (e.g., supply of data); role of funders for the systematic review.	321-329

From: Moher D, Liberati A, Tetzlaff J, Altman DG, The PRISMA Group (2009). Preferred Reporting Items for Systematic Reviews and Meta-Analyses: The PRISMA Statement. PLoS Med 6(7): e1000097. doi:10.1371/journal.pmed1000097

For more information, visit: www.prisma-statement.org.

For peer review only - http://bmjopen.bmj.com/site/about/guidelines.xhtml

BMJ Open

Unequal cluster sizes in stepped-wedge cluster randomised trials: a systematic review

Journal:	*BMJ Open*
Manuscript ID	bmjopen-2017-017151.R1
Article Type:	Research
Date Submitted by the Author:	01-Aug-2017
Complete List of Authors:	Kristunas, Caroline; University of Leicester, Department of Health Sciences<br>Morris, Tom; University of Leicester, Leicester Clinical Trials Unit<br>Gray, Laura; University of Leicester, Department of Health Sciences
**Primary Subject Heading**:	Research methods
Secondary Subject Heading:	Public health
Keywords:	Stepped-wedge trial, Systematic review, Cluster randomised trial

SCHOLARONE™
Manuscripts

For peer review only

Unequal cluster sizes in stepped-wedge cluster randomised trials: a systematic review

Caroline Kristunas[1*], Tom Morris[2], Laura J Gray[1]

Authors Affiliations:

[1]Department of Health Sciences, University of Leicester, Leicester, UK
[2]Leicester Clinical Trials Unit, University of Leicester, Leicester, UK

**Corresponding author:* Caroline Kristunas; cak21@le.ac.uk

Keywords: Stepped-wedge trial; systematic review; cluster randomised trial.

For peer review only

ABSTRACT

Objectives: To investigate the extent to which cluster sizes vary in stepped-wedge cluster randomised trials (SW-CRTs) and whether any variability is accounted for during the sample size calculation and analysis of these trials.

Setting: Any, not limited to healthcare settings.

Participants: Any taking part in a SW-CRT published up to March 2016.

Primary and secondary outcome measures: The primary outcome is the variability in cluster sizes, measured by the coefficient of variation in cluster size (CV). Secondary outcomes include the difference between the cluster sizes assumed during the sample size calculation and those observed during the trial, any reported variability in cluster sizes and whether the methods of sample size calculation and methods of analysis accounted for any variability in cluster sizes.

Results: Of the 101 included SW-CRTs, 48% mentioned that the included clusters were known to vary in size, yet only 13% of these accounted for this during the calculation of the sample size. However, 69% of the trials did use a method of analysis appropriate for when clusters vary in size. Full trial reports were available for 53 trials. The CV was calculated for 23 of these: the median CV was 0.41 (interquartile range: 0.22-0.52). Actual cluster sizes could be compared to those assumed during the sample size calculation for 14 (26%) of the trial reports; the cluster sizes were between 29% and 480% of that which had been assumed.

Conclusions: Cluster sizes often vary in SW-CRTs. Reporting of SW-CRTs also remains sub-optimal. The effect of unequal cluster sizes on the statistical power of SW-CRTs needs further exploration and methods appropriate to studies with unequal cluster sizes need to be employed.

STRENGTHS AND LIMITATIONS OF THIS STUDY:

- To our knowledge this is the first systematic review to assess how often and to what extent cluster sizes are unequal in stepped-wedge cluster randomised trials (SW-CRTs) and determine whether the current methodology being used accounts for any variability in cluster sizes.
- This review used pre-specified search and study selection strategies as well as using double data extraction.
- Due to poor reporting quality, the actual cluster sizes could not be extracted for all studies, and cluster sizes were therefore estimated from whatever information was provided.

For peer review only

BACKGROUND

Cluster randomised trials (CRTs) are often used to evaluate healthcare interventions that are implemented at the cluster level, for example, within hospitals, general practices or across geographical areas, where treatment contamination would be likely to occur if individual randomisation was used.[1] In conventional parallel CRTs, clusters are generally randomised to either a control or intervention group (usually with equal numbers in each group). After baseline measurements have been taken, those randomised to the intervention group are exposed to the intervention for the remainder of the trial, whereas those in the control group are not.

The stepped-wedge CRT (SW-CRT) is a relatively recent variant of the CRT. In SW-CRTs the clusters are randomised to start the intervention at different time points, known as steps, rather than being randomised to either a control or an intervention group. All of the clusters generally start the trial in a control group, then switch to the intervention when their time comes, until by the end of the trial all of the clusters are exposed to the intervention.[2] The cluster size is the number of individuals providing outcome measurements, either per cluster per measurement point, or per cluster over the whole duration of the trial. Figure 1 gives a schematic representation of a SW-CRT. Although use of this trial design is still relatively rare, recent systematic reviews have shown there to be a dramatic rise in the number of SW-CRTs published within the last few years.[3] However, the methodological advances for SW-CRTs lag behind those for other trial designs.

Due to the variability that occurs in the natural sizes of some clusters, the actual observed cluster sizes often vary within a CRT.[1] For both cohort and cross-sectional designs the actual observed cluster size can be defined as either the number of observations actually accrued over the whole duration of the trial or the number of observations actually accrued at each measurement point, with the latter enabling the first to be calculated. Clusters may also vary in size due to differences in the rates of loss to follow-up and incidence rates across clusters.[1] Increasing variability in cluster sizes has been shown to cause a loss of statistical power in parallel CRTs,[4] particularly if the coefficient of variation in cluster size (CV) (given as the standard deviation of the cluster size divided by the mean cluster size) is greater than 0.23.[5] This can lead to a trial that is under-powered to detect the effect of the intervention that is being evaluated. Various sample size calculation methods have thus been developed for parallel CRTs which account for any imbalance in cluster sizes, to ensure that the trial will be sufficiently powered.[5-8] However, although the effect of unequal cluster sizes has been reported for parallel CRTs, it has only been reported for cross-sectional SW-CRTs with a continuous outcome and analysed using generalised estimating equations (GEEs).[9] It is expected that some loss of power will be observed for certain sub-types of SW-CRTs and therefore a similar adjustment to the sample size calculation is likely to be required.

It has been recommended that mixed-effects models (MMs) or GEEs should be used to analyse SW-CRTs.[10] In particular, linear MMs can be used for normal data or when cluster sizes are approximately equal, but if cluster sizes vary and the outcome is non-normal, then generalised linear MM (GLMM) or GEEs are preferred.[10] This is because both of these methods can account for the variability in cluster sizes, as well as non-

For peer review only

normal data.[10] It is therefore to be expected that MMs and GEEs will be employed in SW-CRTs where the cluster sizes are likely to vary. Other methods of analysis that can also account for the design features of SW-CRTs (such as clustering and potential time effects) may also be suitable for use when cluster sizes vary, however, the appropriateness of these methods for the analysis of SW-CRTs with variable cluster sizes is currently unknown. To date, MMs and GEEs are the only methods of analysis for which the efficiency of the method has been investigated when cluster sizes vary.

Varying cluster sizes should also be considered during the randomisation process, as larger clusters may have different characteristics to smaller clusters. The randomisation process should therefore be such that it ensures that cluster sizes are balanced across randomisation groups (clusters randomised to switch to the intervention at the same time).

A recent systematic review of SW-CRTs found that few studies reported any variation in cluster sizes. However, major deficiencies were found in the reporting [11] and so this is likely to be an underestimation of the true extent of variability in cluster sizes. The size of the variability in cluster sizes in SW-CRTs has not previously been reported, nor how any imbalance in cluster sizes is being adjusted for during the calculation of the sample size or the analysis of these trials. The primary aims of our review were therefore to:

- Determine how often and to what extent cluster sizes are unequal in SW-CRTs.
- Determine how unequal cluster sizes are being taken into account during the calculation of the sample size and the analysis of SW-CRTs.

METHODS

Search strategy

We included in our review studies identified by a recent systematic review conducted by Martin et al of SW-CRTs up to 23 October 2014.[11] In addition, we also included studies published more recently, by searching MEDLINE, Scopus and EBSCO Host, up to 21 March 2016, using an adapted version of the Martin et al search strategy (adapted to each database and to exclude studies prior to 23 October 2014). The search strategy is provided in Supplementary File 1. The studies that were included were from protocols or independent full reports of SW-CRTs in both healthcare and non-healthcare settings.[11] The studies had to be randomised trials, using cluster randomisation, and have at least two steps. We did not restrict our search to trials where all clusters started in the control condition or ended in the intervention condition. Trials that were not published in English, were individually or non-randomised trials, trials with a cross-over design and those that were retrospectively analysed as a SW-CRT were excluded.[11] We focused on original research studies and primary study reports.

Once duplicates had been removed, the titles and abstracts of the studies identified by the search were screened for eligibility by CK. Full-text articles were then obtained for all potentially eligible studies and screened independently by two authors (CK and TM). Those studies found not to meet the eligibility criteria were excluded and the reasons for exclusion tabulated. Any differences of opinion were resolved by discussions with LG.

Data extraction

For peer review only

Data for all studies meeting the eligibility criteria were extracted by CK and checked by TM. Any differences of opinion were resolved through discussions with all authors. A data extraction form was developed, then tested and refined on a small number of studies.

Basic trial characteristics are reported. These include the year of publication, type of cluster, the type of primary outcome (binary, continuous, time-to-event etc.), number of clusters required and whether any restrictions related to cluster sizes were used in the randomisation procedure.

Information was also extracted in relation to variability in cluster sizes. We report whether it was mentioned in the report or protocol that clusters were known to vary in size prior to the trial commencing, whether any variability in cluster sizes was accounted for during the randomisation process or calculation of the sample size, and whether the method of analysis appropriately accounts for this variability. We also recorded the number and sizes of the clusters assumed during the sample size calculation. For completed trials we extracted the actual cluster sizes and reported the variability in cluster sizes that was observed during the trial, and made a comparison of the actual cluster sizes with the cluster sizes that were assumed during the sample size calculations.

Reporting of SW-CRTs is poor [11, 12] and so to increase the number of trials available for a comparison of cluster sizes, some cluster sizes had to be estimated from whatever information was available. Cluster sizes per time period, at a specific time point (such as at baseline) or the total cluster sizes over the full duration of the trial were preferred. If this information was not available then other information relating to the sizes of the clusters was used to estimate the cluster sizes, so that an estimate of the variability in the sizes of the clusters could be made. This included using the reported number of eligible individuals in each cluster as an approximation of the number of individuals included in each cluster, assuming that all clusters in a randomisation group (clusters randomised to switch to the intervention at the same time) were of equal size when only the mean cluster size per randomisation group was given, or using the mean and standard deviation of cluster size in each randomisation group to find the CV for each group. The comparison of cluster sizes is reported separately for the actual cluster sizes and those that were estimated from summary measures.

Analysis

We summarise the basic trial characteristics, methods of sample size calculation and methods of analysis, and number and size of clusters assumed during the sample size calculation for study reports and protocols. Actual cluster sizes, estimated actual cluster sizes and comparisons of actual cluster sizes with those that were assumed during the calculation of the sample size are summarised for trial reports only.

RESULTS

We identified 371 records through our search of electronic databases. After combining this with the records identified by Martin et al,[11] removing three protocols for which the full reports had been identified, and after removing duplicates, we were left with 330 records. We screened these records and excluded 229 which did not meet our eligibility criteria (Figure 2). A full list of the 101 included studies is given in Supplementary File 2.

For peer review only

The general characteristics of the included trials are presented in Table 1. Full trial reports were available for 53 (52%) trials, with only protocols available for the remaining 48 (48%) trials. Two (2%) trials were conducted as pilot studies. A primary outcome was reported for 87 (86%) trials, of which the majority were binary (61%) or continuous (16%) outcomes and six (7%) reported multiple primary outcomes. Three (3%) trials did not report a primary outcome and for the remaining 11 (11%) trials the primary outcome was not clear. For the majority of the trials (52%), the clusters were comprised of health care facilities, such as hospitals, hospital wards, clinics or GP practices. The remaining clusters comprised of geographical areas/communities (10%), residential/long-term care facilities (9%), health care professionals (9%), educational facilities (6%), rehabilitation clinics/programs (5%) family groups/households (4%), or other groups (5%).

Table 1: Trial characteristics of included SW-CRTs. Values are number/denominator (percentage), unless otherwise stated. The denominator is omitted whenever it is the number of included studies (n=101).

Characteristic	
**Publication type**	
Report	53 (52.5%)
Protocol	48 (47.5%)
Pilot study	2 (2.0%)
**Primary outcome reported**	**87 (86.1%)**
Binary	53/87 (60.9%)
Continuous	14/87 (16.1%)
Count	7/87 (8.0%)
Ordinal	6/87 (6.9%)
Multiple	6/87 (6.9%)
Time-to-event	1/87 (1.1%)
**Types of cluster**	
Health care facilities	53 (52.5%)
Geographical areas/communities	10 (9.9%)
Residential/long-term care facilities	9 (8.9%)
Health care professionals (including groups thereof)	9 (8.9%)
Education facilities	6 (5.9%)
Rehabilitation clinics/programs	5 (5.0%)
Family groups/households	4 (4.0%)
Other	5 (5.0)%
Evidence of clusters being known to vary in size	48 (47.5%)
Sample size calculation presented	82 (81.2%)
**Accounted for unequal cluster sizes in sample size calculation**	**6 (5.9%)**
Used coefficient of variation in cluster size	2/6 (33.3%)
Accounted for in simulation	2/6 (33.3%)
Unclear	2/6 (33.3%)
**Reported number of clusters required**	**71 (70.3%)**
Median (interquartile range) number of clusters required (n=71)	11 (6 – 22.5)

For peer review only

**Reported required cluster size**	**57 (56.4%)**
Median (interquartile range) per time period (n=23)	30 (20 - 125)
Median (interquartile range) overall (n=34)	51 (20.75 – 165)
Reported total sample size required[1]	64 (63.4%)
Accounted for unequal cluster sizes in the randomisation	29 (28.7%)
**Method of analysis reported**	**94 (93.1%)**
GEEs	13/94 (12.9%)
MM	57/94 (56.4%)
GLMM	32/94 (31.7%)
LMM	7/94 (6.9%)
Unclear	18/94 (17.8%)
Other models	16/94 (15.8%)
Generalised LM	5/94 (5.0%)
GLM (accounting for clustering)	5/94 (5.0%)
LM/ANCOVA	6/94 (5.9%)
Simple analysis (McNemar's, Mann-Whitney U, t-test, ANOVA, bivariate, Wilcoxon signed-rank)	8/94 (7.9%)
Unclear	5/94 (5.0%)
Not given	2/94 (2.0%)

[1] *Total sample size required either stated or enough detail given for it to be reproduced.*

For just under half (48, 48%) of the trials there was evidence that the clusters were known to vary in size. Yet few of these accounted for inequality in cluster size during the randomisation process (29%) or sample size calculation (13%). Nine stratified by cluster size, one pair-matched according to cluster size, two restricted randomisation by cluster size, one used minimisation to balance cluster sizes and one used block randomisation by cluster size. One additional trial used stratification, but did not state for which variables stratification was applied.

Sample size calculation

Only 82 (81%) of the included studies presented a sample size calculation, of which 71 reported the number of clusters required and 57 reported the required cluster sizes (23 reported cluster size per time period, 34 reported total cluster size). The median (interquartile range (IQR)) number of clusters required for these trials was 11 (6 – 22.5). The median (IQR) cluster size was 30 (20 - 125) for those that gave the cluster size per time period and 51 (20.75-165) for those that gave the total cluster size over the duration of the trial.

In total only six (6%) trials reported that they had accounted for unequal cluster sizes when calculating the required sample size. Two used an estimate of the coefficient of variation in cluster size, two used simulations to calculate the required sample size, varying the cluster sizes in their simulations, and for the remaining two trials the method used to account for unequal cluster sizes was unclear.

Methods of analysis

A small proportion of the trials (12%) attempted to adjust for any inequality in cluster size through the inclusion of covariates in the analysis and one trial planned to conduct

For peer review only

an individual level analysis, rather than cluster level analysis, if clusters were found to vary in size.

The majority of analyses were conducted using mixed-effects models (MM) (56%), with 32 studies using generalised linear MMs, seven using linear MMs and 18 not distinguishing between the two. An equal number of trials used generalised estimating equations (13%) as used simple regression models (linear or generalised linear models) (13%). Only five of those that used simple regression models accounted for the clustering of the data during the analysis. The method of analysis for the primary outcome was unclear for five studies and two studies did not report in their protocol details of how the analysis would be conducted. The remaining 11 (11%) studies employed simple analysis methods, such as ANOVA and two sample t-tests, which did not account for the clustering of the data nor any period effect. The majority of studies (69%) employed a method of analysis known to be appropriate for when cluster sizes vary in SW-CRTs (LMM, GLMM or GEEs). Of the 48 studies for which there was evidence that cluster sizes were known to vary, 75.0% (36) of those used LMM, GLMM or GEEs in the analysis. Whereas, for those 23 trials where the cluster sizes were actually found to vary in size only 12 (52.2%) of them used LMM, GLMM or GEEs.

Reporting of actual cluster sizes

Of the 53 included full trial reports, it was only possible to determine the actual cluster sizes for 13 (25%) of the trials. Five of these trials gave the cluster sizes during each time period, four gave the size of each cluster at a specific time point, such as at baseline or the point of analysis and the remaining four reports gave the total size of each cluster over the duration of the whole trial. One further trial gave the number of eligible individuals in each cluster. A further eight (15%) reports gave a summary measure of the cluster sizes, such as the mean per month, per randomisation group, per intervention group etc. or the range of cluster sizes. Two (4%) reports gave the sizes of the randomisation groups only.

Comparison of cluster sizes

Few study reports presented both the required cluster sizes and the actual cluster sizes. The way in which cluster sizes were presented varied between studies and some only presented summary measures of the cluster sizes. Of the 24 trial reports that presented some indication of the cluster sizes, only 14 (58%) also stated the required cluster size. For those trials that presented the actual cluster sizes, the individual cluster sizes, either over the whole trial or at each time point, were between 29% and 480% of the required cluster size (median=100%, IQR=90%, 100%). For those trials that reported a summary measure of the actual cluster sizes, the cluster sizes were between 30% and 209% of the required cluster size (median=79%, IQR=65%, 101%). The average cluster size for each trial ranged between 66% and 214% of the required cluster size (median=95%, IQR=79%, 116%).

None of the trial reports presented the actual value of the coefficient of variation (CV) in cluster size. It was possible to estimate the CV in cluster size for 23 of the trials, of which only two experienced no variability in cluster size. The median CV in cluster size was 0.41 (IQR: 0.22-0.52) and the maximum value was 1.29. . None of these trials reported that they accounted for unequal cluster sizes during the sample size calculation.

For peer review only

DISCUSSION

We conducted a systematic review to assess how often and to what extent cluster sizes are unequal in SW-CRTs, and to determine how any inequality in cluster sizes is currently being taken into account during the calculation of the sample sizes and the analysis of these trials. Of the 101 SW-CRTs included, almost half (48%) mentioned that the clusters were known to vary in size, yet many of these did not accounted for this during the calculation of the sample size. Simple analytical methods of sample size calculation which account for any variability in cluster sizes exist for parallel CRTs.[5-8] No such methods have been developed for use with SW-CRTs, though it may be possible to adapt the methods for parallel CRTs for use in SW-CRTs.

About 69% of the trials did however employ a method of analysis deemed appropriate for when cluster sizes are unequal in SW-CRTs (LMM, GLMM or GEEs).[10] There may be other methods of analysis that are also suitable for SW-CRTs with unequal cluster sizes, but these have yet to be investigated. In addition to the suitability of the analysis method to the variability in cluster sizes in a SW-CRT, the appropriateness of the method to the SW-CRT design must also be considered. Clustering and time effects will also need to be considered as well. Barker et al [13] and Davey et al [14] have both conducted reviews that consider the suitability of analysis methods for SW-CRTs.

Full trial reports were available for 53 trials. Only a quarter of these reported the sizes of each cluster, either per time period, at a specific point during the trial, or the total size over the whole duration of the trial. The CV in cluster size was calculated based on this information. A further eight trials reported a summary measure of the cluster sizes, such as the mean cluster size per month or the mean cluster size per randomisation group. From these summary measures we were able to produce an estimate of the CV in cluster size for each of these trials. One further trial only gave the number of eligible individuals in each cluster and so, assuming equal recruitment rates in each cluster, it was also possible to estimate the CV in cluster size for this trial. Better reporting of CV is required for SW-CRTs, whether this be the CV across all periods or the CV per period.

Only two trials experienced no variability in cluster size. The majority (70%) of trials had a CV in cluster size >0.23 and so if these had been parallel CRTs we would have expected these trials to have suffered a significant loss of statistical power.[5] However, a recent simulation study has shown that for cross-sectional SW-CRTs with a continuous outcome and intracluster correlation coefficient (ICC) of 0.05, even high variability in cluster sizes does not cause a significant loss of power.[9] Nevertheless, a recent systematic review found that the majority of SW-CRTs are not of a cross-sectional design [12] and within health research an ICC of 0.05 is generally considered high. It is for these cohort, low ICC, and other trials that the effect of unequal cluster sizes is still unknown. However, even if unequal cluster sizes are found to cause a reduction in power for most SW-CRTs, this effect may be overshadowed by the effect of using an incorrect sample size calculation, such as one that does not allow for the effect of time. Not allowing for the effect of time in this calculation can result in both under- and over-powered studies and so even with unequal cluster sizes, a study might still be over-powered.[11] It is therefore important that the sample size calculation for a SW-CRT correctly allows for all aspects of the design.

For peer review only

The actual cluster sizes also varied considerably from those that were assumed during the calculation of the required sample size, with reported cluster sizes being between 29% and 480% of that which was required. This would suggest that many of the trials we examined are likely to have been considerably over- or under-powered. These comparisons could only be made for 14 (26%) of the trials with published reports. Information both on the sizes of the clusters that were assumed during the calculation of the sample size and the actual cluster sizes that were observed during the trial were often not reported.

The majority of systematic reviews of SW-CRTs have been conducted with the aim of assessing the quality and breadth of SW-CRTs.[3, 15, 16] A couple of recent systematic reviews have also been conducted to assess the quality of reporting and methodological rigor of SW-CRTs,[11, 12] as well as the statistical methodology being used and available for these trials.[13] However, none of these systematic reviews have assessed the prevalence and severity of unequal cluster sizes in SW-CRTs, nor the statistical methodology being used to account for this variability in cluster sizes. Our review has highlighted the high degree of variability in cluster sizes that is observed in SW-CRTs. Knowing that cluster sizes often vary in SW-CRTs can allow researchers to plan for an expected inequality in cluster sizes, taking this into account when calculating the required sample size for these trials and choosing an appropriate method of analysis. We also highlight the need for improved quality of reporting of cluster sizes in SW-CRTs, which concurs with the findings of previous reviews [11, 12] and the need for wider implementation of methodologies which account for unequal cluster sizes. These findings are in line with what has been shown more generally in CRTs.[17]

Our study has several strengths. In order to minimise the potential for bias during the review, we pre-specified search and study selection strategies. We also did not limit our search to publications from journals with a high impact factor, increasing the generalisability of our findings. However, our study does have limitations as well. For example, selection bias may have been introduced by the choice to only include studies published in English. It was also difficult to ensure the identification of all SW-CRTs, as many do not include in either their title or abstract the common terms that identify a SW-CRT. We included in our search strategy all of the common identifiers that have been included in previous reviews, yet this is still not sufficient to identify studies where the researchers weren't aware that their studies were SW-CRTs and therefore did not include common identifiers in their title or abstract. This may introduce some selection bias. The poor reporting of SW-CRTs [11, 12] may limit the generalisability of the findings of our study. It was only possible to make a comparison of cluster sizes and to calculate the CV in cluster size for a small number of studies, and these are likely to be those studies that have a better quality of reporting than the other studies. The estimations that were made when determining the observed cluster sizes may also result in an under- or over-estimation of the degree of variability in cluster sizes and the comparability of the observed cluster sizes with those which were required.

Unequal cluster sizes is a common problem in SW-CRTs. Variability in cluster sizes is not being accounted for during the calculation of the sample size for these trials and the reporting of the actual cluster sizes observed during these trials is poor, which limits the scope of this review. Appropriate methods of sample size calculation that account for

For peer review only

variability in cluster sizes should be developed by methodological statisticians and perhaps implemented in appropriate software in order to allow their widespread use. Simulation methods, such as those used by Baio et al, [18] could be used whilst simple analytical methods are developed. Further research is also required to investigate the effect of unequal cluster sizes on the statistical power for cohort SW-CRTs and those with a binary outcome. An investigation into how our results differ between cohort and cross-sectional designs would also be of interest.

We echo the views of others,[2] that the development of reporting guidelines for SW-CRTs is needed, particularly to improve the sub-optimal quality of reporting for the sample size calculations and of the actual observed cluster sizes in SW-CRTs. In the meantime it is recommended that reporting should follow the CONSORT 2010 extension to cluster randomised trials [19] and the modifications presented by Hemming et al in their paper.[2] A CONSORT extension to stepped-wedge cluster randomised trials is currently in development.[20]

CONCLUSIONS

Cluster sizes were found to vary in SW-CRTs. The effect of unequal cluster sizes on SW-CRTs is yet to be reported for cohort designs and those with a binary outcome and so the effect of this observed variability is currently unknown. Methods of sample size calculation that are used for these trials rarely account for any variability in cluster size. Reporting of cluster sizes in both the reported sample size calculation and those actually observed during the trial is poor. Authors need to provide greater detail of how the required sample size has been calculated as well as providing details of the actual cluster sizes observed during the trial, preferably at each step of the trial. Researchers should also be aware that cluster sizes are likely to vary in SW-CRTs and so sample size calculation methods and analysis methods should be used which can account for this variability.

FUNDING

CK is funded by a National Institute for Health Research (NIHR) Research Methods Fellowship. The authors would also like to acknowledge support from the NIHR Collaboration for Leadership in Applied Health Research and Care – East Midlands (NIHR CLAHRC – EM), Leicester Clinical Trials Unit and the NIHR Leicester-Loughborough Diet, Lifestyle and Physical Activity Biomedical Research Unit, which is a partnership between University Hospitals of Leicester NHS Trust, Loughborough University and the University of Leicester. The views expressed are those of the authors and not necessarily those of the NIHR or the Department of Health.

COMPETING INTERESTS

None declared.

DATA SHARING

No additional data are available.

CONTRIBUTORS

LJG conceptualised the review. CK carried out the systematic review, designed the data extraction form, identified the studies for inclusion, extracted data, performed the data analysis and wrote the first draft. TM carried out the second data extraction and critically reviewed the contents of the paper. LJG resolved any differences between CK and TM in the data extraction. All authors contributed to the writing of the paper, read and approved the final manuscript.

REFERENCES

1 Eldridge S, Kerry S. A Practical Guide to Cluster Randomised Trials in Health Services Research. US: John Wiley & Sons Inc 2012.

2 Hemming K, Haines TP, Chilton PJ, et al. The stepped wedge cluster randomised trial: rationale, design, analysis, and reporting, *BMJ* 2015;350:h391 doi:10.1136/bmj.h391.

3 Beard E, Lewis J, Copas A, et al. Stepped wedge randomised controlled trials: systematic review of studies published between 2010 and 2014, *Trials* 2015;16:1-14.

4 Lauer SA, Kleinman KP, Reich NG. The Effect of Cluster Size Variability on Statistical Power in Cluster-Randomized Trials, *PLoS ONE* 2015;10:e0119074.

5 Eldridge SM, Ashby D, Kerry S. Sample size for cluster randomized trials: effect of coefficient of variation of cluster size and analysis method, *Int J Epidemiol* 2006;35:1292-300.

6 Kerry SM, Bland JM. Unequal cluster sizes for trials in English and Welsh general practice: implications for sample size calculations, *Stat Med* 2001;20:377-90 doi:AID-SIM799>3.0.CO;2-N.

7 Manatunga AK, Hudgens MG, Chen S. Sample Size Estimation in Cluster Randomized Studies with Varying Cluster Size, *Biometrical Journal* 2001;43:75-86.

8 Pan W. Sample Size and Power Calculations with Correlated Binary Data, *Control Clin Trials* 2001;22:211-27.

9 Kristunas CA, Smith KL, Gray LJ. An imbalance in cluster sizes does not lead to notable loss of power in cross-sectional, stepped-wedge cluster randomised trials with a continuous outcome, *Trials* 2017;18:109.

10 Hussey MA, Hughes JP. Design and analysis of stepped wedge cluster randomized trials, *Contemporary Clinical Trials* 2007;28:182-91.

For peer review only

11 Martin J, Taljaard M, Girling A, et al. Systematic review finds major deficiencies in sample size methodology and reporting for stepped-wedge cluster randomised trials, *BMJ open* 2016;6:e010166 doi:10.1136/bmjopen-2015-010166.

12 Grayling MJ, Wason JM, Mander AP. Stepped wedge cluster randomized controlled trial designs: a review of reporting quality and design features, *Trials* 2017;18:33.

13 Barker D, McElduff P, D'Este C, et al. Stepped wedge cluster randomised trials: A review of the statistical methodology used and available, *BMC Medical Research Methodology* 2016;16 doi:10.1186/s12874-016-0176-5.

14 Davey C, Hargreaves J, Thompson JA, et al. Analysis and reporting of stepped wedge randomised controlled trials: Synthesis and critical appraisal of published studies, 2010 to 2014, *Trials* 2015;16.

15 Brown C, Lilford R. The stepped wedge trial design: a systematic review, *BMC Medical Research Methodology* 2006;6:54.

16 Mdege ND, Man M, Taylor (nee Brown), Celia A, et al. Systematic review of stepped wedge cluster randomized trials shows that design is particularly used to evaluate interventions during routine implementation, *J Clin Epidemiol* 2011;64:936-48.

17 Rutterford C, Taljaard M, Dixon S, et al. Reporting and methodological quality of sample size calculations in cluster randomized trials could be improved: a review, *J Clin Epidemiol* 2015;68:716-23.

18 Baio G, Copas A, Ambler G, et al. Sample size calculation for a stepped wedge trial, *Trials* 2015;16:354.

19 Campbell MK, Piaggio G, Elbourne DR, et al. Consort 2010 statement: Extension to cluster randomised trials, *BMJ (Online)* 2012;345.

20 Hemming K, Girling A, Haines T, et al. Protocol: Consort extension to stepped wedge cluster randomised controlled trial, *BMJ* 2014.

FIGURE LEGENDS

Figure 1: Schematic representation of a stepped-wedge cluster randomised trial with four steps. The white areas represent control periods and the shaded areas represent intervention periods.

Figure 2: Flow chart showing the studies identified by the systematic review.

For peer review only

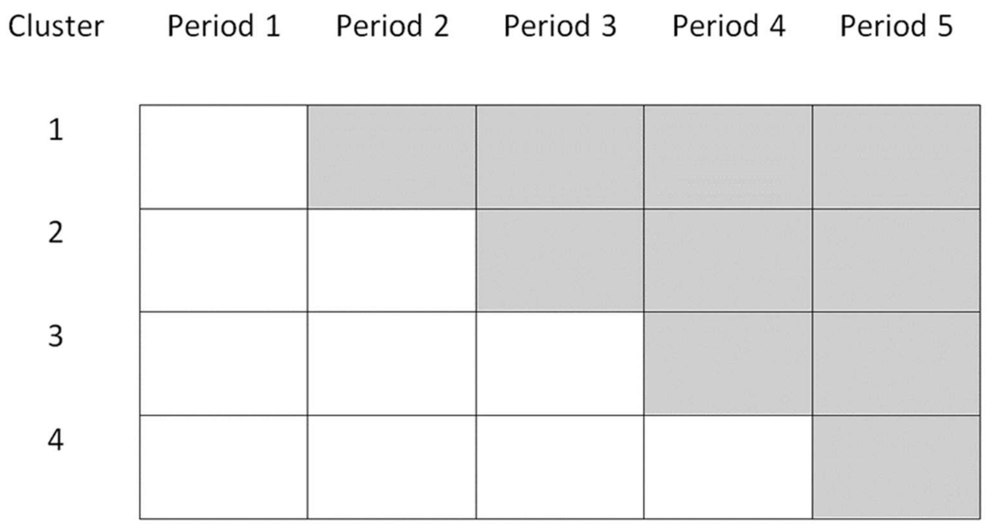

Schematic representation of a stepped-wedge cluster randomised trial with four steps. The white areas represent control periods and the shaded areas represent intervention periods.

190x107mm (300 x 300 DPI)

For peer review only

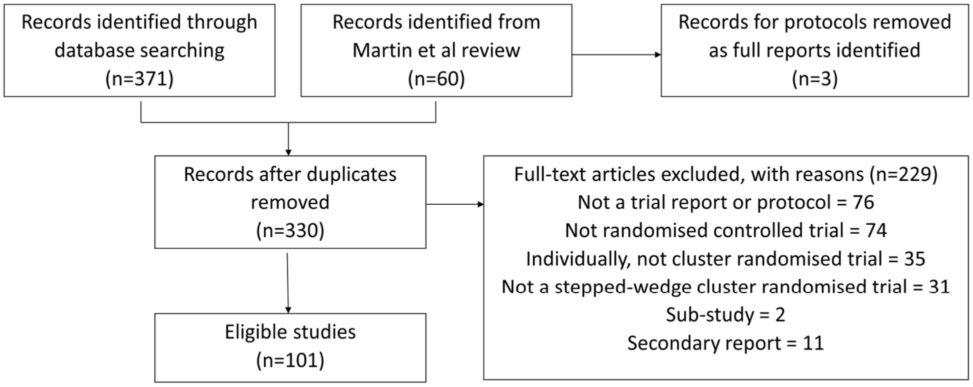

Flow chart showing the studies identified by the systematic review.

190x107mm (300 x 300 DPI)

review only

SYSTEMATIC REVIEW SEARCH STRATEGY

1. step$ wedge.ti,ab.
2. experimentally staged introduction.ti,ab.
3. delayed intervention.ti,ab.
4. (one directional cross over design or one directional crossover design).ti,ab.
5. ((incremental or phased or staggered or stepwise or step wise or delayed) adj1 (recruitment or introduction or implementation)).ti,ab.
6. or/1-5
7. limit 6 to english language
8. limit 7 to randomized controlled trial
9. limit 8 to humans
10. limit 9 to yr=”2014-current”

For peer review only

Reference list of included studies

1. Craine N, Whitaker R, Perrett S, Zou L, Hickman M, Lyons M. A stepped wedge cluster randomized control trial of dried blood spot testing to improve the uptake of hepatitis C antibody testing within UK prisons. European journal of public health. 2014.

2. Palmay L, Elligsen M, Walker SAN, Pinto R, Walker S, Einarson T, et al. Hospital-wide rollout of antimicrobial stewardship: A stepped-wedge randomized trial. Clinical Infectious Diseases. 2014;59(6):867-74.

3. van de Steeg L, R IJ, Langelaan M, Wagner C. Can an e-learning course improve nursing care for older people at risk of delirium: a stepped wedge cluster randomised trial. BMC geriatrics. 2014;14:69.

4. Haugen AS, Softeland E, Almeland SK, Sevdalis N, Vonen B, Eide GE, et al. Effect of the World Health Organization Checklist on Patient Outcomes: A Stepped Wedge Cluster Randomized Controlled Trial. Annals of surgery. 2014.

5. Stern A, Mitsakakis N, Paulden M, Alibhai S, Wong J, Tomlinson G, et al. Pressure ulcer multidisciplinary teams via telemedicine: a pragmatic cluster randomized stepped wedge trial in long term care. BMC Health Serv Res. 2014;14:83.

6. Enns E, Rhemtulla R, Ewa V, Fruetel K, Holroyd-Leduc JM. A controlled quality improvement trial to reduce the use of physical restraints in older hospitalized adults. Journal of the American Geriatrics Society. 2014;62(3):541-5.

7. Roy A, Anaraki S, Hardelid P, Catchpole M, Rodrigues LC, Lipman M, et al. Universal HIV testing in London tuberculosis clinics: A cluster randomised controlled trial. European Respiratory Journal. 2013;41(3):627-34.

8. Mhurchu CN, Gorton D, Turley M, Jiang Y, Michie J, Maddison R, et al. Effects of a free school breakfast programme on children's attendance, academic achievement and short-term hunger: Results from a stepped-wedge, cluster randomised controlled trial. Journal of Epidemiology and Community Health. 2013;67(3):257-64.

9. Leontjevas R, Gerritsen DL, Smalbrugge M, Teerenstra S, Vernooij-Dassen MJFJ, Koopmans RTCM. A structural multidisciplinary approach to depression management in nursing-home residents: A multicentre, stepped-wedge cluster-randomised trial. The Lancet. 2013;381(9885):2255-64.

10. Gruber JS, Reygadas F, Arnold BF, Ray I, Nelson K, Colford Jr JM. A stepped wedge, cluster-randomized trial of a household UV-disinfection and safe storage drinking water intervention in rural Baja California Sur, Mexico. American Journal of Tropical Medicine and Hygiene. 2013;89(2):238-45.

11. Durovni B, Saraceni V, Moulton LH, Pacheco AG, Cavalcante SC, King BS, et al. Effect of improved tuberculosis screening and isoniazid preventive therapy on incidence of tuberculosis and death in patients with HIV in clinics in Rio de Janeiro, Brazil: A stepped wedge, cluster-randomised trial. The Lancet Infectious Diseases. 2013;13(10):852-8.

12. Bashour HN, Kanaan M, Kharouf MH, Abdulsalam AA, Tabbaa MA, Cheikha SA. The effect of training doctors in communication skills on women's satisfaction with doctor-woman relationship during labour and delivery: A stepped wedge cluster randomised trial in Damascus. BMJ Open. 2013;3(8).

13. van den Broek IV, van Bergen JE, Brouwers EE, Fennema JS, Gotz HM, Hoebe CJ, et al. Effectiveness of yearly, register based screening for chlamydia in the Netherlands: Controlled trial with randomised stepped wedge implementation. BMJ: British Medical Journal. 2012;345(7869):1-12.

14. Monse B, Duijster D, Sheiham A, Grijalva-Eternod CS, van Palenstein Helderman W, Hobdell MH. The effects of extraction of pulpally involved primary teeth on weight, height and BMI in underweight Filipino children. A cluster randomized clinical trial. BMC public health. 2012;12:725.

15. Horner C, Wilcox M, Barr B, Hall D, Hodgson G, Parnell P, et al. The longitudinal prevalence of MRSA in care home residents and the effectiveness of improving infection prevention knowledge and practice on colonisation using a stepped wedge study design. BMJ Open. 2012;2(1):e000423.

16. Fuller C, Michie S, Savage J, McAteer J, Besser S, Charlett A, et al. The Feedback Intervention Trial (FIT)--improving hand-hygiene compliance in UK healthcare workers: a stepped wedge cluster randomised controlled trial. PloS one. 2012;7(10):e41617.

For peer review only

17. Chinbuah MA, Kager PA, Abbey M, Gyapong M, Awini E, Nonvignon J, et al. Impact of community management of fever (using antimalarials with or without antibiotics) on childhood mortality: A cluster-randomized controlled trial in Ghana. American Journal of Tropical Medicine and Hygiene. 2012;87(SUPPL.5):11-20.

18. Etchells E, Adhikari NK, Wu R, Cheung M, Quan S, Mraz R, et al. Real-time automated paging and decision support for critical laboratory abnormalities. BMJ quality & safety. 2011;20(11):924-30.

19. Bacchieri G, Barros AJ, Santos JV, Goncalves H, Gigante DP. A community intervention to prevent traffic accidents among bicycle commuters. Revista de saude publica. 2010;44(5):867-75.

20. Solomon E, Rees T, Ukoumunne OC, Metcalf B, Hillsdon M. The Devon Active Villages Evaluation (DAVE) trial of a community-level physical activity intervention in rural south-west England: A stepped wedge cluster randomised controlled trial. The International Journal of Behavioral Nutrition and Physical Activity. 2014;11:94.

21. Gozalo P, Prakash S, Qato DM, Sloane PD, Mor V. Effect of the bathing without a battle training intervention on bathing-associated physical and verbal outcomes in nursing home residents with dementia: A randomized crossover diffusion study. Journal of the American Geriatrics Society. 2014;62(5):797-804.

22. Foster JM, Hoskins G, Smith B, Lee AJ, Price D, Pinnock H. Practice development plans to improve the primary care management of acute asthma: randomised controlled trial. BMC Fam Pract. 2007;8:23.

23. Flannery DJ, Vazsonyi AT, Liau AK, Guo S, Powell KE, Atha H, et al. Initial behavior outcomes for the peacebuilders universal school-based violence prevention program. Developmental psychology. 2003;39(2):292-308.

24. Priestley G, Watson W, Rashidian A, Mozley C, Russell D, Wilson J, et al. Introducing Critical Care Outreach: A ward-randomised trial of phased introduction in a general hospital. Intensive Care Medicine. 2004;30(7):1398-404.

25. Husaini BA, Reece MC, Emerson JS, Scales S, Hull PC, Levine RS. A church-based program on prostate cancer screening for African American men: reducing health disparities. Ethn Dis. 2008;18(2 Suppl 2):S2-179-84.

26. Viera AJ, Garrett JM. Preliminary study of a school-based program to improve hypertension awareness in the community. Family medicine. 2008;40(4):264-70.

27. Barton A, Basham M, Foy C, Buckingham K, Somervill M. The Watcombe Housing Study: the short term effect of improving housing conditions on the health of residents. J Epidemiol Community Health. 2007;61(9):771-7.

28. Bailet LL, Repper KK, Piasta SB, Murphy SP. Emergent literacy intervention for prekindergarteners at risk for reading failure. Journal of Learning Disabilities. 2009;42(4):336-55.

29. Weiner M, El Hoyek G, Wang L, Dexter PR, Zerr AD, Perkins AJ, et al. A web-based generalist-specialist system to improve scheduling of outpatient specialty consultations in an academic center. Journal of general internal medicine. 2009;24(6):710-5.

30. Brown BB, Young J, Smith DP, Kneebone AB, Brooks AJ, Xhilaga M, et al. Clinician-led improvement in cancer care (CLICC)--testing a multifaceted implementation strategy to increase evidence-based prostate cancer care: phased randomised controlled trial--study protocol. Implementation science : IS. 2014;9:64.

31. Reuther S, Holle D, Buscher I, Dortmann O, Muller R, Bartholomeyczik S, et al. Effect evaluation of two types of dementia-specific case conferences in German nursing homes (FallDem) using a stepped-wedge design: Study protocol for a randomized controlled trial. Trials. 2014;15(1).

32. Bernabe-Ortiz A, Diez-Canseco F, Gilman RH, Cardenas MK, Sacksteder KA, Miranda JJ. Launching a salt substitute to reduce blood pressure at the population level: A cluster randomized stepped wedge trial in Peru. Trials. 2014;15(1).

33. Brimblecombe J, Ferguson M, Liberato SC, Ball K, Moodie ML, Magnus A, et al. Stores Healthy Options Project in Remote Indigenous Communities (SHOP@RIC): a protocol of a randomised trial promoting healthy food and beverage purchases through price discounts and in-store nutrition education. BMC Public Health. 2013;13:744.

For peer review only

34. Feng R, Li K, Cheng J, Xie S, Chai J, Wei P, et al. Toward integrated and sustainable prevention against diabetes in rural China: Study rationale and protocol of eCROPS. BMC Endocrine Disorders. 2013;13(28).

35. Keriel-Gascou M, Buchet-Poyau K, Duclos A, Rabilloud M, Figon S, Dubois JP, et al. Evaluation of an interactive program for preventing adverse drug events in primary care: study protocol of the InPAct cluster randomised stepped wedge trial. Implementation science : IS. 2013;8:69.

36. Praveen D, Patel A, McMahon S, Prabhakaran D, Clifford GD, Maulik PK, et al. A multifaceted strategy using mobile technology to assist rural primary healthcare doctors and frontline health workers in cardiovascular disease risk management: protocol for the SMARTHealth India cluster randomised controlled trial. Implement Sci. 2013;8:137.

37. Ward J, McGregor S, Guy RJ, Rumbold AR, Garton L, Silver BJ, et al. STI in remote communities: improved and enhanced primary health care (STRIVE) study protocol: a cluster randomised controlled trial comparing 'usual practice' STI care to enhanced care in remote primary health care services in Australia. BMC Infect Dis. 2013;13:425.

38. Bennett PN, Daly RM, Fraser SF, Haines T, Barnard R, Ockerby C, et al. The impact of an exercise physiologist coordinated resistance exercise program on the physical function of people receiving hemodialysis: a stepped wedge randomised control study. BMC Nephrol. 2013;14:204.

39. Poldervaart JM, Reitsma JB, Koffijberg H, Backus BE, Six AJ, Doevendans PA, et al. The impact of the HEART risk score in the early assessment of patients with acute chest pain: design of a stepped wedge, cluster randomised trial. BMC Cardiovasc Disord. 2013;13:77.

40. Rasmussen CD, Holtermann A, Mortensen OS, Sogaard K, Jorgensen MB. Prevention of low back pain and its consequences among nurses' aides in elderly care: a stepped-wedge multi-faceted cluster-randomized controlled trial. BMC Public Health. 2013;13:1088.

41. van Holland BJ, de Boer MR, Brouwer S, Soer R, Reneman MF. Sustained employability of workers in a production environment: design of a stepped wedge trial to evaluate effectiveness and cost-benefit of the POSE program. BMC Public Health. 2012;12:1003.

42. Dreischulte T, Grant A, Donnan P, McCowan C, Davey P, Petrie D, et al. A cluster randomised stepped wedge trial to evaluate the effectiveness of a multifaceted information technology-based intervention in reducing high-risk prescribing of non-steroidal anti-inflammatory drugs and antiplatelets in primary medical care: the DQIP study protocol. Implementation science : IS. 2012;7:24.

43. Flocke SA, Antognoli E, Step MM, Marsh S, Parran T, Mason MJ. A Teachable Moment Communication Process for smoking cessation talk: description of a group randomized clinician-focused intervention. BMC health services research. 2012;12:109.

44. Marshall T, Caley M, Hemming K, Gill P, Gale N, Jolly K. Mixed methods evaluation of targeted case finding for cardiovascular disease prevention using a stepped wedged cluster RCT. BMC public health. 2012;12:908.

45. Mouchoux C, Rippert P, Duclos A, Fassier T, Bonnefoy M, Comte B, et al. Impact of a multifaceted program to prevent postoperative delirium in the elderly: the CONFUCIUS stepped wedge protocol. BMC geriatrics. 2011;11:25.

46. Turner J, Kelly B, Clarke D, Yates P, Aranda S, Jolley D, et al. A randomised trial of a psychosocial intervention for cancer patients integrated into routine care: the PROMPT study (promoting optimal outcomes in mood through tailored psychosocial therapies). BMC Cancer. 2011;11:48.

47. Liddy C, Hogg W, Russell G, Wells G, Armstrong CD, Akbari A, et al. Improved delivery of cardiovascular care (IDOCC) through outreach facilitation: study protocol and implementation details of a cluster randomized controlled trial in primary care. Implement Sci. 2011;6:110.

48. The Gambia Hepatitis Intervention Study. The Gambia Hepatitis Study Group. Cancer Research. 1987;47(21):5782-7.

49. Schultz TJ, Kitson AL, Soenen S, Long L, Shanks A, Wiechula R, et al. Does a multidisciplinary nutritional intervention prevent nutritional decline in hospital patients? A stepped wedge randomised cluster trial. e-SPEN Journal. 2014;9(2):e84-e90.

For peer review only

50. Kjeken I, Berdal G, Bo I, Dager T, Dingsor A, Hagfors J, et al. Evaluation of a structured goal planning and tailored follow-up programme in rehabilitation for patients with rheumatic diseases: protocol for a pragmatic, stepped-wedge cluster randomized trial. BMC musculoskeletal disorders. 2014;15:153.

51. Stringer JS, Chisembele-Taylor A, Chibwesha CJ, Chi HF, Ayles H, Manda H, et al. Protocol-driven primary care and community linkages to improve population health in rural Zambia: the Better Health Outcomes through Mentoring and Assessment (BHOMA) project. BMC Health Serv Res. 2013;13 Suppl 2:S7.

52. De Allegri M, Pokhrel S, Becher H, Dong H, Mansmann U, Kouyate B, et al. Step-wedge cluster-randomised community-based trials: an application to the study of the impact of community health insurance. Health research policy and systems / BioMed Central. 2008;6:10.

53. Gucciardi E, Fortugno M, Horodezny S, Lou W, Sidani S, Espin S, et al. Will Mobile Diabetes Education Teams (MDETs) in primary care improve patient care processes and health outcomes? Study protocol for a randomized controlled trial. Trials. 2012;13:165.

54. Williams AL, Phillips CJ, Watkins A, Rushton AB. The effect of work-based mentoring on patient outcome in musculoskeletal physiotherapy: study protocol for a randomised controlled trial. Trials. 2014;15:409.

55. Mosha F, Winani S, Wood S, Changalucha J, Ngasalla B. Evaluation of the effectiveness of a clean delivery kit intervention in preventing cord infection and puerperal sepsis among neonates and their mothers in rural Mwanza Region, Tanzania. Tanzania health research bulletin. 2005;7(3):185-8.

56. Strijbos MJ, Steunenberg B, van der Mast RC, Inouye SK, Schuurmans MJ. Design and methods of the Hospital Elder Life Program (HELP), a multicomponent targeted intervention to prevent delirium in hospitalized older patients: efficacy and cost-effectiveness in Dutch health care. BMC Geriatr. 2013;13:78.

57. Zwijsen SA, Smalbrugge M, Eefsting JA, Twisk JWR, Gerritsen DL, Pot AM, et al. Coming to grips with challenging behavior: A cluster randomized controlled trial on the effects of a multidisciplinary care program for challenging behavior in Dementia. Journal of the American Medical Directors Association. 2014;15(7):531.e1-.e10.

58. Baker C, Huxley P, Dennis M, Islam S, Russell I. Alleviating staff stress in care homes for people with dementia: Protocol for stepped-wedge cluster randomised trial to evaluate a web-based mindfulness- stress reduction course. BMC Psychiatry 2015 12;15.

59. Baker M, Biringen Z, Meyer-Parsons B, Schneider A. Emotional attachment and emotional availability tele-intervention for adoptive families. Infant Ment Health J 2015;36(2):179-192.

60. Banga FR, Truijens SEM, Fransen AF, Dieleman JP, Van Runnard Heimel PJ, Oei GS. The impact of transmural multiprofessional simulation-based obstetric team training on perinatal outcome and quality of care in the Netherlands. BMC Med Educ 2014;14(1).

61. Brady MC, Stott D, Weir CJ, Chalmers C, Sweeney P, Donaldson C, et al. Clinical and cost effectiveness of enhanced oral healthcare in stroke care settings (SOCLE II): A pilot, stepped wedge, cluster randomized, controlled trial protocol. Int J Stroke 2015;10(6):979-984.

62. Britton B, McCarter K, Baker A, Wolfenden L, Wratten C, Bauer J, et al. Eating As Treatment (EAT) study protocol: A stepped-wedge, randomised controlled trial of a health behaviour change intervention provided by dietitians to improve nutrition in patients with head and neck cancer undergoing radiotherapy. BMJ Open 2015;5(7).

63. Chavane L, Merialdi M, Betrán AP, Requejo-Harris J, Bergel E, Aleman A, et al. Implementation of evidence-based antenatal care in Mozambique: A cluster randomized controlled trial: Study protocol. BMC Health Serv Res 2014;14(1).

64. Cowan JF, Micek M, Cowan JFG, Napua M, Hoek R, Gimbel S, et al. Early ART initiation among HIV-positive pregnant women in central Mozambique: a stepped wedge randomized controlled trial of an optimized Option B+ approach. Implementation Science 2015;10:61.

65. Dryden-Peterson S, Bennett K, Hughes MD, Veres A, John O, Pradhananga R, et al. An augmented SMS intervention to improve access to antenatal CD4 testing and ART initiation in HIV-infected pregnant women: A cluster randomized trial. PLoS ONE 2015;10(2).

66. Durovni B, Saraceni V, van den Hof S, Trajman A, Cordeiro-Santos M, Cavalcante S, et al. Impact of Replacing Smear Microscopy with Xpert MTB/RIF for Diagnosing Tuberculosis in Brazil: A Stepped-Wedge Cluster-Randomized Trial. PLoS Med 2014;11(12).

67. Fiscella K, Ogedegbe G, He H, Carroll J, Cassells A, Sanders M, et al. Blood Pressure Visit Intensification Study in Treatment: Trial design. Am Heart J 2015;170(6):1202-1210.

68. Golden MR, Kerani RP, Stenger M, Hughes JP, Aubin M, Malinski C, et al. Uptake and Population-Level Impact of Expedited Partner Therapy (EPT) on Chlamydia trachomatis and Neisseria gonorrhoeae: The Washington State Community-Level Randomized Trial of EPT. PLoS Med 2015;12(1):1-22.

69. Grace AM, Lippert S, Collins K, Pineda N, Tolani A, Walker R, et al. Educating health care professionals on human trafficking. Pediatr Emerg Care 2014;30(12):856-861.

70. Haines TP, O'Brien L, Mitchell D, Bowles K-, Haas R, Markham D, et al. Study protocol for two randomized controlled trials examining the effectiveness and safety of current weekend allied health services and a new stakeholder-driven model for acute medical/surgical patients versus no weekend allied health services. Trials 2015;16(1).

71. Happ MB, Sereika SM, Houze MP, Seaman JB, Tate JA, Nilsen ML, et al. Quality of care and resource use among mechanically ventilated patients before and after an intervention to assist nurse-nonvocal patient communication. Heart Lung J Acute Crit Care 2015;44(5):408-415.e2.

72. Hayden MK, Lin MY, Lolans K, Weiner S, Blom D, Moore NM, et al. Prevention of colonization and infection by klebsiella pneumoniae carbapenemase-producing enterobacteriaceae in long-term acute-care hospitals. Clin Infect Dis 2015;60(8):1154-1161.

73. Helitzer DL, Hathorn G, Benally J, Ortega C. Culturally relevant model program to prevent and reduce agricultural injuries. J Agric Saf Health 2014;20(3):175-198.

74. Hill A-, McPhail SM, Waldron N, Etherton-Beer C, Ingram K, Flicker L, et al. Fall rates in hospital rehabilitation units after individualised patient and staff education programmes: A pragmatic, stepped-wedge, cluster-randomised controlled trial. Lancet 2015;385(9987):2592-2599.

75. Hunter SB, Ober AJ, Paddock SM, Hunt PE, Levan D. Continuous quality improvement (CQI) in addiction treatment settings: design and intervention protocol of a group randomized pilot study. Addict Sci Clin Pract 2014;9:4.

76. Jarvik JG, Comstock BA, James KT, Avins AL, Bresnahan BW, Deyo RA, et al. Lumbar Imaging With Reporting Of Epidemiology (LIRE)-Protocol for a pragmatic cluster randomized trial. Contemp Clin Trials 2015;45:157-163.

77. Jordan S, Gabe-Walters ME, Watkins A, Humphreys I, Newson L, Snelgrove S, et al. Nurse-led medicines' monitoring for patients with dementia in care homes: A pragmatic cohort stepped wedge cluster randomised trial. PLoS ONE 2015;10(10).

78. Kelly PJ, Baker AL, Deane FP, Callister R, Collins CE, Oldmeadow C, et al. Study protocol: a stepped wedge cluster randomised controlled trial of a healthy lifestyle intervention for people attending residential substance abuse treatment. BMC Public Health 2015;15:465.

79. Li S, Wu Y, Du X, Li X, Patel A, Peterson ED, et al. Rational and design of a stepped-wedge cluster randomized trial evaluating quality improvement initiative for reducing cardiovascular events among patients with acute coronary syndromes in resource-constrained hospitals in China. Am Heart J 2015;169(3):349-355.

80. Marrin K, Wood F, Firth J, Kinsey K, Edwards A, Brain KE, et al. Option Grids to facilitate shared decision making for patients with Osteoarthritis of the knee: Protocol for a single site, efficacy trial. BMC Health Serv Res 2014;14(1).

81. Morrison LJ, Brooks SC, Dainty KN, Dorian P, Needham DM, Ferguson ND, et al. Improving use of targeted temperature management after out-of-hospital cardiac arrest: A stepped wedge cluster randomized controlled trial. Crit Care Med 2015;43(5):954-964.

82. Noel PH, Romero RL, Robertson M, Parchman ML. Key activities used by community based primary care practices to improve the quality of diabetes care in response to practice facilitation. Quality in Primary Care 2014;22(4):211-219.

For peer review only

83. Nosyk B, Krebs E, Min JE, Ahamad K, Buxton J, Goldsmith C, et al. The 'Expanded HIV care in opioid substitution treatment' (EHOST) cluster-randomized, stepped-wedge trial: A study protocol. Contemp Clin Trials 2015;45:201-209.

84. Olds DL, Baca P, McClatchey M, Ingoldsby EM, Luckey DW, Knudtson MD, et al. Cluster randomized controlled trial of intervention to increase participant retention and completed home visits in the Nurse-Family Partnership. Prevention Science 2015 08;16(6):778-788.

85. Palmer VJ, Chondros P, Piper D, Callander R, Weavell W, Godbee K, et al. The CORE study protocol: A stepped wedge cluster randomised controlled trial to test a co-design technique to optimise psychosocial recovery outcomes for people affected by mental illness in the community mental health setting. BMJ Open 2015;5(3).

86. Pickering BW, Dong Y, Ahmed A, Giri J, Kilickaya O, Gupta A, et al. The implementation of clinician designed, human-centered electronic medical record viewer in the intensive care unit: A pilot step-wedge cluster randomized trial. Int J Med Informatics 2016;84(5):299-307.

87. Ralph AP, Read C, Johnston V, de Dassel JL, Bycroft K, Mitchell A, et al. Improving delivery of secondary prophylaxis for rheumatic heart disease in remote Indigenous communities: Study protocol for a stepped-wedge randomised trial. Trials 2016;17(1).

88. Rodriguez V, Giuffre C, Villa S, Almada G, Prasopa-Plaizier N, Gogna M, et al. A multimodal intervention to improve hand hygiene in ICUs in Buenos Aires, Argentina: A stepped wedge trial. Int J Qual Health Care 2015;27(5):405-411.

89. Shah L, Rojas M, Mori O, Zamudio C, Kaufman JS, Otero L, et al. Implementation of a stepped-wedge cluster randomized design in routine public health practice: design and application for a tuberculosis (TB) household contact study in a high burden area of Lima, Peru. BMC Public Health 2015;15:587.

90. Suman A, Schaafsma FG, Elders PJM, van Tulder MW, Anema JR. Cost-effectiveness of a multifaceted implementation strategy for the Dutch multidisciplinary guideline for nonspecific low back pain: design of a stepped-wedge cluster randomised controlled trial. BMC Public Health 2015;15:522.

91. Tielsch JM, Katz J, Zeger SL, Khatry SK, Shrestha L, Breysse P, et al. Designs of two randomized, community-based trials to assess the impact of alternative cookstove installation on respiratory illness among young children and reproductive outcomes in rural Nepal. BMC Public Health 2014;14:1271.

92. Tiono AB, Pinder M, N'Fale S, Faragher B, Smith T, Silkey M, et al. The AvecNet Trial to assess whether addition of pyriproxyfen, an insect juvenile hormone mimic, to long-lasting insecticidal mosquito nets provides additional protection against clinical malaria over current best practice in an area with pyrethroid-resistant vectors in rural Burkina Faso: Study protocol for a randomised controlled trial. Trials 2015;16(1).

93. Toftegaard BS, Bro F, Vedsted P. A geographical cluster randomised stepped wedge study of continuing medical education and cancer diagnosis in general practice. Implementation Science 2014;9:159.

94. van Daalen FV, Prins JM, Opmeer BC, Boermeester MA, Visser CE, van Hest RM, et al. A cluster randomized trial for the implementation of an antibiotic checklist based on validated quality indicators: The AB-checklist. BMC Infect Dis 2015;15(1).

95. Var C, Bazzano AN, Srivastav SK, Welty JC, Ek NI, Oberhelman RA. Newborn Infection Control and Care Initiative for health facilities to accelerate reduction of newborn mortality (NICCI): Study protocol for a randomized controlled trial. Trials 2015;16(1).

96. Verberne CJ, Zhan Z, Van Den Heuvel E, Grossmann I, Doornbos PM, Havenga K, et al. Intensified follow-up in colorectal cancer patients using frequent Carcino-Embryonic Antigen (CEA) measurements and CEA-triggered imaging: Results of the randomized "CEAwatch" trial. Eur J Surg Oncol 2015;41(9):1188-1196.

97. Wald HL, Bandle B, Richard AA, Min S, Capezuti E. A Trial of electronic surveillance feedback for quality improvement at Nurses Improving Care for Healthsystem Elders (NICHE) hospitals. Am J Infect Control 2014 Oct;42(10 Suppl):S250-6.

98. Williamson A, Redman S, Haynes A, Barker D, Jorm L, Green S, et al. Supporting Policy In health with Research: an Intervention Trial (SPIRIT)—protocol for a stepped wedge trial. BMJ Open 2014;4(7).

For peer review only

99. Wu M-, Wu C-, Chen B-. Behavioral Intervention and Decreased Daily Melamine Exposure from Melamine Tableware. Environ Sci Technol 2015;49(16):9964-9970.

100. Hoogendijk EO, van der Horst HE, van de Ven PM, Twisk JWR, Deeg DJH, Frijters DHM, et al. Effectiveness of a Geriatric Care Model for frail older adults in primary care: Results from a stepped wedge cluster randomised trial. Eur J Intern Med 2015;28(43).

101. Ononge S, Campbell OMR, Kaharuza F, Lewis JL, Fielding K, Mirembe F. Effectiveness and safety of misoprostol distributed to antenatal women to prevent postpartum haemorrhage after child-births: a stepped-wedge cluster-randomised trial. BMC Pregnancy and Childbirth 2015;15(315).

For peer review only

PRISMA 2009 Checklist

Section/topic	#	Checklist item	Reported on page #
**TITLE**			
Title	1	Identify the report as a systematic review, meta-analysis, or both.	1-2
**ABSTRACT**			
Structured summary	2	Provide a structured summary including, as applicable: background; objectives; data sources; study eligibility criteria, participants, and interventions; study appraisal and synthesis methods; results; limitations; conclusions and implications of key findings; systematic review registration number.	12-35
**INTRODUCTION**			
Rationale	3	Describe the rationale for the review in the context of what is already known.	50-111
Objectives	4	Provide an explicit statement of questions being addressed with reference to participants, interventions, comparisons, outcomes, and study design (PICOS).	111-114
**METHODS**			
Protocol and registration	5	Indicate if a review protocol exists, if and where it can be accessed (e.g., Web address), and, if available, provide registration information including registration number.	NA
Eligibility criteria	6	Specify study characteristics (e.g., PICOS, length of follow-up) and report characteristics (e.g., years considered, language, publication status) used as criteria for eligibility, giving rationale.	117-129
Information sources	7	Describe all information sources (e.g., databases with dates of coverage, contact with study authors to identify additional studies) in the search and date last searched.	117-121
Search	8	Present full electronic search strategy for at least one database, including any limits used, such that it could be repeated.	Supplementary File 1
Study selection	9	State the process for selecting studies (i.e., screening, eligibility, included in systematic review, and, if applicable, included in the meta-analysis).	130-134
Data collection process	10	Describe method of data extraction from reports (e.g., piloted forms, independently, in duplicate) and any processes for obtaining and confirming data from investigators.	136-139
Data items	11	List and define all variables for which data were sought (e.g., PICOS, funding sources) and any assumptions and simplifications made.	140-166
Risk of bias in individual studies	12	Describe methods used for assessing risk of bias of individual studies (including specification of whether this was done at the study or outcome level), and how this information is to be used in any data synthesis.	NA
Summary measures	13	State the principal summary measures (e.g., risk ratio, difference in means).	140-166
Synthesis of results	14	Describe the methods of handling data and combining results of studies, if done, including measures of consistency (e.g., I^2) for each meta-analysis.	167-172

For peer review only - http://bmjopen.bmj.com/site/about/guidelines.xhtml

PRISMA 2009 Checklist

Section/topic	#	Checklist item	Reported on page #
Risk of bias across studies	15	Specify any assessment of risk of bias that may affect the cumulative evidence (e.g., publication bias, selective reporting within studies).	NA
Additional analyses	16	Describe methods of additional analyses (e.g., sensitivity or subgroup analyses, meta-regression), if done, indicating which were pre-specified.	NA
**RESULTS**			
Study selection	17	Give numbers of studies screened, assessed for eligibility, and included in the review, with reasons for exclusions at each stage, ideally with a flow diagram.	174-178, Figure 2
Study characteristics	18	For each study, present characteristics for which data were extracted (e.g., study size, PICOS, follow-up period) and provide the citations.	179-260, Table 1
Risk of bias within studies	19	Present data on risk of bias of each study and, if available, any outcome level assessment (see item 12).	NA
Results of individual studies	20	For all outcomes considered (benefits or harms), present, for each study: (a) simple summary data for each intervention group (b) effect estimates and confidence intervals, ideally with a forest plot.	NA
Synthesis of results	21	Present results of each meta-analysis done, including confidence intervals and measures of consistency.	NA
Risk of bias across studies	22	Present results of any assessment of risk of bias across studies (see Item 15).	NA
Additional analysis	23	Give results of additional analyses, if done (e.g., sensitivity or subgroup analyses, meta-regression [see Item 16]).	NA
**DISCUSSION**			
Summary of evidence	24	Summarize the main findings including the strength of evidence for each main outcome; consider their relevance to key groups (e.g., healthcare providers, users, and policy makers).	262-312
Limitations	25	Discuss limitations at study and outcome level (e.g., risk of bias), and at review-level (e.g., incomplete retrieval of identified research, reporting bias).	331-345
Conclusions	26	Provide a general interpretation of the results in the context of other evidence, and implications for future research.	346-375
**FUNDING**			
Funding	27	Describe sources of funding for the systematic review and other support (e.g., supply of data); role of funders for the systematic review.	376-384

From: Moher D, Liberati A, Tetzlaff J, Altman DG, The PRISMA Group (2009). Preferred Reporting Items for Systematic Reviews and Meta-Analyses: The PRISMA Statement. PLoS Med 6(7): e1000097. doi:10.1371/journal.pmed1000097

For more information, visit: www.prisma-statement.org.

For peer review only - http://bmjopen.bmj.com/site/about/guidelines.xhtml

BMJ Open

Unequal cluster sizes in stepped-wedge cluster randomised trials: a systematic review

Journal:	*BMJ Open*
Manuscript ID	bmjopen-2017-017151.R2
Article Type:	Research
Date Submitted by the Author:	13-Sep-2017
Complete List of Authors:	Kristunas, Caroline; University of Leicester, Department of Health Sciences<br>Morris, Tom; University of Leicester, Leicester Clinical Trials Unit<br>Gray, Laura; University of Leicester, Department of Health Sciences
**Primary Subject Heading**:	Research methods
Secondary Subject Heading:	Public health
Keywords:	Stepped-wedge trial, Systematic review, Cluster randomised trial

SCHOLARONE™
Manuscripts

For peer review only

Unequal cluster sizes in stepped-wedge cluster randomised trials: a systematic review

Caroline Kristunas[1*], Tom Morris[2], Laura J Gray[1]

Authors Affiliations:

[1]Department of Health Sciences, University of Leicester, Leicester, UK
[2]Leicester Clinical Trials Unit, University of Leicester, Leicester, UK

**Corresponding author:* Caroline Kristunas; cak21@le.ac.uk

Keywords: Stepped-wedge trial; systematic review; cluster randomised trial.

For peer review only

ABSTRACT

Objectives: To investigate the extent to which cluster sizes vary in stepped-wedge cluster randomised trials (SW-CRTs) and whether any variability is accounted for during the sample size calculation and analysis of these trials.

Setting: Any, not limited to healthcare settings.

Participants: Any taking part in a SW-CRT published up to March 2016.

Primary and secondary outcome measures: The primary outcome is the variability in cluster sizes, measured by the coefficient of variation in cluster size (CV). Secondary outcomes include the difference between the cluster sizes assumed during the sample size calculation and those observed during the trial, any reported variability in cluster sizes and whether the methods of sample size calculation and methods of analysis accounted for any variability in cluster sizes.

Results: Of the 101 included SW-CRTs, 48% mentioned that the included clusters were known to vary in size, yet only 13% of these accounted for this during the calculation of the sample size. However, 69% of the trials did use a method of analysis appropriate for when clusters vary in size. Full trial reports were available for 53 trials. The CV was calculated for 23 of these: the median CV was 0.41 (interquartile range: 0.22-0.52). Actual cluster sizes could be compared to those assumed during the sample size calculation for 14 (26%) of the trial reports; the cluster sizes were between 29% and 480% of that which had been assumed.

Conclusions: Cluster sizes often vary in SW-CRTs. Reporting of SW-CRTs also remains sub-optimal. The effect of unequal cluster sizes on the statistical power of SW-CRTs needs further exploration and methods appropriate to studies with unequal cluster sizes need to be employed.

STRENGTHS AND LIMITATIONS OF THIS STUDY:

- To our knowledge this is the first systematic review to assess how often and to what extent cluster sizes are unequal in stepped-wedge cluster randomised trials (SW-CRTs) and determine whether the current methodology being used accounts for any variability in cluster sizes.
- This review used pre-specified search and study selection strategies as well as using double data extraction.
- Due to poor reporting quality, the actual cluster sizes could not be extracted for all studies, and cluster sizes were therefore estimated from whatever information was provided.

For peer review only

BACKGROUND

Cluster randomised trials (CRTs) are often used to evaluate healthcare interventions that are implemented at the cluster level, for example, within hospitals, general practices or across geographical areas, where treatment contamination would be likely to occur if individual randomisation was used.[1] In conventional parallel CRTs, clusters are generally randomised to either a control or intervention group (usually with equal numbers in each group). After baseline measurements have been taken, those randomised to the intervention group are exposed to the intervention for the remainder of the trial, whereas those in the control group are not.

The stepped-wedge CRT (SW-CRT) is a relatively recent variant of the CRT. In SW-CRTs the clusters are randomised to start the intervention at different time points, known as steps, rather than being randomised to either a control or an intervention group. All of the clusters generally start the trial in a control group, then switch to the intervention when their time comes, until by the end of the trial all of the clusters are exposed to the intervention.[2] The cluster size is the number of individuals providing outcome measurements, either per cluster per measurement point, or per cluster over the whole duration of the trial. Figure 1 gives a schematic representation of a SW-CRT. Although use of this trial design is still relatively rare, recent systematic reviews have shown there to be a dramatic rise in the number of SW-CRTs published within the last few years.[3] However, the methodological advances for SW-CRTs lag behind those for other trial designs.

Due to the variability that occurs in the natural sizes of some clusters, the actual observed cluster sizes often vary within a CRT.[1] For both cohort and cross-sectional designs the actual observed cluster size can be defined as either the number of observations actually accrued over the whole duration of the trial or the number of observations actually accrued at each measurement point, with the latter enabling the former to be calculated. Clusters may also vary in size due to differences in the rates of loss to follow-up and incidence rates across clusters.[1] Increasing variability in cluster sizes has been shown to cause a loss of statistical power in parallel CRTs,[4] particularly if the coefficient of variation in cluster size (CV) (given as the standard deviation of the cluster size divided by the mean cluster size) is greater than 0.23.[5] This can lead to a trial that is under-powered to detect the effect of the intervention that is being evaluated. Various sample size calculation methods have thus been developed for parallel CRTs which account for any imbalance in cluster sizes, to ensure that the trial will be sufficiently powered.[5-8] However, although the effect of unequal cluster sizes has been reported for parallel CRTs, it has only been reported for cross-sectional SW-CRTs with a continuous outcome and analysed using generalised estimating equations (GEEs).[9] It is expected that some loss of power will be observed for certain sub-types of SW-CRTs and therefore a similar adjustment to the sample size calculation is likely to be required.

It has been recommended that mixed-effects models (MMs) or GEEs should be used to analyse SW-CRTs.[10] In particular, linear MMs can be used for continuous data, plausibly Normally distributed, or when cluster sizes are approximately equal, but if cluster sizes vary and the outcome is non-normal, then generalised linear MM (GLMM) or

For peer review only

GEEs are preferred.[10] This is because both of these methods can account for the variability in cluster sizes, as well as non-normal data.[10] It is therefore to be expected that MMs and GEEs will be employed in SW-CRTs where the cluster sizes are likely to vary. Other methods of analysis that can also account for the design features of SW-CRTs (such as clustering and potential time effects) may also be suitable for use when cluster sizes vary, however, the appropriateness of these methods for the analysis of SW-CRTs with variable cluster sizes is currently unknown. To date, MMs and GEEs are the only methods of analysis for which the efficiency of the method has been investigated when cluster sizes vary.

Varying cluster sizes should also be considered during the randomisation process, as larger clusters may have different characteristics to smaller clusters. If more than one cluster switches to the intervention at each time point, then the randomisation process should be such that it ensures that cluster sizes are balanced across time points.

A recent systematic review of SW-CRTs found that few studies reported any variation in cluster sizes. However, major deficiencies were found in the reporting [11] and so this is likely to be an underestimation of the true extent of variability in cluster sizes. The size of the variability in cluster sizes in SW-CRTs has not previously been reported, nor how any imbalance in cluster sizes is being adjusted for during the calculation of the sample size or the analysis of these trials. The primary aims of our review were therefore to:

- Determine how often and to what extent cluster sizes are unequal in SW-CRTs.
- Determine how unequal cluster sizes are being taken into account during the calculation of the sample size and the analysis of SW-CRTs.

METHODS

Search strategy

We included in our review studies identified by a recent systematic review conducted by Martin et al of SW-CRTs up to 23 October 2014.[11] In addition, we also included studies published more recently, by searching MEDLINE, Scopus and EBSCO Host, up to 21 March 2016, using an adapted version of the Martin et al search strategy (adapted to each database and to exclude studies prior to 23 October 2014). The search strategy is provided in Supplementary File 1. The studies that were included were from protocols or independent full reports of SW-CRTs in both healthcare and non-healthcare settings.[11] The studies had to be randomised trials, using cluster randomisation, and have at least two steps. We did not restrict our search to trials where all clusters started in the control condition or ended in the intervention condition. Trials that were not published in English, were individually or non-randomised trials, trials with a cross-over design and those that were retrospectively analysed as a SW-CRT were excluded.[11] We focused on original research studies and primary study reports.

Once duplicates had been removed, the titles and abstracts of the studies identified by the search were screened for eligibility by CK. Full-text articles were then obtained for all potentially eligible studies and screened independently by two authors (CK and TM). Those studies found not to meet the eligibility criteria were excluded and the reasons for exclusion tabulated. Any differences of opinion were resolved by discussions with LG.

For peer review only

Data extraction

Data for all studies meeting the eligibility criteria were extracted by CK and checked by TM. Any differences of opinion were resolved through discussions with all authors. A data extraction form was developed, then tested and refined on a small number of studies.

Basic trial characteristics are reported. These include the year of publication, type of cluster, the type of primary outcome (binary, continuous, time-to-event etc.), number of clusters required and whether any restrictions related to cluster sizes were used in the randomisation procedure.

Information was also extracted in relation to variability in cluster sizes. We report whether it was mentioned in the report or protocol that clusters were known to vary in size prior to the trial commencing, whether any variability in cluster sizes was accounted for during the randomisation process or calculation of the sample size, and whether the method of analysis appropriately accounts for this variability. We also recorded the number and sizes of the clusters assumed during the sample size calculation. For completed trials we extracted the actual cluster sizes and reported the variability in cluster sizes that was observed during the trial, and made a comparison of the actual cluster sizes with the cluster sizes that were assumed during the sample size calculations.

Reporting of SW-CRTs is poor [11, 12] and so to increase the number of trials available for a comparison of cluster sizes, some cluster sizes had to be estimated from whatever information was available. Cluster sizes per time period, at a specific time point (such as at baseline) or the total cluster sizes over the full duration of the trial were preferred. If this information was not available then other information relating to the sizes of the clusters was used to estimate the cluster sizes, so that an estimate of the variability in the sizes of the clusters could be made. This included using the reported number of eligible individuals in each cluster as an approximation of the number of individuals included in each cluster, assuming that all clusters in a randomisation group (clusters randomised to switch to the intervention at the same time) were of equal size when only the mean cluster size per randomisation group was given, or using the mean and standard deviation of cluster size in each randomisation group to find the CV for each group. The comparison of cluster sizes is reported separately for the actual cluster sizes and those that were estimated from summary measures.

Analysis

We summarise the basic trial characteristics, methods of sample size calculation and methods of analysis, and number and size of clusters assumed during the sample size calculation for study reports and protocols. Actual cluster sizes, estimated actual cluster sizes and comparisons of actual cluster sizes with those that were assumed during the calculation of the sample size are summarised for trial reports only.

RESULTS

We identified 371 records through our search of electronic databases. After combining this with the records identified by Martin et al,[11] removing three protocols for which the full reports had been identified, and after removing duplicates, we were left with 330

For peer review only

records. We screened these records and excluded 229 which did not meet our eligibility criteria (Figure 2). A full list of the 101 included studies is given in Supplementary File 2.

The general characteristics of the included trials are presented in Table 1. Full trial reports were available for 53 (52%) trials, with only protocols available for the remaining 48 (48%) trials. Two (2%) trials were conducted as pilot studies. A primary outcome was reported for 87 (86%) trials, of which the majority were binary (45%), time-to-event and rate (17%) or continuous (16%) outcomes and six (7%) reported multiple primary outcomes. Three (3%) trials did not report a primary outcome and for the remaining 11 (11%) trials the primary outcome was not clear. For the majority of the trials (52%), the clusters were comprised of health care facilities, such as hospitals, hospital wards, clinics or GP practices. The remaining clusters comprised of geographical areas/communities (10%), residential/long-term care facilities (9%), health care professionals (9%), educational facilities (6%), rehabilitation clinics/programs (5%) family groups/households (4%), or other groups (5%).

Table 1: Trial characteristics of included SW-CRTs. Unless stated the denominator is the number of included studies (n=101).

**Characteristic**	**Number**	**Percentage**
Included studies	101	
Publication type**:**		
Report	53	52.5
Protocol	48	47.5
Publication describes results from a pilot study	2	2.0
Primary outcome reported	87	86.1
Primary outcome type (where reported, denominator=87):		
Binary	39	44.8
Time-to-event and rate	15	17.2
Continuous	14	16.1
Count	7	8.0
Ordinal	6	6.9
Multiple	6	6.9
Types of cluster:		
Health care facilities	53	52.5
Geographical areas/communities	10	9.9
Residential/long-term care facilities	9	8.9
Health care professionals (including groups thereof)	9	8.9
Education facilities	6	5.9
Rehabilitation clinics/programs	5	5.0
Family groups/households	4	4.0
Other	5	5.0
Evidence of clusters being known to vary in size	48	47.5
Sample size calculation presented	82	81.2
Accounted for unequal cluster sizes in sample size calculation	6	5.9

For peer review only

Method used to account for unequal cluster sizes in sample size calculation (where reported, denominator=6):		
Used coefficient of variation in cluster size	2	33.3
Accounted for in simulation	2	33.3
Unclear	2	33.3
Reported number of clusters required	71	70.3
Median (interquartile range) number of clusters required (n=71)	11 (6 – 22.5)	
Reported required cluster size	57	56.4
Median (interquartile range) per time period (n=23)	30 (20 - 125)	
Median (interquartile range) overall (n=34)	51 (20.75 – 165)	
Reported total sample size required[1]	64	63.4
Accounted for unequal cluster sizes in the randomisation	29	28.7
Method of analysis reported	94	93.1
Method of analysis used (where reported, denominator=94):		
GEEs	13	12.9
MM	57	56.4
GLMM	32	31.7
LMM	7	6.9
Unclear	18	17.8
Other models	16	15.8
Generalised LM	5	5.0
GLM (accounting for clustering)	5	5.0
LM/ANCOVA	6	5.9
Simple analysis[2]	8	7.9
Unclear	5	5.0
Not given	2	2.0

[1] *Total sample size required either stated or enough detail given for it to be reproduced.*
[2] *Including - McNemar's, Mann-Whitney U, t-test, ANOVA, bivariate, Wilcoxon signed-rank*

For just under half (48, 48%) of the trials there was evidence that the clusters were known to vary in size. Yet few of these accounted for inequality in cluster size during the randomisation process (29%) or sample size calculation (13%). Nine stratified by cluster size, one pair-matched according to cluster size, two restricted randomisation by cluster size, one used minimisation to balance cluster sizes and one used block randomisation by cluster size. One additional trial used stratification, but did not state for which variables stratification was applied.

Sample size calculation

Only 82 (81%) of the included studies presented a sample size calculation, of which 71 reported the number of clusters required and 57 reported the required cluster sizes (23 reported cluster size per time period, 34 reported total cluster size). The median (interquartile range (IQR)) number of clusters required for these trials was 11 (6 – 22.5). The median (IQR) cluster size was 30 (20 - 125) for those that gave the cluster size per

For peer review only

time period and 51 (20.75-165) for those that gave the total cluster size over the duration of the trial.

In total only six (6%) trials reported that they had accounted for unequal cluster sizes when calculating the required sample size. Two used an estimate of the coefficient of variation in cluster size, two used simulations to calculate the required sample size, varying the cluster sizes in their simulations, and for the remaining two trials the method used to account for unequal cluster sizes was unclear.

Methods of analysis

A small proportion of the trials (12%) attempted to adjust for any inequality in cluster size through the inclusion of covariates in the analysis and one trial planned to conduct an individual level analysis, rather than cluster level analysis, if clusters were found to vary in size.

The majority of analyses were conducted using mixed-effects models (MM) (56%), with 32 studies using generalised linear MMs, seven using linear MMs and 18 not distinguishing between the two. An equal number of trials used generalised estimating equations (13%) as used simple regression models (linear or generalised linear models) (13%). Only five of those that used simple regression models accounted for the clustering of the data during the analysis. The method of analysis for the primary outcome was unclear for five studies and two studies did not report in their protocol details of how the analysis would be conducted. The remaining 11 (11%) studies employed simple analysis methods, such as ANOVA and two sample t-tests, which did not account for the clustering of the data nor any period effect. The majority of studies (69%) employed a method of analysis known to be appropriate for when cluster sizes vary in SW-CRTs (LMM, GLMM or GEEs). Of the 48 studies for which there was evidence that cluster sizes were known to vary, 75.0% (36) of those used LMM, GLMM or GEEs in the analysis. Whereas, for those 23 trials where the cluster sizes were actually found to vary in size only 12 (52.2%) of them used LMM, GLMM or GEEs.

Reporting of actual cluster sizes

Of the 53 included full trial reports, it was only possible to determine the actual cluster sizes for 13 (25%) of the trials. Five of these trials gave the cluster sizes during each time period, four gave the size of each cluster at a specific time point, such as at baseline or the point of analysis and the remaining four reports gave the total size of each cluster over the duration of the whole trial. One further trial gave the number of eligible individuals in each cluster. A further eight (15%) reports gave a summary measure of the cluster sizes, such as the mean per month, per randomisation group, per intervention group etc. or the range of cluster sizes. Two (4%) reports gave the sizes of the randomisation groups only.

Comparison of cluster sizes

Few study reports presented both the required cluster sizes and the actual cluster sizes. The way in which cluster sizes were presented varied between studies and some only presented summary measures of the cluster sizes. Of the 24 trial reports that presented some indication of the cluster sizes, only 14 (58%) also stated the required cluster size. For those trials that presented the actual cluster sizes, the individual cluster sizes, either over the whole trial or at each time point, were between 29% and 480% of the required

For peer review only

cluster size (median=100%, IQR=90%, 100%). For those trials that reported a summary measure of the actual cluster sizes, the cluster sizes were between 30% and 209% of the required cluster size (median=79%, IQR=65%, 101%). The average cluster size for each trial ranged between 66% and 214% of the required cluster size (median=95%, IQR=79%, 116%).

None of the trial reports presented the actual value of the coefficient of variation (CV) in cluster size. It was possible to estimate the CV in cluster size for 23 of the trials, of which only two experienced no variability in cluster size. The median CV in cluster size was 0.41 (IQR: 0.22-0.52) and the maximum value was 1.29. . None of these trials reported that they accounted for unequal cluster sizes during the sample size calculation.

DISCUSSION

We conducted a systematic review to assess how often and to what extent cluster sizes are unequal in SW-CRTs, and to determine how any inequality in cluster sizes is currently being taken into account during the calculation of the sample sizes and the analysis of these trials. Of the 101 SW-CRTs included, almost half (48%) mentioned that the clusters were known to vary in size, yet many of these did not account for this during the calculation of the sample size. Simple analytical methods of sample size calculation which account for any variability in cluster sizes exist for parallel CRTs.[5-8] No such methods have been developed for use with SW-CRTs, though it may be possible to adapt the methods for parallel CRTs for use in SW-CRTs.

About 69% of the trials did however employ a method of analysis deemed appropriate for when cluster sizes are unequal in SW-CRTs (LMM, GLMM or GEEs).[10] There may be other methods of analysis that are also suitable for SW-CRTs with unequal cluster sizes, but these have yet to be investigated. In addition to the suitability of the analysis method to the variability in cluster sizes in a SW-CRT, the appropriateness of the method to the SW-CRT design must also be considered. Clustering and time effects will also need to be considered as well. Barker et al [13] and Davey et al [14] have both conducted reviews that consider the suitability of analysis methods for SW-CRTs.

Full trial reports were available for 53 trials. Only a quarter of these reported the sizes of each cluster, either per time period, at a specific point during the trial, or the total size over the whole duration of the trial. The CV in cluster size was calculated based on this information. A further eight trials reported a summary measure of the cluster sizes, such as the mean cluster size per month or the mean cluster size per randomisation group. From these summary measures we were able to produce an estimate of the CV in cluster size for each of these trials. One further trial only gave the number of eligible individuals in each cluster and so, assuming equal recruitment rates in each cluster, it was also possible to estimate the CV in cluster size for this trial. Better reporting of CV is required for SW-CRTs, whether this be the CV across all periods or the CV per period.

Only two trials experienced no variability in cluster size. The majority (70%) of trials had a CV in cluster size >0.23 and so if these had been parallel CRTs we would have expected these trials to have suffered a significant loss of statistical power.[5] However, a recent simulation study has shown that for cross-sectional SW-CRTs with a continuous outcome

For peer review only

and intracluster correlation coefficient (ICC) of 0.05, even high variability in cluster sizes does not cause a significant loss of power.[9] Nevertheless, a recent systematic review found that the majority of SW-CRTs are not of a cross-sectional design [12] and within health research an ICC of 0.05 is generally considered high. It is for these cohort, low ICC, and other trials that the effect of unequal cluster sizes is still unknown. However, even if unequal cluster sizes are found to cause a reduction in power for most SW-CRTs, this effect may be overshadowed by the effect of using an incorrect sample size calculation, such as one that does not allow for the effect of time. Not allowing for the effect of time in this calculation can result in both under- and over-powered studies and so even with unequal cluster sizes, a study might still be over-powered.[11] It is therefore important that the sample size calculation for a SW-CRT correctly allows for all aspects of the design.

The actual cluster sizes also varied considerably from those that were assumed during the calculation of the required sample size, with reported cluster sizes being between 29% and 480% of that which was required. This would suggest that some of the trials we examined are likely to have been considerably over- or under-powered. However, half of the studies that presented the actual cluster sizes did use clusters within 10% of the required size and half of those that presented a summary measure of the actual cluster size used clusters within 22% of the actual size. These comparisons could only be made for 14 (26%) of the trials with published reports. Information both on the sizes of the clusters that were assumed during the calculation of the sample size and the actual cluster sizes that were observed during the trial were often not reported.

The majority of systematic reviews of SW-CRTs have been conducted with the aim of assessing the quality and breadth of SW-CRTs.[3, 15, 16] A couple of recent systematic reviews have also been conducted to assess the quality of reporting and methodological rigor of SW-CRTs,[11, 12] as well as the statistical methodology being used and available for these trials.[13] However, none of these systematic reviews have assessed the prevalence and severity of unequal cluster sizes in SW-CRTs, nor the statistical methodology being used to account for this variability in cluster sizes. Our review has highlighted the high degree of variability in cluster sizes that is observed in SW-CRTs. Knowing that cluster sizes often vary in SW-CRTs can allow researchers to plan for an expected inequality in cluster sizes, taking this into account when calculating the required sample size for these trials and choosing an appropriate method of analysis. We also highlight the need for improved quality of reporting of cluster sizes in SW-CRTs, which concurs with the findings of previous reviews [11, 12] and the need for wider implementation of methodologies which account for unequal cluster sizes. These findings are in line with what has been shown more generally in CRTs.[17]

Our study has several strengths. In order to minimise the potential for bias during the review, we pre-specified search and study selection strategies. We also did not limit our search to publications from journals with a high impact factor, increasing the generalisability of our findings. However, our study does have limitations as well. For example, selection bias may have been introduced by the choice to only include studies published in English. It was also difficult to ensure the identification of all SW-CRTs, as many do not include in either their title or abstract the common terms that identify a SW-CRT. We included in our search strategy all of the common identifiers that have been

For peer review only

included in previous reviews, yet this is still not sufficient to identify studies where the researchers weren't aware that their studies were SW-CRTs and therefore did not include common identifiers in their title or abstract. This may introduce some selection bias. The poor reporting of SW-CRTs [11, 12] may limit the generalisability of the findings of our study. It was only possible to make a comparison of cluster sizes and to calculate the CV in cluster size for a small number of studies, and these are likely to be those studies that have a better quality of reporting than the other studies. The estimations that were made when determining the observed cluster sizes may also result in an under- or over-estimation of the degree of variability in cluster sizes and the comparability of the observed cluster sizes with those which were required.

Unequal cluster sizes is a common problem in SW-CRTs. Variability in cluster sizes is not being accounted for during the calculation of the sample size for these trials and the reporting of the actual cluster sizes observed during these trials is poor, which limits the scope of this review. Appropriate methods of sample size calculation that account for variability in cluster sizes should be developed by methodological statisticians and perhaps implemented in appropriate software in order to allow their widespread use. Simulation methods, such as those used by Baio et al, [18] could be used whilst simple analytical methods are developed. Further research is also required to investigate the effect of unequal cluster sizes on the statistical power for cohort SW-CRTs and those with a binary outcome. An investigation into how our results differ between cohort and cross-sectional designs would also be of interest.

We echo the views of others,[2] that the development of reporting guidelines for SW-CRTs is needed, particularly to improve the sub-optimal quality of reporting for the sample size calculations and of the actual observed cluster sizes in SW-CRTs. In the meantime it is recommended that reporting should follow the CONSORT 2010 extension to cluster randomised trials [19] and the modifications presented by Hemming et al in their paper.[2] A CONSORT extension to stepped-wedge cluster randomised trials is currently in development.[20]

CONCLUSIONS

Cluster sizes were found to vary in SW-CRTs. The effect of unequal cluster sizes on SW-CRTs is yet to be reported for cohort designs and those with a binary outcome and so the effect of this observed variability is currently unknown. Methods of sample size calculation that are used for these trials rarely account for any variability in cluster size. Reporting of cluster sizes in both the reported sample size calculation and those actually observed during the trial is poor. Authors need to provide greater detail of how the required sample size has been calculated as well as providing details of the actual cluster sizes observed during the trial, preferably at each step of the trial. Researchers should also be aware that cluster sizes are likely to vary in SW-CRTs and so sample size calculation methods and analysis methods should be used which can account for this variability.

FUNDING

For peer review only

CK is funded by a National Institute for Health Research (NIHR) Research Methods Fellowship. The authors would also like to acknowledge support from the NIHR Collaboration for Leadership in Applied Health Research and Care – East Midlands (NIHR CLAHRC – EM), Leicester Clinical Trials Unit and the NIHR Leicester-Loughborough Diet, Lifestyle and Physical Activity Biomedical Research Unit, which is a partnership between University Hospitals of Leicester NHS Trust, Loughborough University and the University of Leicester. The views expressed are those of the authors and not necessarily those of the NIHR or the Department of Health.

COMPETING INTERESTS

None declared.

DATA SHARING

No additional data are available.

CONTRIBUTORS

LJG conceptualised the review. CK carried out the systematic review, designed the data extraction form, identified the studies for inclusion, extracted data, performed the data analysis and wrote the first draft. TM carried out the second data extraction and critically reviewed the contents of the paper. LJG resolved any differences between CK and TM in the data extraction. All authors contributed to the writing of the paper, read and approved the final manuscript.

REFERENCES

1 Eldridge S, Kerry S. A Practical Guide to Cluster Randomised Trials in Health Services Research. US: John Wiley & Sons Inc 2012.

2 Hemming K, Haines TP, Chilton PJ, et al. The stepped wedge cluster randomised trial: rationale, design, analysis, and reporting, *BMJ* 2015;350:h391 doi:10.1136/bmj.h391.

3 Beard E, Lewis J, Copas A, et al. Stepped wedge randomised controlled trials: systematic review of studies published between 2010 and 2014, *Trials* 2015;16:1-14.

4 Lauer SA, Kleinman KP, Reich NG. The Effect of Cluster Size Variability on Statistical Power in Cluster-Randomized Trials, *PLoS ONE* 2015;10:e0119074.

5 Eldridge SM, Ashby D, Kerry S. Sample size for cluster randomized trials: effect of coefficient of variation of cluster size and analysis method, *Int J Epidemiol* 2006;35:1292-300.

6 Kerry SM, Bland JM. Unequal cluster sizes for trials in English and Welsh general practice: implications for sample size calculations, *Stat Med* 2001;20:377-90 doi:AID-SIM799>3.0.CO;2-N.

For peer review only

7 Manatunga AK, Hudgens MG, Chen S. Sample Size Estimation in Cluster Randomized Studies with Varying Cluster Size, *Biometrical Journal* 2001;43:75-86.

8 Pan W. Sample Size and Power Calculations with Correlated Binary Data, *Control Clin Trials* 2001;22:211-27.

9 Kristunas CA, Smith KL, Gray LJ. An imbalance in cluster sizes does not lead to notable loss of power in cross-sectional, stepped-wedge cluster randomised trials with a continuous outcome, *Trials* 2017;18:109.

10 Hussey MA, Hughes JP. Design and analysis of stepped wedge cluster randomized trials, *Contemporary Clinical Trials* 2007;28:182-91.

11 Martin J, Taljaard M, Girling A, et al. Systematic review finds major deficiencies in sample size methodology and reporting for stepped-wedge cluster randomised trials, *BMJ open* 2016;6:e010166 doi:10.1136/bmjopen-2015-010166.

12 Grayling MJ, Wason JM, Mander AP. Stepped wedge cluster randomized controlled trial designs: a review of reporting quality and design features, *Trials* 2017;18:33.

13 Barker D, McElduff P, D'Este C, et al. Stepped wedge cluster randomised trials: A review of the statistical methodology used and available, *BMC Medical Research Methodology* 2016;16 doi:10.1186/s12874-016-0176-5.

14 Davey C, Hargreaves J, Thompson JA, et al. Analysis and reporting of stepped wedge randomised controlled trials: Synthesis and critical appraisal of published studies, 2010 to 2014, *Trials* 2015;16.

15 Brown C, Lilford R. The stepped wedge trial design: a systematic review, *BMC Medical Research Methodology* 2006;6:54.

16 Mdege ND, Man M, Taylor (nee Brown), Celia A, et al. Systematic review of stepped wedge cluster randomized trials shows that design is particularly used to evaluate interventions during routine implementation, *J Clin Epidemiol* 2011;64:936-48.

17 Rutterford C, Taljaard M, Dixon S, et al. Reporting and methodological quality of sample size calculations in cluster randomized trials could be improved: a review, *J Clin Epidemiol* 2015;68:716-23.

18 Baio G, Copas A, Ambler G, et al. Sample size calculation for a stepped wedge trial, *Trials* 2015;16:354.

19 Campbell MK, Piaggio G, Elbourne DR, et al. Consort 2010 statement: Extension to cluster randomised trials, *BMJ (Online)* 2012;345.

20 Hemming K, Girling A, Haines T, et al. Protocol: Consort extension to stepped wedge cluster randomised controlled trial, *BMJ* 2014.

For peer review only

FIGURE LEGENDS

Figure 1: Schematic representation of a stepped-wedge cluster randomised trial with four steps. The white areas represent control periods and the shaded areas represent intervention periods.

Figure 2: Flow chart showing the studies identified by the systematic review.

For peer review only

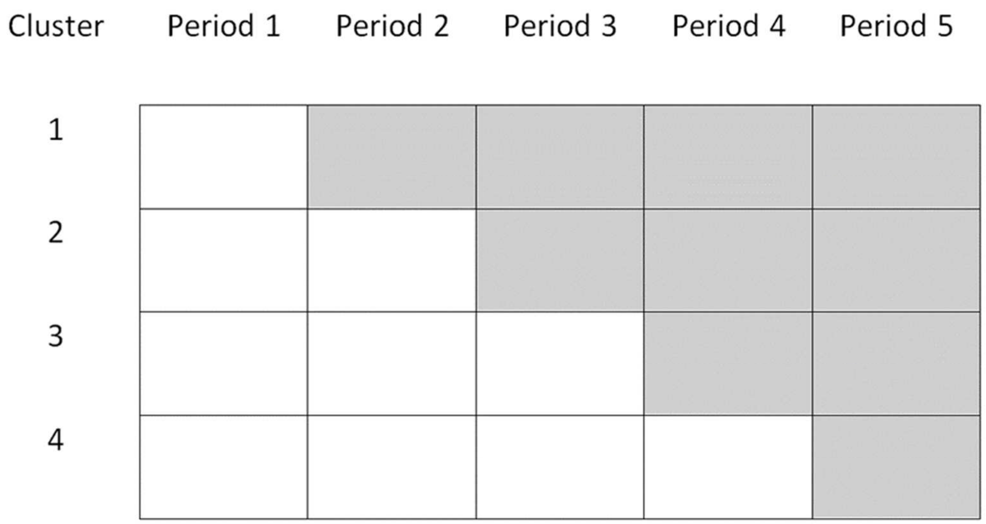

Schematic representation of a stepped-wedge cluster randomised trial with four steps. The white areas represent control periods and the shaded areas represent intervention periods.

190x107mm (300 x 300 DPI)

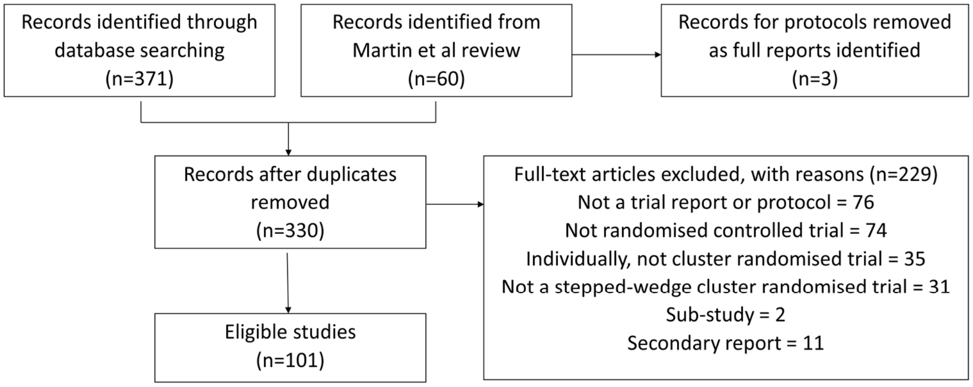

Flow chart showing the studies identified by the systematic review.

190x107mm (300 x 300 DPI)

review only

SYSTEMATIC REVIEW SEARCH STRATEGY

1. step$ wedge.ti,ab.
2. experimentally staged introduction.ti,ab.
3. delayed intervention.ti,ab.
4. (one directional cross over design or one directional crossover design).ti,ab.
5. ((incremental or phased or staggered or stepwise or step wise or delayed) adj1 (recruitment or introduction or implementation)).ti,ab.
6. or/1-5
7. limit 6 to english language
8. limit 7 to randomized controlled trial
9. limit 8 to humans
10. limit 9 to yr=”2014-current”

For peer review only

Reference list of included studies

1. Craine N, Whitaker R, Perrett S, Zou L, Hickman M, Lyons M. A stepped wedge cluster randomized control trial of dried blood spot testing to improve the uptake of hepatitis C antibody testing within UK prisons. European journal of public health. 2014.

2. Palmay L, Elligsen M, Walker SAN, Pinto R, Walker S, Einarson T, et al. Hospital-wide rollout of antimicrobial stewardship: A stepped-wedge randomized trial. Clinical Infectious Diseases. 2014;59(6):867-74.

3. van de Steeg L, R IJ, Langelaan M, Wagner C. Can an e-learning course improve nursing care for older people at risk of delirium: a stepped wedge cluster randomised trial. BMC geriatrics. 2014;14:69.

4. Haugen AS, Softeland E, Almeland SK, Sevdalis N, Vonen B, Eide GE, et al. Effect of the World Health Organization Checklist on Patient Outcomes: A Stepped Wedge Cluster Randomized Controlled Trial. Annals of surgery. 2014.

5. Stern A, Mitsakakis N, Paulden M, Alibhai S, Wong J, Tomlinson G, et al. Pressure ulcer multidisciplinary teams via telemedicine: a pragmatic cluster randomized stepped wedge trial in long term care. BMC Health Serv Res. 2014;14:83.

6. Enns E, Rhemtulla R, Ewa V, Fruetel K, Holroyd-Leduc JM. A controlled quality improvement trial to reduce the use of physical restraints in older hospitalized adults. Journal of the American Geriatrics Society. 2014;62(3):541-5.

7. Roy A, Anaraki S, Hardelid P, Catchpole M, Rodrigues LC, Lipman M, et al. Universal HIV testing in London tuberculosis clinics: A cluster randomised controlled trial. European Respiratory Journal. 2013;41(3):627-34.

8. Mhurchu CN, Gorton D, Turley M, Jiang Y, Michie J, Maddison R, et al. Effects of a free school breakfast programme on children's attendance, academic achievement and short-term hunger: Results from a stepped-wedge, cluster randomised controlled trial. Journal of Epidemiology and Community Health. 2013;67(3):257-64.

9. Leontjevas R, Gerritsen DL, Smalbrugge M, Teerenstra S, Vernooij-Dassen MJFJ, Koopmans RTCM. A structural multidisciplinary approach to depression management in nursing-home residents: A multicentre, stepped-wedge cluster-randomised trial. The Lancet. 2013;381(9885):2255-64.

10. Gruber JS, Reygadas F, Arnold BF, Ray I, Nelson K, Colford Jr JM. A stepped wedge, cluster-randomized trial of a household UV-disinfection and safe storage drinking water intervention in rural Baja California Sur, Mexico. American Journal of Tropical Medicine and Hygiene. 2013;89(2):238-45.

11. Durovni B, Saraceni V, Moulton LH, Pacheco AG, Cavalcante SC, King BS, et al. Effect of improved tuberculosis screening and isoniazid preventive therapy on incidence of tuberculosis and death in patients with HIV in clinics in Rio de Janeiro, Brazil: A stepped wedge, cluster-randomised trial. The Lancet Infectious Diseases. 2013;13(10):852-8.

12. Bashour HN, Kanaan M, Kharouf MH, Abdulsalam AA, Tabbaa MA, Cheikha SA. The effect of training doctors in communication skills on women's satisfaction with doctor-woman relationship during labour and delivery: A stepped wedge cluster randomised trial in Damascus. BMJ Open. 2013;3(8).

13. van den Broek IV, van Bergen JE, Brouwers EE, Fennema JS, Gotz HM, Hoebe CJ, et al. Effectiveness of yearly, register based screening for chlamydia in the Netherlands: Controlled trial with randomised stepped wedge implementation. BMJ: British Medical Journal. 2012;345(7869):1-12.

14. Monse B, Duijster D, Sheiham A, Grijalva-Eternod CS, van Palenstein Helderman W, Hobdell MH. The effects of extraction of pulpally involved primary teeth on weight, height and BMI in underweight Filipino children. A cluster randomized clinical trial. BMC public health. 2012;12:725.

15. Horner C, Wilcox M, Barr B, Hall D, Hodgson G, Parnell P, et al. The longitudinal prevalence of MRSA in care home residents and the effectiveness of improving infection prevention knowledge and practice on colonisation using a stepped wedge study design. BMJ Open. 2012;2(1):e000423.

16. Fuller C, Michie S, Savage J, McAteer J, Besser S, Charlett A, et al. The Feedback Intervention Trial (FIT)--improving hand-hygiene compliance in UK healthcare workers: a stepped wedge cluster randomised controlled trial. PloS one. 2012;7(10):e41617.

For peer review only

17. Chinbuah MA, Kager PA, Abbey M, Gyapong M, Awini E, Nonvignon J, et al. Impact of community management of fever (using antimalarials with or without antibiotics) on childhood mortality: A cluster-randomized controlled trial in Ghana. American Journal of Tropical Medicine and Hygiene. 2012;87(SUPPL.5):11-20.

18. Etchells E, Adhikari NK, Wu R, Cheung M, Quan S, Mraz R, et al. Real-time automated paging and decision support for critical laboratory abnormalities. BMJ quality & safety. 2011;20(11):924-30.

19. Bacchieri G, Barros AJ, Santos JV, Goncalves H, Gigante DP. A community intervention to prevent traffic accidents among bicycle commuters. Revista de saude publica. 2010;44(5):867-75.

20. Solomon E, Rees T, Ukoumunne OC, Metcalf B, Hillsdon M. The Devon Active Villages Evaluation (DAVE) trial of a community-level physical activity intervention in rural south-west England: A stepped wedge cluster randomised controlled trial. The International Journal of Behavioral Nutrition and Physical Activity. 2014;11:94.

21. Gozalo P, Prakash S, Qato DM, Sloane PD, Mor V. Effect of the bathing without a battle training intervention on bathing-associated physical and verbal outcomes in nursing home residents with dementia: A randomized crossover diffusion study. Journal of the American Geriatrics Society. 2014;62(5):797-804.

22. Foster JM, Hoskins G, Smith B, Lee AJ, Price D, Pinnock H. Practice development plans to improve the primary care management of acute asthma: randomised controlled trial. BMC Fam Pract. 2007;8:23.

23. Flannery DJ, Vazsonyi AT, Liau AK, Guo S, Powell KE, Atha H, et al. Initial behavior outcomes for the peacebuilders universal school-based violence prevention program. Developmental psychology. 2003;39(2):292-308.

24. Priestley G, Watson W, Rashidian A, Mozley C, Russell D, Wilson J, et al. Introducing Critical Care Outreach: A ward-randomised trial of phased introduction in a general hospital. Intensive Care Medicine. 2004;30(7):1398-404.

25. Husaini BA, Reece MC, Emerson JS, Scales S, Hull PC, Levine RS. A church-based program on prostate cancer screening for African American men: reducing health disparities. Ethn Dis. 2008;18(2 Suppl 2):S2-179-84.

26. Viera AJ, Garrett JM. Preliminary study of a school-based program to improve hypertension awareness in the community. Family medicine. 2008;40(4):264-70.

27. Barton A, Basham M, Foy C, Buckingham K, Somervill M. The Watcombe Housing Study: the short term effect of improving housing conditions on the health of residents. J Epidemiol Community Health. 2007;61(9):771-7.

28. Bailet LL, Repper KK, Piasta SB, Murphy SP. Emergent literacy intervention for prekindergarteners at risk for reading failure. Journal of Learning Disabilities. 2009;42(4):336-55.

29. Weiner M, El Hoyek G, Wang L, Dexter PR, Zerr AD, Perkins AJ, et al. A web-based generalist-specialist system to improve scheduling of outpatient specialty consultations in an academic center. Journal of general internal medicine. 2009;24(6):710-5.

30. Brown BB, Young J, Smith DP, Kneebone AB, Brooks AJ, Xhilaga M, et al. Clinician-led improvement in cancer care (CLICC)--testing a multifaceted implementation strategy to increase evidence-based prostate cancer care: phased randomised controlled trial--study protocol. Implementation science : IS. 2014;9:64.

31. Reuther S, Holle D, Buscher I, Dortmann O, Muller R, Bartholomeyczik S, et al. Effect evaluation of two types of dementia-specific case conferences in German nursing homes (FallDem) using a stepped-wedge design: Study protocol for a randomized controlled trial. Trials. 2014;15(1).

32. Bernabe-Ortiz A, Diez-Canseco F, Gilman RH, Cardenas MK, Sacksteder KA, Miranda JJ. Launching a salt substitute to reduce blood pressure at the population level: A cluster randomized stepped wedge trial in Peru. Trials. 2014;15(1).

33. Brimblecombe J, Ferguson M, Liberato SC, Ball K, Moodie ML, Magnus A, et al. Stores Healthy Options Project in Remote Indigenous Communities (SHOP@RIC): a protocol of a randomised trial promoting healthy food and beverage purchases through price discounts and in-store nutrition education. BMC Public Health. 2013;13:744.

For peer review only

34. Feng R, Li K, Cheng J, Xie S, Chai J, Wei P, et al. Toward integrated and sustainable prevention against diabetes in rural China: Study rationale and protocol of eCROPS. BMC Endocrine Disorders. 2013;13(28).

35. Keriel-Gascou M, Buchet-Poyau K, Duclos A, Rabilloud M, Figon S, Dubois JP, et al. Evaluation of an interactive program for preventing adverse drug events in primary care: study protocol of the InPAct cluster randomised stepped wedge trial. Implementation science : IS. 2013;8:69.

36. Praveen D, Patel A, McMahon S, Prabhakaran D, Clifford GD, Maulik PK, et al. A multifaceted strategy using mobile technology to assist rural primary healthcare doctors and frontline health workers in cardiovascular disease risk management: protocol for the SMARTHealth India cluster randomised controlled trial. Implement Sci. 2013;8:137.

37. Ward J, McGregor S, Guy RJ, Rumbold AR, Garton L, Silver BJ, et al. STI in remote communities: improved and enhanced primary health care (STRIVE) study protocol: a cluster randomised controlled trial comparing 'usual practice' STI care to enhanced care in remote primary health care services in Australia. BMC Infect Dis. 2013;13:425.

38. Bennett PN, Daly RM, Fraser SF, Haines T, Barnard R, Ockerby C, et al. The impact of an exercise physiologist coordinated resistance exercise program on the physical function of people receiving hemodialysis: a stepped wedge randomised control study. BMC Nephrol. 2013;14:204.

39. Poldervaart JM, Reitsma JB, Koffijberg H, Backus BE, Six AJ, Doevendans PA, et al. The impact of the HEART risk score in the early assessment of patients with acute chest pain: design of a stepped wedge, cluster randomised trial. BMC Cardiovasc Disord. 2013;13:77.

40. Rasmussen CD, Holtermann A, Mortensen OS, Sogaard K, Jorgensen MB. Prevention of low back pain and its consequences among nurses' aides in elderly care: a stepped-wedge multi-faceted cluster-randomized controlled trial. BMC Public Health. 2013;13:1088.

41. van Holland BJ, de Boer MR, Brouwer S, Soer R, Reneman MF. Sustained employability of workers in a production environment: design of a stepped wedge trial to evaluate effectiveness and cost-benefit of the POSE program. BMC Public Health. 2012;12:1003.

42. Dreischulte T, Grant A, Donnan P, McCowan C, Davey P, Petrie D, et al. A cluster randomised stepped wedge trial to evaluate the effectiveness of a multifaceted information technology-based intervention in reducing high-risk prescribing of non-steroidal anti-inflammatory drugs and antiplatelets in primary medical care: the DQIP study protocol. Implementation science : IS. 2012;7:24.

43. Flocke SA, Antognoli E, Step MM, Marsh S, Parran T, Mason MJ. A Teachable Moment Communication Process for smoking cessation talk: description of a group randomized clinician-focused intervention. BMC health services research. 2012;12:109.

44. Marshall T, Caley M, Hemming K, Gill P, Gale N, Jolly K. Mixed methods evaluation of targeted case finding for cardiovascular disease prevention using a stepped wedged cluster RCT. BMC public health. 2012;12:908.

45. Mouchoux C, Rippert P, Duclos A, Fassier T, Bonnefoy M, Comte B, et al. Impact of a multifaceted program to prevent postoperative delirium in the elderly: the CONFUCIUS stepped wedge protocol. BMC geriatrics. 2011;11:25.

46. Turner J, Kelly B, Clarke D, Yates P, Aranda S, Jolley D, et al. A randomised trial of a psychosocial intervention for cancer patients integrated into routine care: the PROMPT study (promoting optimal outcomes in mood through tailored psychosocial therapies). BMC Cancer. 2011;11:48.

47. Liddy C, Hogg W, Russell G, Wells G, Armstrong CD, Akbari A, et al. Improved delivery of cardiovascular care (IDOCC) through outreach facilitation: study protocol and implementation details of a cluster randomized controlled trial in primary care. Implement Sci. 2011;6:110.

48. The Gambia Hepatitis Intervention Study. The Gambia Hepatitis Study Group. Cancer Research. 1987;47(21):5782-7.

49. Schultz TJ, Kitson AL, Soenen S, Long L, Shanks A, Wiechula R, et al. Does a multidisciplinary nutritional intervention prevent nutritional decline in hospital patients? A stepped wedge randomised cluster trial. e-SPEN Journal. 2014;9(2):e84-e90.

For peer review only

50. Kjeken I, Berdal G, Bo I, Dager T, Dingsor A, Hagfors J, et al. Evaluation of a structured goal planning and tailored follow-up programme in rehabilitation for patients with rheumatic diseases: protocol for a pragmatic, stepped-wedge cluster randomized trial. BMC musculoskeletal disorders. 2014;15:153.

51. Stringer JS, Chisembele-Taylor A, Chibwesha CJ, Chi HF, Ayles H, Manda H, et al. Protocol-driven primary care and community linkages to improve population health in rural Zambia: the Better Health Outcomes through Mentoring and Assessment (BHOMA) project. BMC Health Serv Res. 2013;13 Suppl 2:S7.

52. De Allegri M, Pokhrel S, Becher H, Dong H, Mansmann U, Kouyate B, et al. Step-wedge cluster-randomised community-based trials: an application to the study of the impact of community health insurance. Health research policy and systems / BioMed Central. 2008;6:10.

53. Gucciardi E, Fortugno M, Horodezny S, Lou W, Sidani S, Espin S, et al. Will Mobile Diabetes Education Teams (MDETs) in primary care improve patient care processes and health outcomes? Study protocol for a randomized controlled trial. Trials. 2012;13:165.

54. Williams AL, Phillips CJ, Watkins A, Rushton AB. The effect of work-based mentoring on patient outcome in musculoskeletal physiotherapy: study protocol for a randomised controlled trial. Trials. 2014;15:409.

55. Mosha F, Winani S, Wood S, Changalucha J, Ngasalla B. Evaluation of the effectiveness of a clean delivery kit intervention in preventing cord infection and puerperal sepsis among neonates and their mothers in rural Mwanza Region, Tanzania. Tanzania health research bulletin. 2005;7(3):185-8.

56. Strijbos MJ, Steunenberg B, van der Mast RC, Inouye SK, Schuurmans MJ. Design and methods of the Hospital Elder Life Program (HELP), a multicomponent targeted intervention to prevent delirium in hospitalized older patients: efficacy and cost-effectiveness in Dutch health care. BMC Geriatr. 2013;13:78.

57. Zwijsen SA, Smalbrugge M, Eefsting JA, Twisk JWR, Gerritsen DL, Pot AM, et al. Coming to grips with challenging behavior: A cluster randomized controlled trial on the effects of a multidisciplinary care program for challenging behavior in Dementia. Journal of the American Medical Directors Association. 2014;15(7):531.e1-.e10.

58. Baker C, Huxley P, Dennis M, Islam S, Russell I. Alleviating staff stress in care homes for people with dementia: Protocol for stepped-wedge cluster randomised trial to evaluate a web-based mindfulness- stress reduction course. BMC Psychiatry 2015 12;15.

59. Baker M, Biringen Z, Meyer-Parsons B, Schneider A. Emotional attachment and emotional availability tele-intervention for adoptive families. Infant Ment Health J 2015;36(2):179-192.

60. Banga FR, Truijens SEM, Fransen AF, Dieleman JP, Van Runnard Heimel PJ, Oei GS. The impact of transmural multiprofessional simulation-based obstetric team training on perinatal outcome and quality of care in the Netherlands. BMC Med Educ 2014;14(1).

61. Brady MC, Stott D, Weir CJ, Chalmers C, Sweeney P, Donaldson C, et al. Clinical and cost effectiveness of enhanced oral healthcare in stroke care settings (SOCLE II): A pilot, stepped wedge, cluster randomized, controlled trial protocol. Int J Stroke 2015;10(6):979-984.

62. Britton B, McCarter K, Baker A, Wolfenden L, Wratten C, Bauer J, et al. Eating As Treatment (EAT) study protocol: A stepped-wedge, randomised controlled trial of a health behaviour change intervention provided by dietitians to improve nutrition in patients with head and neck cancer undergoing radiotherapy. BMJ Open 2015;5(7).

63. Chavane L, Merialdi M, Betrán AP, Requejo-Harris J, Bergel E, Aleman A, et al. Implementation of evidence-based antenatal care in Mozambique: A cluster randomized controlled trial: Study protocol. BMC Health Serv Res 2014;14(1).

64. Cowan JF, Micek M, Cowan JFG, Napua M, Hoek R, Gimbel S, et al. Early ART initiation among HIV-positive pregnant women in central Mozambique: a stepped wedge randomized controlled trial of an optimized Option B+ approach. Implementation Science 2015;10:61.

65. Dryden-Peterson S, Bennett K, Hughes MD, Veres A, John O, Pradhananga R, et al. An augmented SMS intervention to improve access to antenatal CD4 testing and ART initiation in HIV-infected pregnant women: A cluster randomized trial. PLoS ONE 2015;10(2).

For peer review only

66. Durovni B, Saraceni V, van den Hof S, Trajman A, Cordeiro-Santos M, Cavalcante S, et al. Impact of Replacing Smear Microscopy with Xpert MTB/RIF for Diagnosing Tuberculosis in Brazil: A Stepped-Wedge Cluster-Randomized Trial. PLoS Med 2014;11(12).

67. Fiscella K, Ogedegbe G, He H, Carroll J, Cassells A, Sanders M, et al. Blood Pressure Visit Intensification Study in Treatment: Trial design. Am Heart J 2015;170(6):1202-1210.

68. Golden MR, Kerani RP, Stenger M, Hughes JP, Aubin M, Malinski C, et al. Uptake and Population-Level Impact of Expedited Partner Therapy (EPT) on Chlamydia trachomatis and Neisseria gonorrhoeae: The Washington State Community-Level Randomized Trial of EPT. PLoS Med 2015;12(1):1-22.

69. Grace AM, Lippert S, Collins K, Pineda N, Tolani A, Walker R, et al. Educating health care professionals on human trafficking. Pediatr Emerg Care 2014;30(12):856-861.

70. Haines TP, O'Brien L, Mitchell D, Bowles K-, Haas R, Markham D, et al. Study protocol for two randomized controlled trials examining the effectiveness and safety of current weekend allied health services and a new stakeholder-driven model for acute medical/surgical patients versus no weekend allied health services. Trials 2015;16(1).

71. Happ MB, Sereika SM, Houze MP, Seaman JB, Tate JA, Nilsen ML, et al. Quality of care and resource use among mechanically ventilated patients before and after an intervention to assist nurse-nonvocal patient communication. Heart Lung J Acute Crit Care 2015;44(5):408-415.e2.

72. Hayden MK, Lin MY, Lolans K, Weiner S, Blom D, Moore NM, et al. Prevention of colonization and infection by klebsiella pneumoniae carbapenemase-producing enterobacteriaceae in long-term acute-care hospitals. Clin Infect Dis 2015;60(8):1154-1161.

73. Helitzer DL, Hathorn G, Benally J, Ortega C. Culturally relevant model program to prevent and reduce agricultural injuries. J Agric Saf Health 2014;20(3):175-198.

74. Hill A-, McPhail SM, Waldron N, Etherton-Beer C, Ingram K, Flicker L, et al. Fall rates in hospital rehabilitation units after individualised patient and staff education programmes: A pragmatic, stepped-wedge, cluster-randomised controlled trial. Lancet 2015;385(9987):2592-2599.

75. Hunter SB, Ober AJ, Paddock SM, Hunt PE, Levan D. Continuous quality improvement (CQI) in addiction treatment settings: design and intervention protocol of a group randomized pilot study. Addict Sci Clin Pract 2014;9:4.

76. Jarvik JG, Comstock BA, James KT, Avins AL, Bresnahan BW, Deyo RA, et al. Lumbar Imaging With Reporting Of Epidemiology (LIRE)-Protocol for a pragmatic cluster randomized trial. Contemp Clin Trials 2015;45:157-163.

77. Jordan S, Gabe-Walters ME, Watkins A, Humphreys I, Newson L, Snelgrove S, et al. Nurse-led medicines' monitoring for patients with dementia in care homes: A pragmatic cohort stepped wedge cluster randomised trial. PLoS ONE 2015;10(10).

78. Kelly PJ, Baker AL, Deane FP, Callister R, Collins CE, Oldmeadow C, et al. Study protocol: a stepped wedge cluster randomised controlled trial of a healthy lifestyle intervention for people attending residential substance abuse treatment. BMC Public Health 2015;15:465.

79. Li S, Wu Y, Du X, Li X, Patel A, Peterson ED, et al. Rational and design of a stepped-wedge cluster randomized trial evaluating quality improvement initiative for reducing cardiovascular events among patients with acute coronary syndromes in resource-constrained hospitals in China. Am Heart J 2015;169(3):349-355.

80. Marrin K, Wood F, Firth J, Kinsey K, Edwards A, Brain KE, et al. Option Grids to facilitate shared decision making for patients with Osteoarthritis of the knee: Protocol for a single site, efficacy trial. BMC Health Serv Res 2014;14(1).

81. Morrison LJ, Brooks SC, Dainty KN, Dorian P, Needham DM, Ferguson ND, et al. Improving use of targeted temperature management after out-of-hospital cardiac arrest: A stepped wedge cluster randomized controlled trial. Crit Care Med 2015;43(5):954-964.

82. Noel PH, Romero RL, Robertson M, Parchman ML. Key activities used by community based primary care practices to improve the quality of diabetes care in response to practice facilitation. Quality in Primary Care 2014;22(4):211-219.

For peer review only

83. Nosyk B, Krebs E, Min JE, Ahamad K, Buxton J, Goldsmith C, et al. The 'Expanded HIV care in opioid substitution treatment' (EHOST) cluster-randomized, stepped-wedge trial: A study protocol. Contemp Clin Trials 2015;45:201-209.

84. Olds DL, Baca P, McClatchey M, Ingoldsby EM, Luckey DW, Knudtson MD, et al. Cluster randomized controlled trial of intervention to increase participant retention and completed home visits in the Nurse-Family Partnership. Prevention Science 2015 08;16(6):778-788.

85. Palmer VJ, Chondros P, Piper D, Callander R, Weavell W, Godbee K, et al. The CORE study protocol: A stepped wedge cluster randomised controlled trial to test a co-design technique to optimise psychosocial recovery outcomes for people affected by mental illness in the community mental health setting. BMJ Open 2015;5(3).

86. Pickering BW, Dong Y, Ahmed A, Giri J, Kilickaya O, Gupta A, et al. The implementation of clinician designed, human-centered electronic medical record viewer in the intensive care unit: A pilot step-wedge cluster randomized trial. Int J Med Informatics 2016;84(5):299-307.

87. Ralph AP, Read C, Johnston V, de Dassel JL, Bycroft K, Mitchell A, et al. Improving delivery of secondary prophylaxis for rheumatic heart disease in remote Indigenous communities: Study protocol for a stepped-wedge randomised trial. Trials 2016;17(1).

88. Rodriguez V, Giuffre C, Villa S, Almada G, Prasopa-Plaizier N, Gogna M, et al. A multimodal intervention to improve hand hygiene in ICUs in Buenos Aires, Argentina: A stepped wedge trial. Int J Qual Health Care 2015;27(5):405-411.

89. Shah L, Rojas M, Mori O, Zamudio C, Kaufman JS, Otero L, et al. Implementation of a stepped-wedge cluster randomized design in routine public health practice: design and application for a tuberculosis (TB) household contact study in a high burden area of Lima, Peru. BMC Public Health 2015;15:587.

90. Suman A, Schaafsma FG, Elders PJM, van Tulder MW, Anema JR. Cost-effectiveness of a multifaceted implementation strategy for the Dutch multidisciplinary guideline for nonspecific low back pain: design of a stepped-wedge cluster randomised controlled trial. BMC Public Health 2015;15:522.

91. Tielsch JM, Katz J, Zeger SL, Khatry SK, Shrestha L, Breysse P, et al. Designs of two randomized, community-based trials to assess the impact of alternative cookstove installation on respiratory illness among young children and reproductive outcomes in rural Nepal. BMC Public Health 2014;14:1271.

92. Tiono AB, Pinder M, N'Fale S, Faragher B, Smith T, Silkey M, et al. The AvecNet Trial to assess whether addition of pyriproxyfen, an insect juvenile hormone mimic, to long-lasting insecticidal mosquito nets provides additional protection against clinical malaria over current best practice in an area with pyrethroid-resistant vectors in rural Burkina Faso: Study protocol for a randomised controlled trial. Trials 2015;16(1).

93. Toftegaard BS, Bro F, Vedsted P. A geographical cluster randomised stepped wedge study of continuing medical education and cancer diagnosis in general practice. Implementation Science 2014;9:159.

94. van Daalen FV, Prins JM, Opmeer BC, Boermeester MA, Visser CE, van Hest RM, et al. A cluster randomized trial for the implementation of an antibiotic checklist based on validated quality indicators: The AB-checklist. BMC Infect Dis 2015;15(1).

95. Var C, Bazzano AN, Srivastav SK, Welty JC, Ek NI, Oberhelman RA. Newborn Infection Control and Care Initiative for health facilities to accelerate reduction of newborn mortality (NICCI): Study protocol for a randomized controlled trial. Trials 2015;16(1).

96. Verberne CJ, Zhan Z, Van Den Heuvel E, Grossmann I, Doornbos PM, Havenga K, et al. Intensified follow-up in colorectal cancer patients using frequent Carcino-Embryonic Antigen (CEA) measurements and CEA-triggered imaging: Results of the randomized "CEAwatch" trial. Eur J Surg Oncol 2015;41(9):1188-1196.

97. Wald HL, Bandle B, Richard AA, Min S, Capezuti E. A Trial of electronic surveillance feedback for quality improvement at Nurses Improving Care for Healthsystem Elders (NICHE) hospitals. Am J Infect Control 2014 Oct;42(10 Suppl):S250-6.

98. Williamson A, Redman S, Haynes A, Barker D, Jorm L, Green S, et al. Supporting Policy In health with Research: an Intervention Trial (SPIRIT)—protocol for a stepped wedge trial. BMJ Open 2014;4(7).

For peer review only

99. Wu M-, Wu C-, Chen B-. Behavioral Intervention and Decreased Daily Melamine Exposure from Melamine Tableware. Environ Sci Technol 2015;49(16):9964-9970.

100. Hoogendijk EO, van der Horst HE, van de Ven PM, Twisk JWR, Deeg DJH, Frijters DHM, et al. Effectiveness of a Geriatric Care Model for frail older adults in primary care: Results from a stepped wedge cluster randomised trial. Eur J Intern Med 2015;28(43).

101. Ononge S, Campbell OMR, Kaharuza F, Lewis JL, Fielding K, Mirembe F. Effectiveness and safety of misoprostol distributed to antenatal women to prevent postpartum haemorrhage after child-births: a stepped-wedge cluster-randomised trial. BMC Pregnancy and Childbirth 2015;15(315).

For peer review only

PRISMA 2009 Checklist

Section/topic	#	Checklist item	Reported on page #
**TITLE**			
Title	1	Identify the report as a systematic review, meta-analysis, or both.	1-2
**ABSTRACT**			
Structured summary	2	Provide a structured summary including, as applicable: background; objectives; data sources; study eligibility criteria, participants, and interventions; study appraisal and synthesis methods; results; limitations; conclusions and implications of key findings; systematic review registration number.	12-35
**INTRODUCTION**			
Rationale	3	Describe the rationale for the review in the context of what is already known.	50-111
Objectives	4	Provide an explicit statement of questions being addressed with reference to participants, interventions, comparisons, outcomes, and study design (PICOS).	111-114
**METHODS**			
Protocol and registration	5	Indicate if a review protocol exists, if and where it can be accessed (e.g., Web address), and, if available, provide registration information including registration number.	NA
Eligibility criteria	6	Specify study characteristics (e.g., PICOS, length of follow-up) and report characteristics (e.g., years considered, language, publication status) used as criteria for eligibility, giving rationale.	117-129
Information sources	7	Describe all information sources (e.g., databases with dates of coverage, contact with study authors to identify additional studies) in the search and date last searched.	117-121
Search	8	Present full electronic search strategy for at least one database, including any limits used, such that it could be repeated.	Supplementary File 1
Study selection	9	State the process for selecting studies (i.e., screening, eligibility, included in systematic review, and, if applicable, included in the meta-analysis).	130-134
Data collection process	10	Describe method of data extraction from reports (e.g., piloted forms, independently, in duplicate) and any processes for obtaining and confirming data from investigators.	136-139
Data items	11	List and define all variables for which data were sought (e.g., PICOS, funding sources) and any assumptions and simplifications made.	140-166
Risk of bias in individual studies	12	Describe methods used for assessing risk of bias of individual studies (including specification of whether this was done at the study or outcome level), and how this information is to be used in any data synthesis.	NA
Summary measures	13	State the principal summary measures (e.g., risk ratio, difference in means).	140-166
Synthesis of results	14	Describe the methods of handling data and combining results of studies, if done, including measures of consistency (e.g., I^2) for each meta-analysis.	167-172

For peer review only

PRISMA 2009 Checklist

Section/topic	#	Checklist item	Reported on page #
Risk of bias across studies	15	Specify any assessment of risk of bias that may affect the cumulative evidence (e.g., publication bias, selective reporting within studies).	NA
Additional analyses	16	Describe methods of additional analyses (e.g., sensitivity or subgroup analyses, meta-regression), if done, indicating which were pre-specified.	NA
**RESULTS**			
Study selection	17	Give numbers of studies screened, assessed for eligibility, and included in the review, with reasons for exclusions at each stage, ideally with a flow diagram.	174-178, Figure 2
Study characteristics	18	For each study, present characteristics for which data were extracted (e.g., study size, PICOS, follow-up period) and provide the citations.	179-260, Table 1
Risk of bias within studies	19	Present data on risk of bias of each study and, if available, any outcome level assessment (see item 12).	NA
Results of individual studies	20	For all outcomes considered (benefits or harms), present, for each study: (a) simple summary data for each intervention group (b) effect estimates and confidence intervals, ideally with a forest plot.	NA
Synthesis of results	21	Present results of each meta-analysis done, including confidence intervals and measures of consistency.	NA
Risk of bias across studies	22	Present results of any assessment of risk of bias across studies (see Item 15).	NA
Additional analysis	23	Give results of additional analyses, if done (e.g., sensitivity or subgroup analyses, meta-regression [see Item 16]).	NA
**DISCUSSION**			
Summary of evidence	24	Summarize the main findings including the strength of evidence for each main outcome; consider their relevance to key groups (e.g., healthcare providers, users, and policy makers).	262-312
Limitations	25	Discuss limitations at study and outcome level (e.g., risk of bias), and at review-level (e.g., incomplete retrieval of identified research, reporting bias).	331-345
Conclusions	26	Provide a general interpretation of the results in the context of other evidence, and implications for future research.	346-375
**FUNDING**			
Funding	27	Describe sources of funding for the systematic review and other support (e.g., supply of data); role of funders for the systematic review.	376-384

From: Moher D, Liberati A, Tetzlaff J, Altman DG, The PRISMA Group (2009). Preferred Reporting Items for Systematic Reviews and Meta-Analyses: The PRISMA Statement. PLoS Med 6(7): e1000097. doi:10.1371/journal.pmed1000097

For more information, visit: www.prisma-statement.org.

For peer review only - http://bmjopen.bmj.com/site/about/guidelines.xhtml
